# Supplementary material for: m-Carborane as a Novel Core for Periphery-Decorated Macromolecules
Source: Molecules. 2020 Jun 18;25(12):2814. doi: 10.3390/molecules25122814 (PMC7356233; doi:10.3390/molecules25122814)
Supplement: Supplementary file 1 [file molecules-25-02814-s001.pdf]

Supplementary Information for

m-Carborane as a Novel Core for Periphery-Decorated Macromolecules

Ines Bennour, Francesc Teixidor, Zsolt Kelemen and Clara Viñas *

Institut de Ciència de Materials de Barcelona (ICMAB-CSIC). Campus UAB, 08193 Bellaterra, Barcelona, Spain; bennourines@ymail.com (I.B.); teixidor@icmab.es (F.T.); kelemen.zsolt@mail.bme.hu (Z.K.)

* Correspondence: clara@icmab.es

Contents

Characterization of 1:7-*closo*-$C_2B_{10}H_{12}$, 1

Figure S1. $^{13}C\{^1H\}$-NMR spectrum

Figure S2. ^1H-NMR spectrum

Figure S3. $^1H\{^{11}B\}$-NMR spectrum

Figure S4. $^{11}B\{^1H\}$-NMR spectrum

Figure S5. ^{11}B-NMR spectrum

Figure S6. The two-dimensional $^{11}B\{^1H\}$-$^{11}B\{^1H\}$ COSY NMR spectra with the assigned boron vertices.

Figure S7. $^1H\{^{11}B\}$-NMR Selective Irradiation spectra

Characterization of 9,10-I_2-1,7-*closo*-$C_2B_{10}H_{10}$, 2

Figure S8. $^{13}C\{^1H\}$-NMR spectrum

Figure S9. ^1H-NMR spectrum

Figure S10. $^1H\{^{11}B\}$-NMR spectrum

Figure S11. $^{11}B\{^1H\}$-NMR spectrum

Figure S12. ^{11}B-NMR spectrum

Figure S13. The two-dimensional $^{11}B\{^1H\}$-$^{11}B\{^1H\}$ COSY NMR spectra with the assigned boron vertices.

Figure S14. $^1H\{^{11}B\}$-NMR Selective Irradiation spectra

Characterization of 9,10-$(CH_2{=}CHCH_2)_2$-1,7-*closo*-$C_2B_{10}H_{10}$, 3

Figure S15. IR-ATR spectrum

Figure S16. $^{13}C\{^1H\}$-NMR spectrum

Figure S17. ^1H-NMR ($CDCl_3$) spectrum

Figure S18. $^1H\{^{11}B\}$-NMR spectrum ($CDCl_3$)

Figure S19. $^{11}B\{^1H\}$-NMR spectrum ($CDCl_3$)

Figure S20. ^{11}B-NMR spectrum ($CDCl_3$)

Figure S21. $^{11}B\{^1H\}$-NMR spectrum ($(CD_3)_2CO$)

Figure S22. ^{11}B-NMR spectrum ($(CD_3)_2CO$)

Figure S23. Superposition of $^{1}H\{^{11}B\}$-NMR and ^{1}H-NMR spectra in ($(CD_3)_2CO$)

Characterization of 9,10-($HOCH_2CH_2CH_2$)$_2$-1,7-*closo*- $C_2B_{10}H_{10}$, 4

Figure S24. IR-ATR spectrum

Figure S25. $^{13}C\{^{1}H\}$-NMR spectrum

Figure S26. ^{1}H-NMR spectrum

Figure S27. $^{1}H\{^{11}B\}$-NMR spectrum

Figure S28. $^{11}B\{^{1}H\}$-NMR spectrum

Figure S29. ^{11}B-NMR spectrum

Figure S30 Crystal packing of the 9,10-($HOCH_2CH_2CH_2$)$_2$-1,7-closo- $C_2B_{10}H_{10}$ structure

Table S1. Bond lengths (Å) for 9,10-($HOCH_2CH_2CH_2$)$_2$-1,7-closo- $C_2B_{10}H_{10}$ structure

Table S2. Bond Angles (°) for 9,10-($HOCH_2CH_2CH_2$)$_2$-1,7-closo-$C_2B_{10}H_{10}$ structure

Characterization of 9,10-($ClCH_2CH_2CH_2$)$_2$-1,7-*closo*-$C_2B_{10}H_{10}$, 5

Figure S31. IR-ATR spectrum

Figure S32. $^{13}C\{^{1}H\}$-NMR spectrum

Figure S33. ^{1}H-NMR spectrum ($(CD_3)_2CO$)

Figure S34. $^{1}H\{^{11}B\}$-NMR spectrum ($(CD_3)_2CO$)

Figure S35. $^{11}B\{^{1}H\}$-NMR spectrum ($(CD_3)_2CO$)

Figure S36. ^{11}B-NMR spectrum ($(CD_3)_2CO$)

Characterization of 9,10-($C_6H_5COOCH_2CH_2CH_2$)$_2$-1,7-*closo*-$C_2B_{10}H_{10}$, 6

Figure S37. IR-ATR spectrum

Figure S38. $^{13}C\{^{1}H\}$-NMR spectrum

Figure S39. ^{1}H-NMR spectrum

Figure S40. $^{1}H\{^{11}B\}$-NMR spectrum

Figure S41. $^{11}B\{^{1}H\}$-NMR spectrum ($(CD_3)_2CO$)

Figure S42. ^{11}B-NMR spectrum ($(CD_3)_2CO$)

Characterization of 9,10-(CH_3-C_6H_4-$SO_3(CH_2)_3)_2$-1,7-$C_2B_{10}H_{10}$, 7

Figure S43. ATR spectrum

Figure S44. $^{13}C\{1H\}$-NMR spectrum

Figure S45. ^{1}H-NMR spectrum

Figure S46. $^{1}H\{^{11}B\}$-NMR spectrum

Figure S47. $^{11}B\{^{1}H\}$-NMR spectrum ($(CD_3)_2CO$)

Figure S48. ^{11}B-NMR spectrum ($(CD_3)_2CO$)

Characterization of 9, 10-($N_3CH_2CH_2CH_2)_2$-1,7-*closo*-$C_2B_{10}H_{10}$, 8

Figure S49. ATR spectrum

Figure S50. $^{13}C\{^{1}H\}$-NMR spectrum

Figure S51. ^{1}H-NMR spectrum

Figure S52. $^{1}H\{^{11}B\}$-NMR spectrum

Figure S53. $^{11}B\{^{1}H\}$-NMR spectrum ($(CD_3)_2CO$)

Figure S54. ^{11}B-NMR spectrum ($(CD_3)_2CO$)

Characterization of 9, 10-($C_6H_5C_2N_3CH_2CH_2CH_2)_2$-1,7-*closo*-$C_2B_{10}H_{10}$, 9

Figure S55. ATR-IR spectrum

Figure S56. $^{13}C\{^{1}H\}$-NMR spectrum

Figure S57. ^{1}H-NMR spectrum

Figure S58. $^{1}H\{^{11}B\}$-NMR spectrum

Figure S59. $^{11}B\{^{1}H\}$-NMR spectrum

Figure S60. ^{11}B-NMR spectrum

Characterization of 9,10-(CH_2=$CHCH_2)_2$-1,7-(CH_2=$CHCH_2)_2$-*closo*-$C_2B_{10}H_8$, 10

Figure S61. $^{13}C\{^{1}H\}$-NMR spectrum

Figure S62. ^{1}H-NMR spectrum

Figure S63. $^{1}H\{^{11}B\}$-NMR spectrum

Figure S64. $^{11}B\{^{1}H\}$-NMR spectrum

Figure S65. ^{11}B-NMR spectrum

Figure S66. Comparison of ^{1}H-NMR spectrum of 3 and 10

Characterization of 9,10-$(HOCH_2CH_2CH_2)_2$-1,7-$(HOCH_2CH_2CH_2)_2$-*closo*-$C_2B_{10}H_8$, 11

Figure S67. $^{13}C\{^{1}H\}$-NMR spectrum:

Figure S68. ^{1}H-NMR spectrum

Figure S69. $^{1}H\{^{11}B\}$-NMR spectrum

Figure S70. $^{11}B\{^{1}H\}$-NMR spectrum

Figure S71. ^{11}B-NMR spectrum

Characterization of 9,10-$(CH_3CHCH)_2$-1,7-*closo*-$C_2B_{10}H_{10}$, 12

Figure S72. IR-ATR spectrum

Figure S73. ^{1}H-NMR spectrum

Figure S74. $^{1}H\{^{11}B\}$-NMR spectrum

Figure S75. $^{11}B\{^{1}H\}$-NMR spectrum ($CDCl_3$)

Figure S76. ^{11}B-NMR spectrum ($CDCl_3$)

Figure S77. $^{11}B\{^{1}H\}$-NMR spectrum ($CDCOCD_3$)

Figure S78. ^{11}B-NMRspectrum ($CDCOCD_3$)

Figure S79. Crystal packing of the 9,10-$(CH_3CH{=}CH)_2$-1,7-closo-$C_2B_{10}H_{10}$

Table S3. Bond lengths (Å) for 9,10-$(CH_3CH{=}CH)_2$-1,7-closo-$C_2B_{10}H_{10}$

Table S4. Bond angles (°) for 9,10-$(CH_3CH{=}CH)_2$-1,7-closo-$C_2B_{10}H_{10}$

Theoretical calculations

XYZ coordinates and total energies of the investigated systems

Table S5. o-carborane

Table S6. m-carborane

Table S7. compound 3

Table S8. compound 3 deprotonated at the allylic position.

Table S9. compound 3 deprotonated at cluster carbon atom position.

Table S10. o-carborane analogue of compound 3 deprotonated at the allylic position.

Table S11. o-carborane analogue of compound 3 deprotonated at the cluster carbon atom position.

Figure S80. Two-dimensional fingerprint plots overall plot and those delineated into (a) H…H and O…H/H…O and O…O for 4; (b) H…H and π…H/H…π for 12.

References

Characterization of 1,7-*closo*-$C_2B_{10}H_{12}$, 1, in d6-acetone.

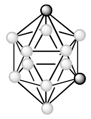

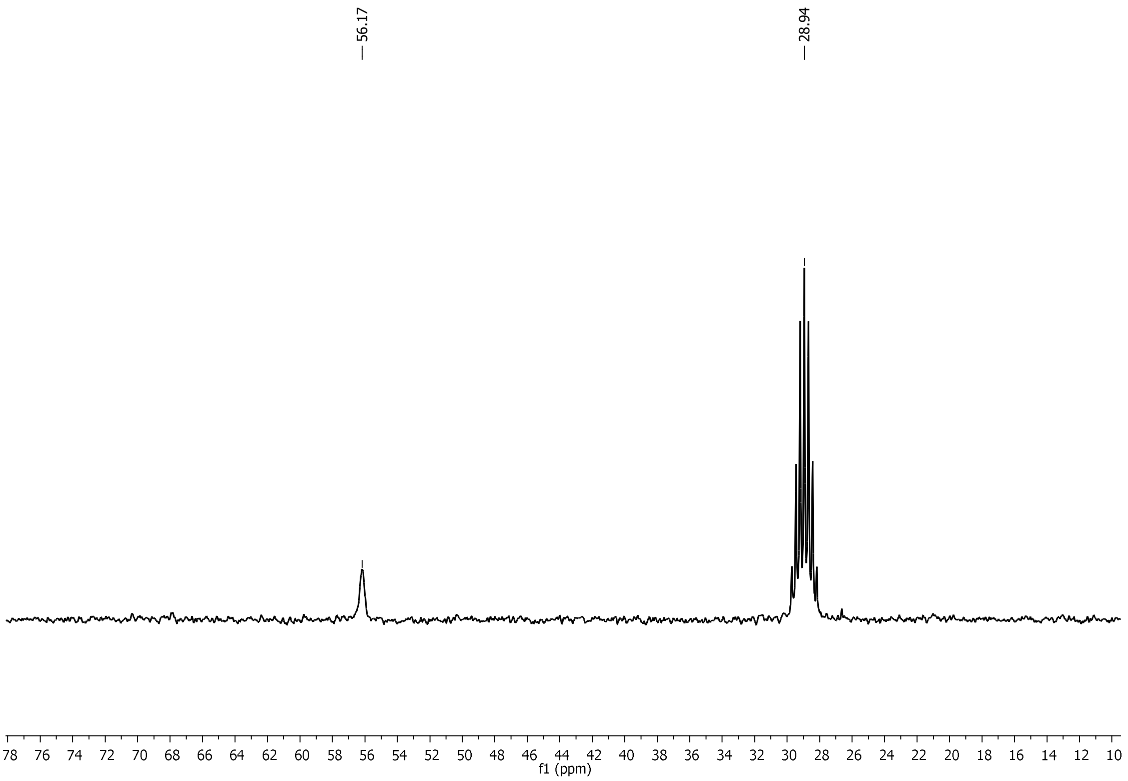

Figure S1. $^{13}C\{^{1}H\}$-NMR spectrum.

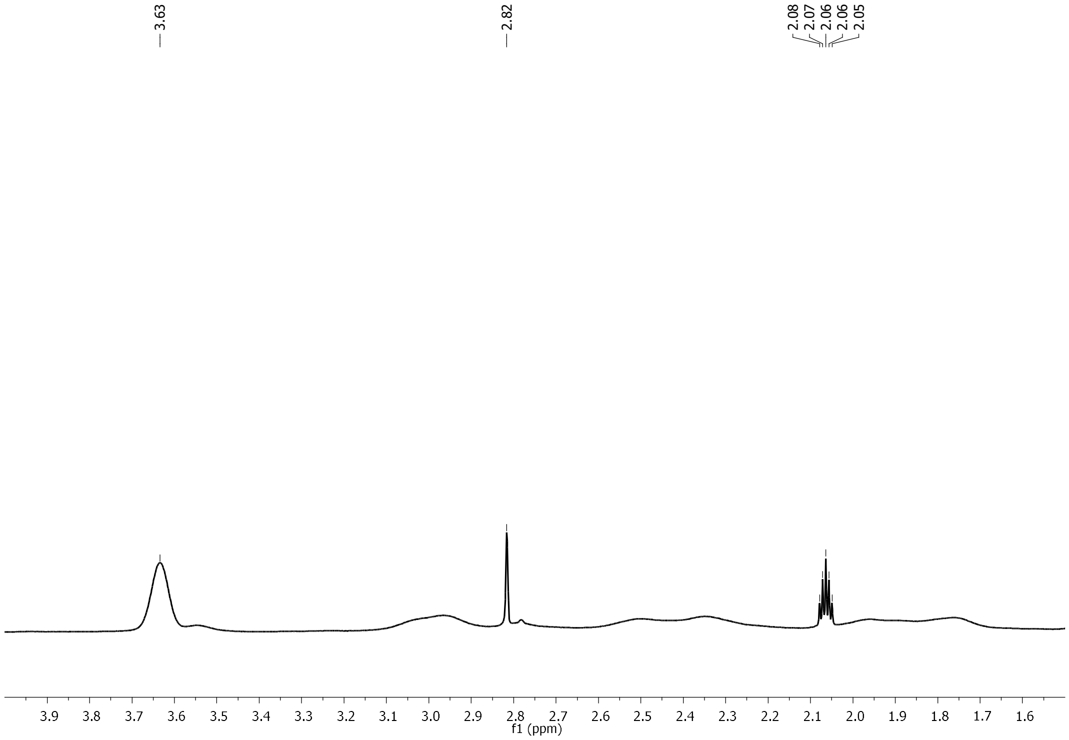

Figure S2. ^1H-NMR spectrum.

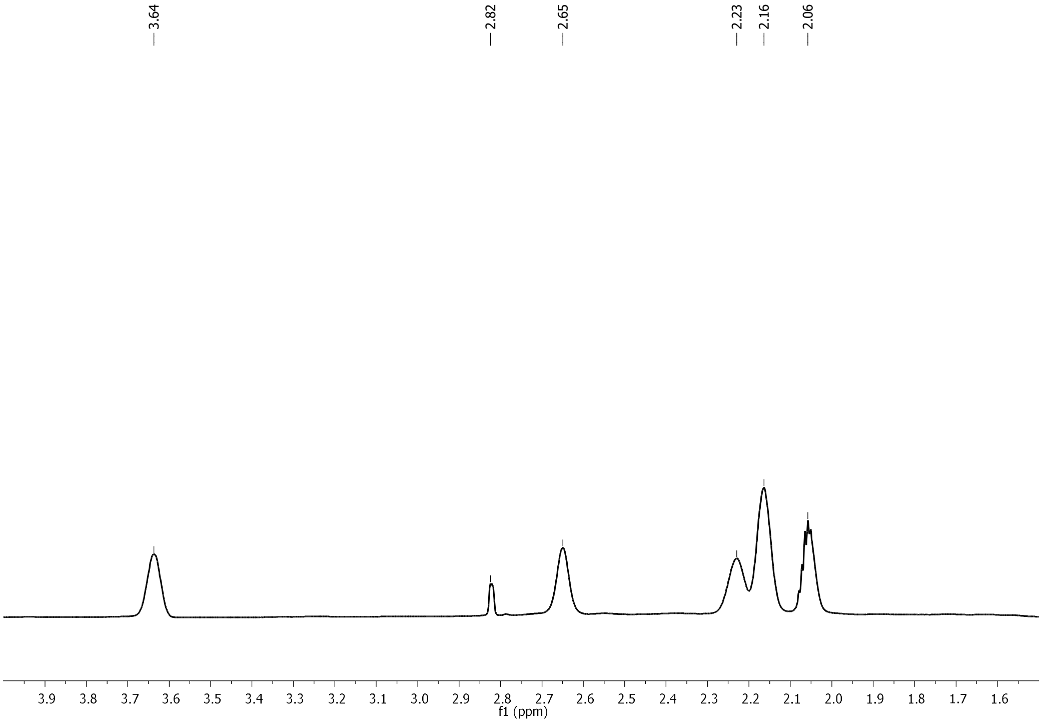

Figure S3. $^1H\{^{11}B\}$-NMR spectrum.

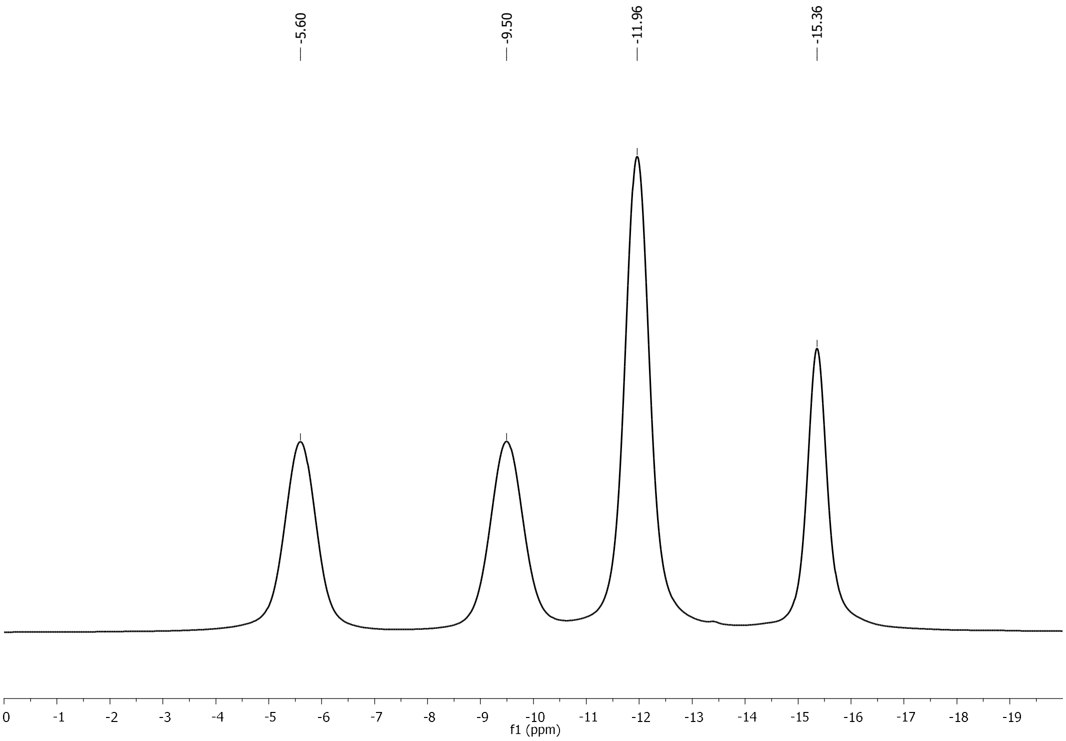

Figure S4. 11B{1H}-NMR spectrum.

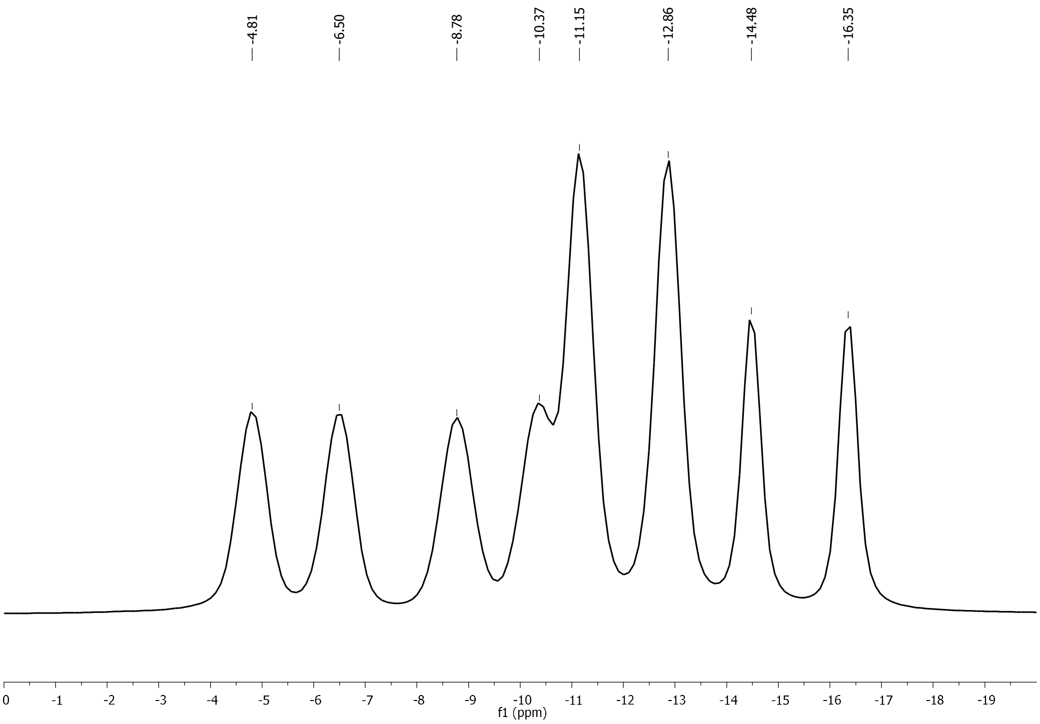

Figure S5. ^{11}B-NMR spectrum

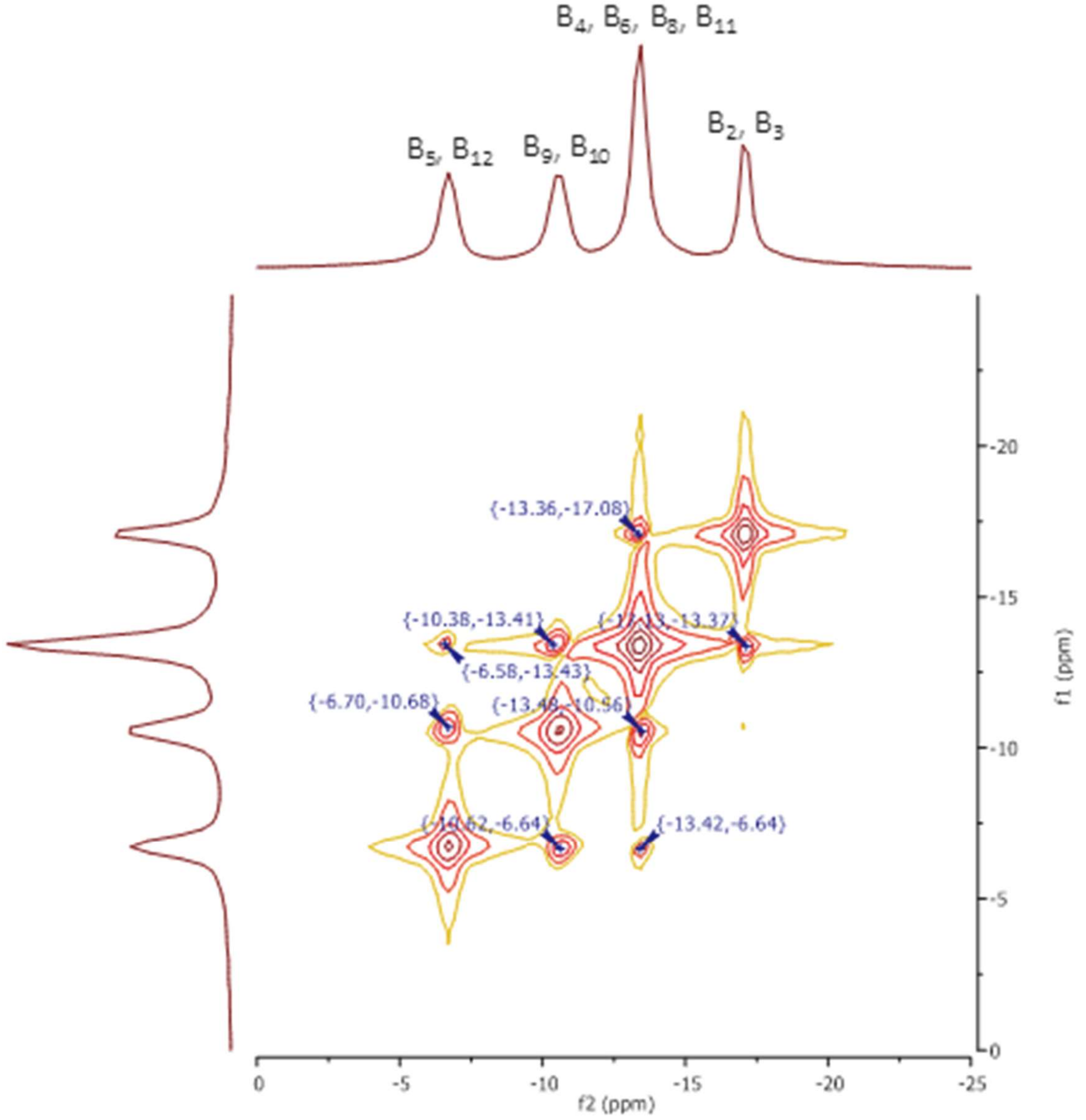

Figure S6. The two-dimensional ^{11}B {^1H}-^{11}B {^1H} COSY NMR spectra with the assigned boron vertices.

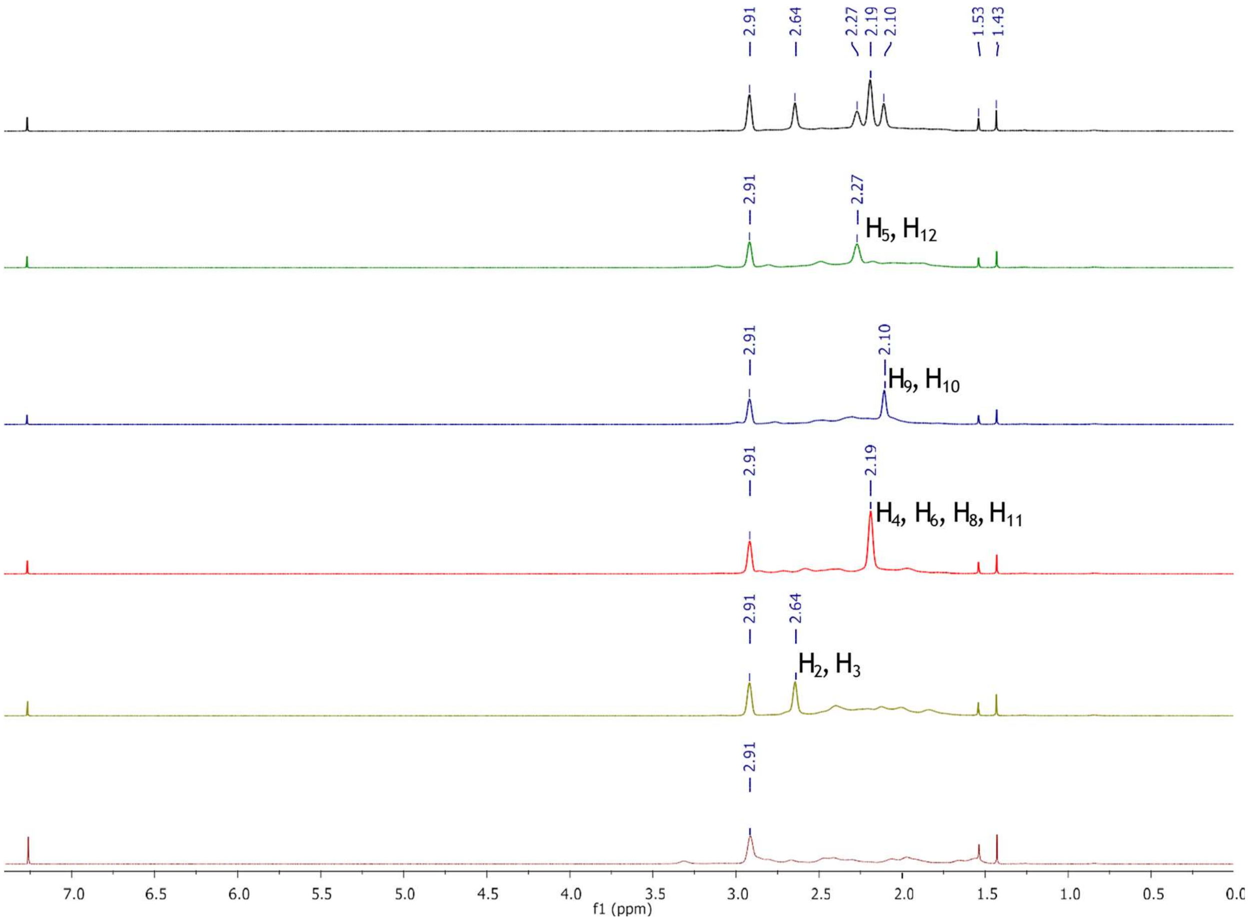

Figure S7. $^{1}H\{^{11}B\}$-NMR Selective Irradiation spectra

Characterization of 9,10-I_2-1,7-*closo*-$C_2B_{10}H_{10}$: 2, in d6-acetone.

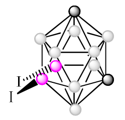

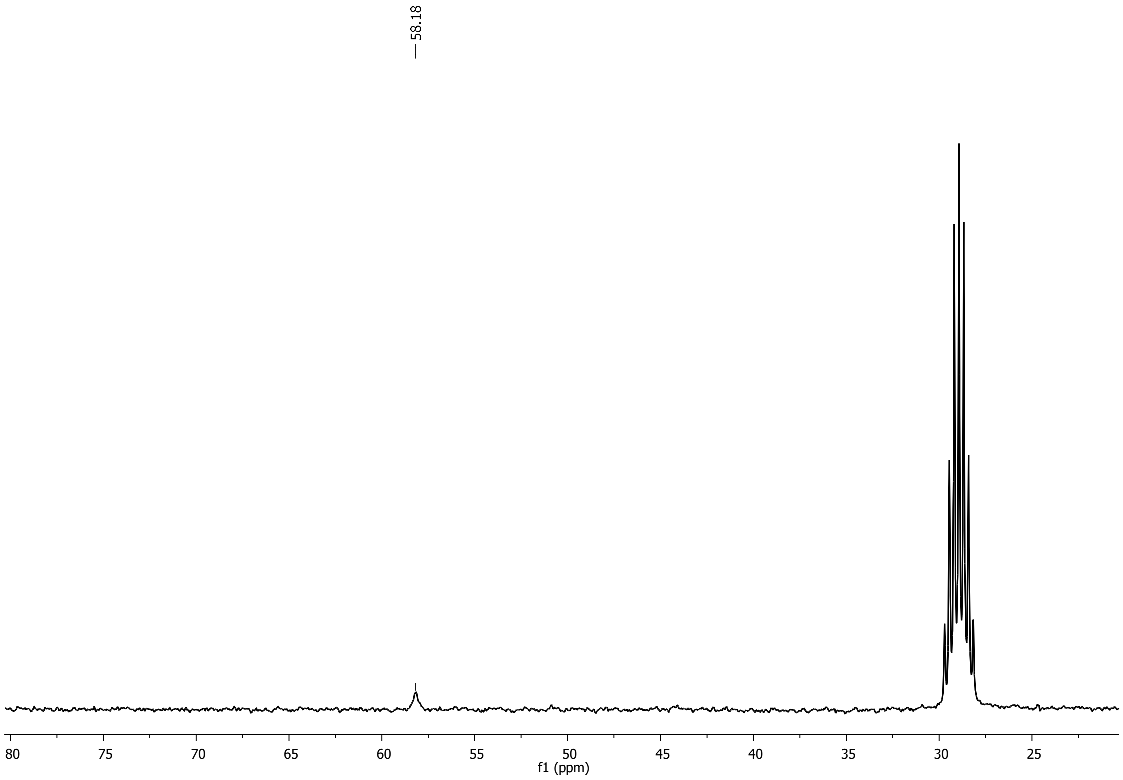

Figure S8. $^{13}C\{^1H\}$-NMR spectrum.

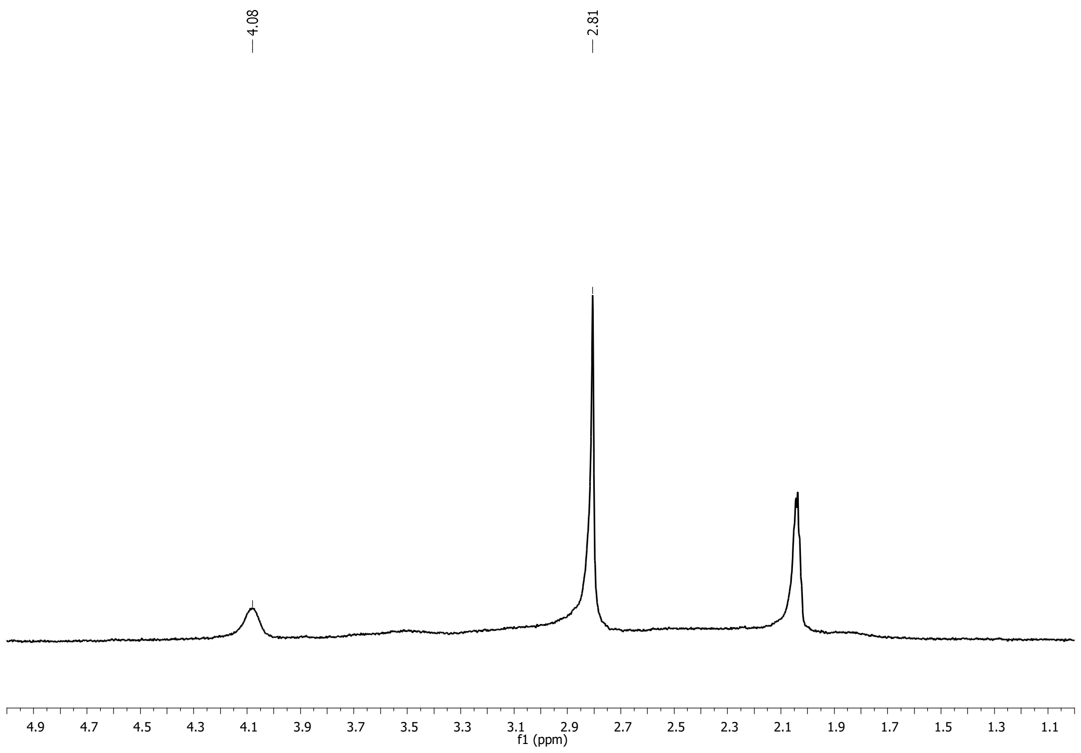

Figure S9. ^{1}H-NMR spectrum

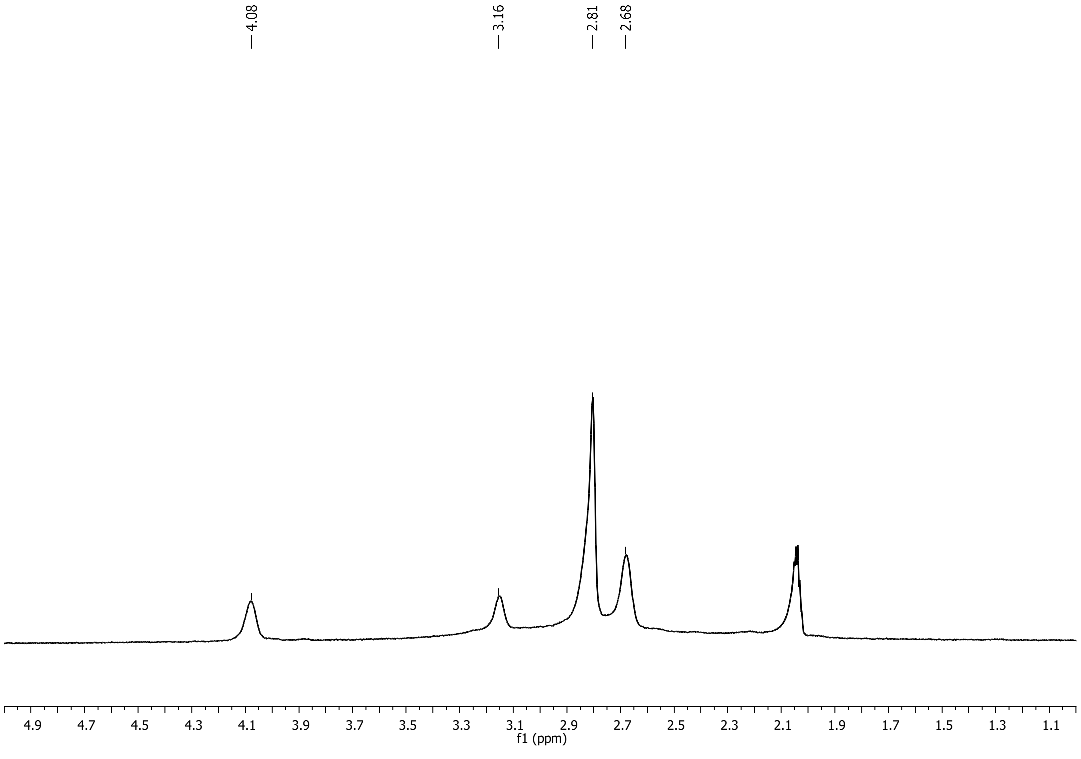

Figure S10. $^{1}H\{^{11}B\}$-NMR spectrum.

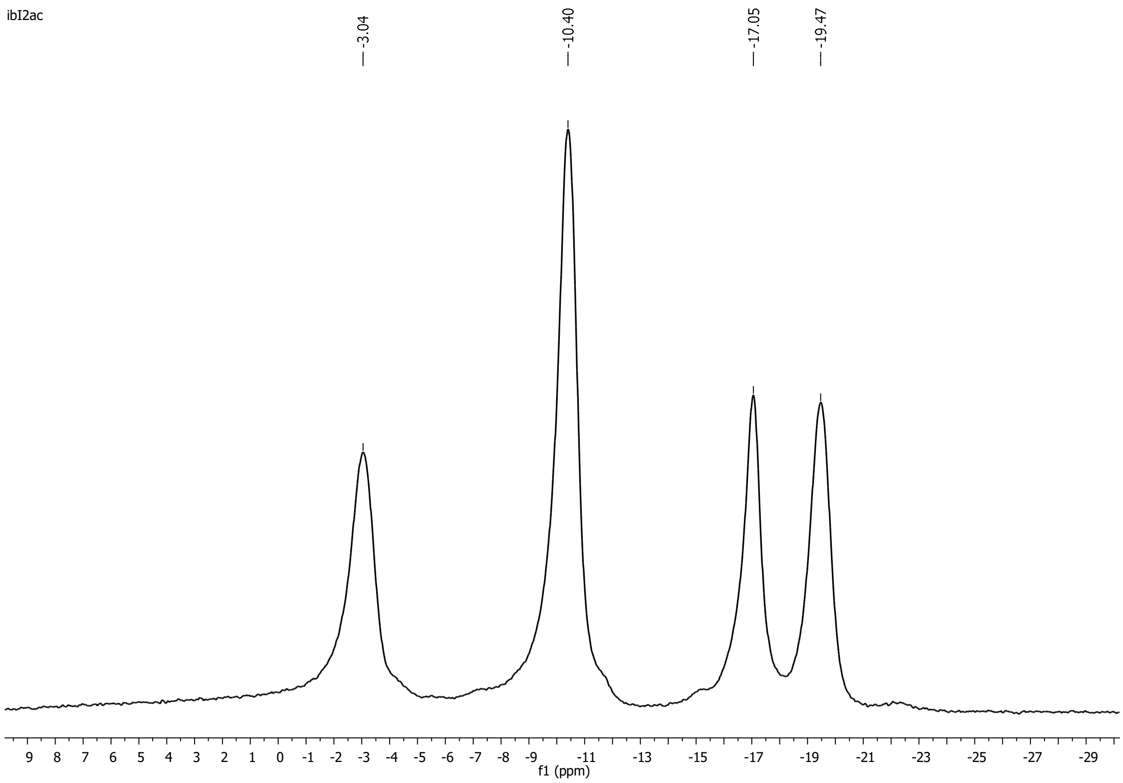

Figure S11. $^{11}B\{^{1}H\}$-NMR spectrum:.

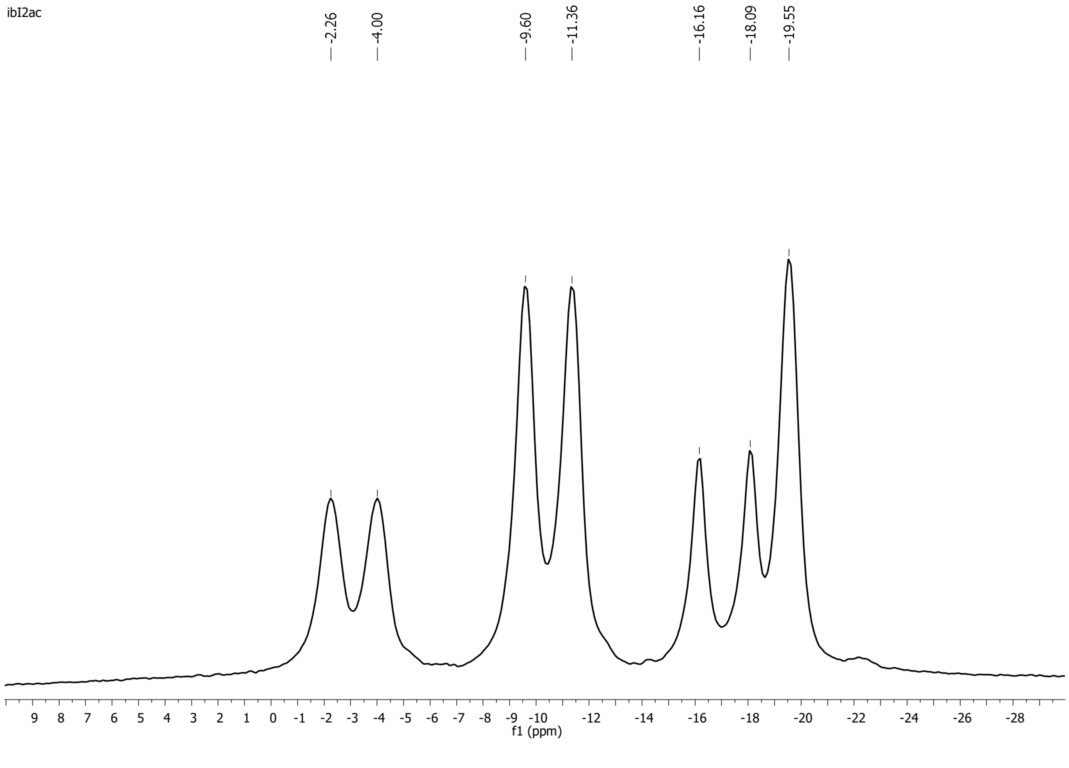

Figure S12. ^{11}B-NMR spectrum

.

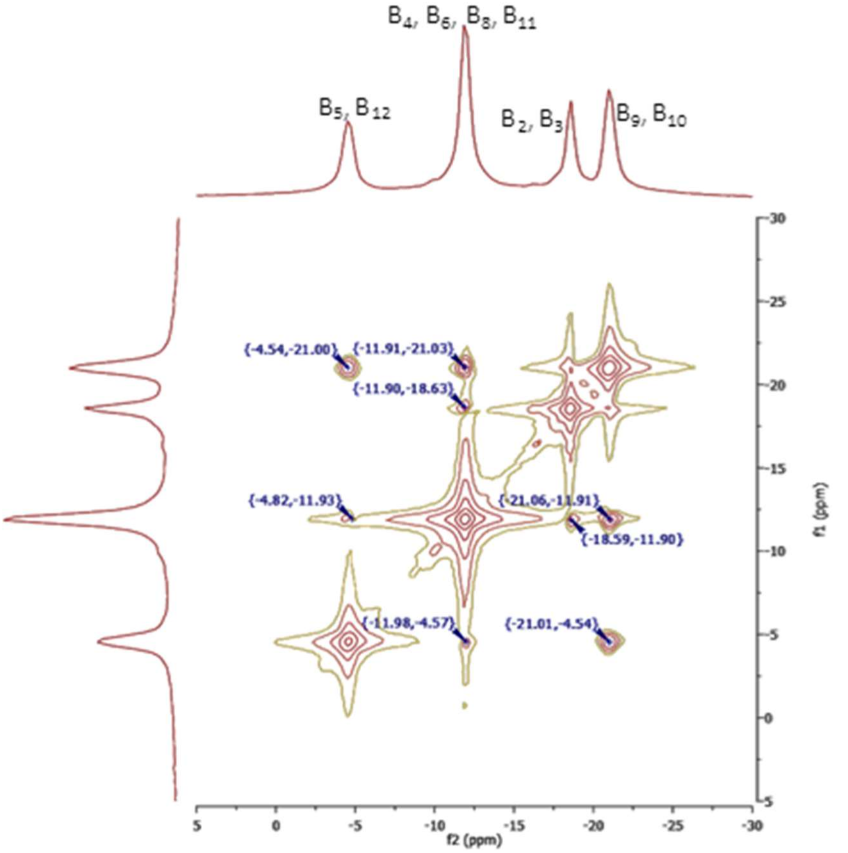

Figure S13. The two-dimensional ^{11}B {^{1}H}-^{11}B {^{1}H} COSY NMR spectra with the assigned boron vertices.

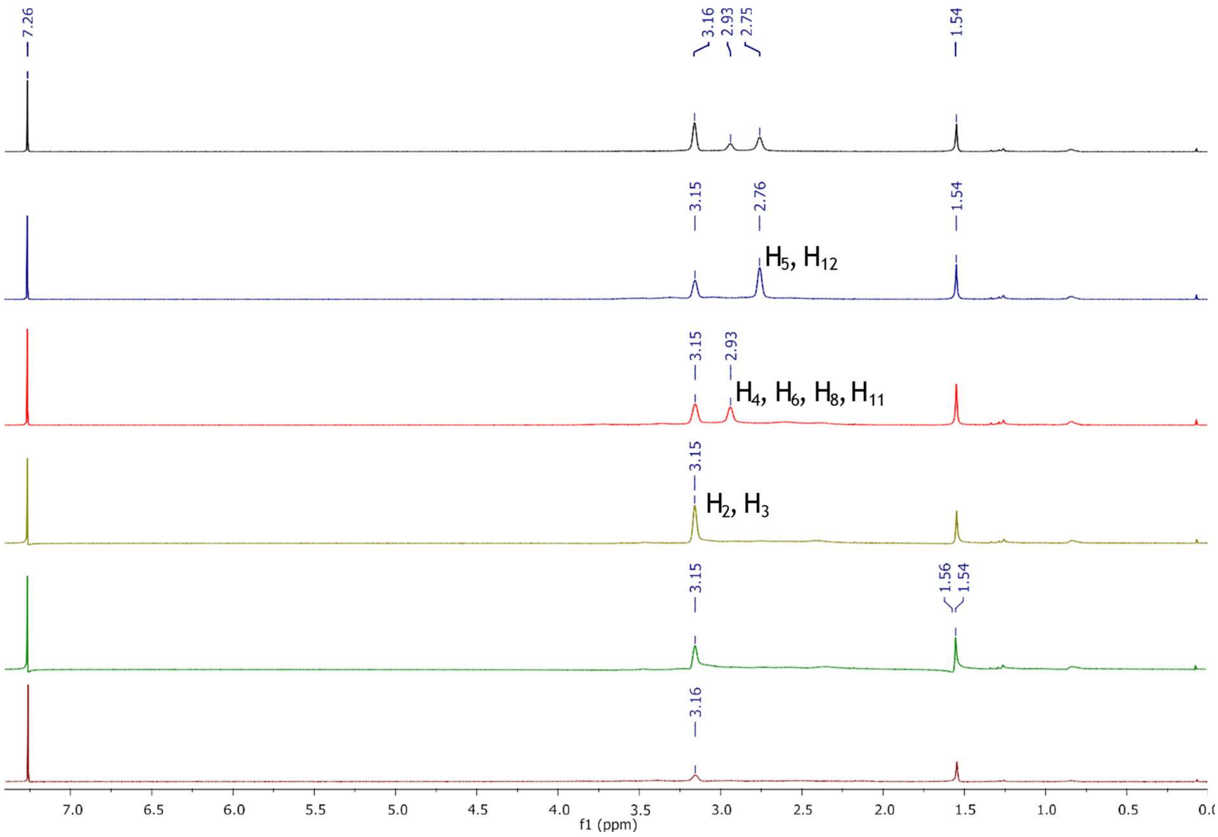

Figure S14. $^{1}H\{^{11}B\}$-NMR Selective Irradiation spectra.

Characterization of 9,10-(CH_2=$CHCH_2$)$_2$-1,7-*closo*-$C_2B_{10}H_{10}$, 3, in $CDCl_3$.

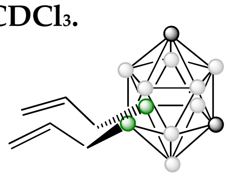

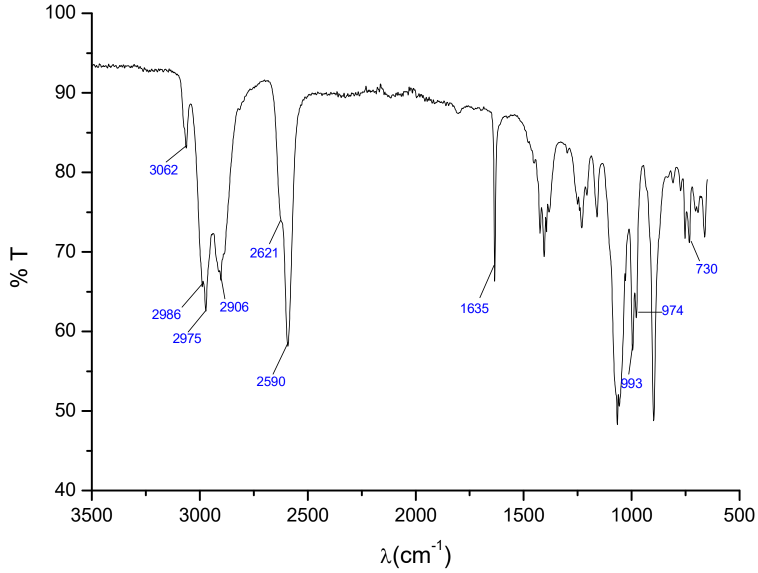

Figure S15. IR-ATR spectrum.

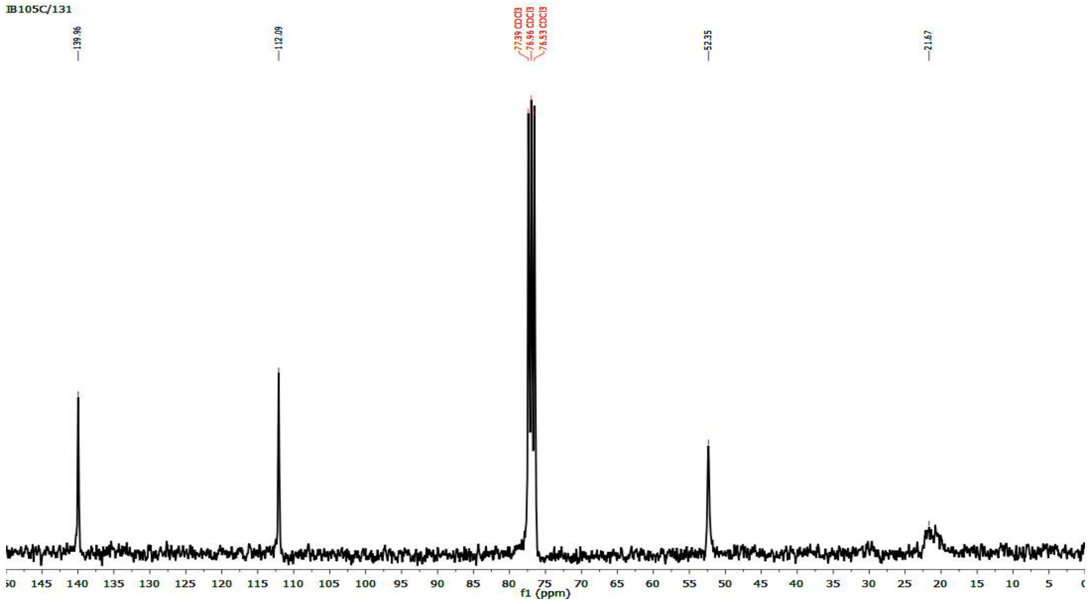

Figure S16. $^{13}C\{^1H\}$-NMR spectrum.

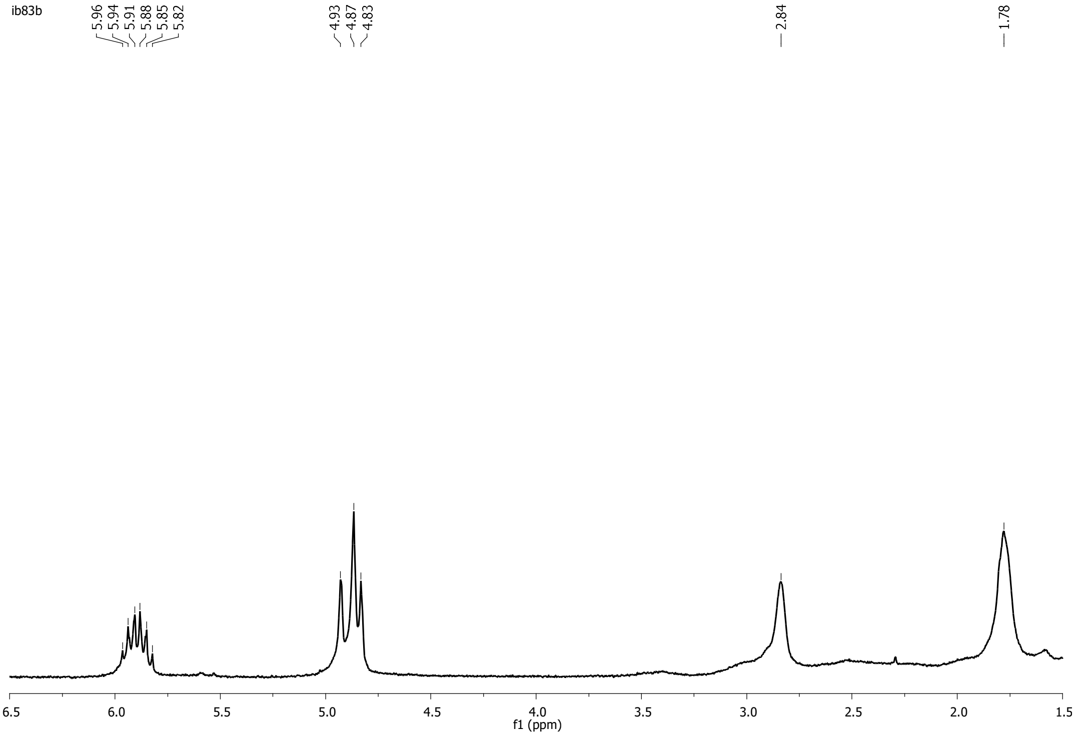

Figure S17. ^{1}H-NMR ($CDCl_3$) spectrum.

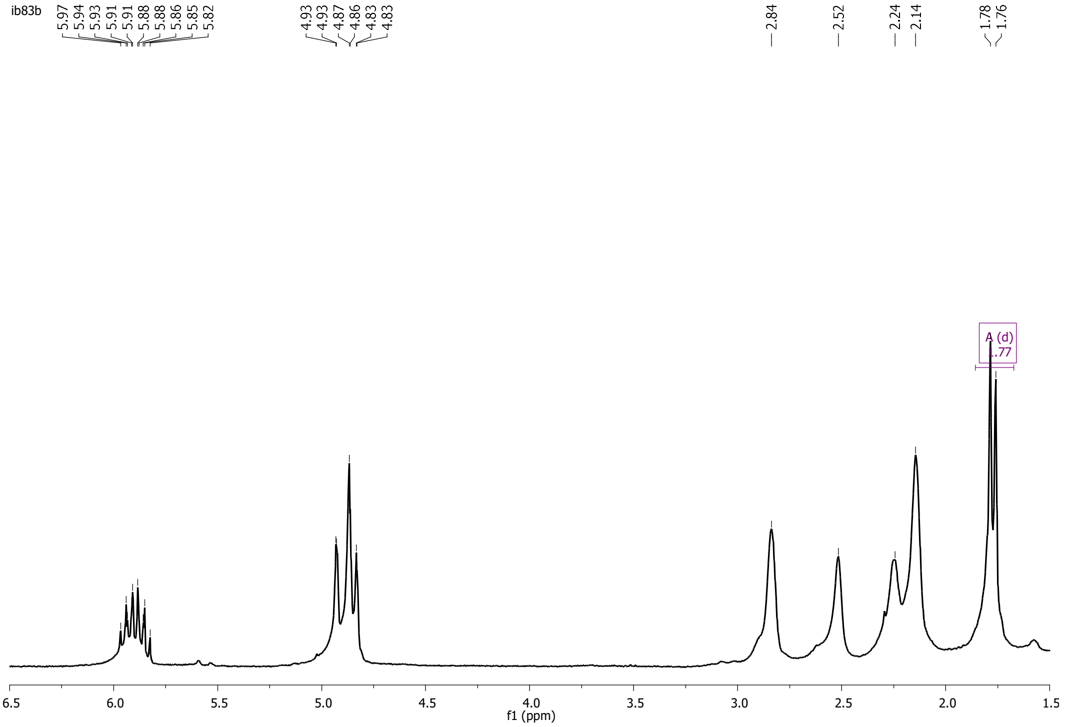

Figure S18. ^{1}H{1^{1}B}-NMR spectrum ($CDCl_3$).

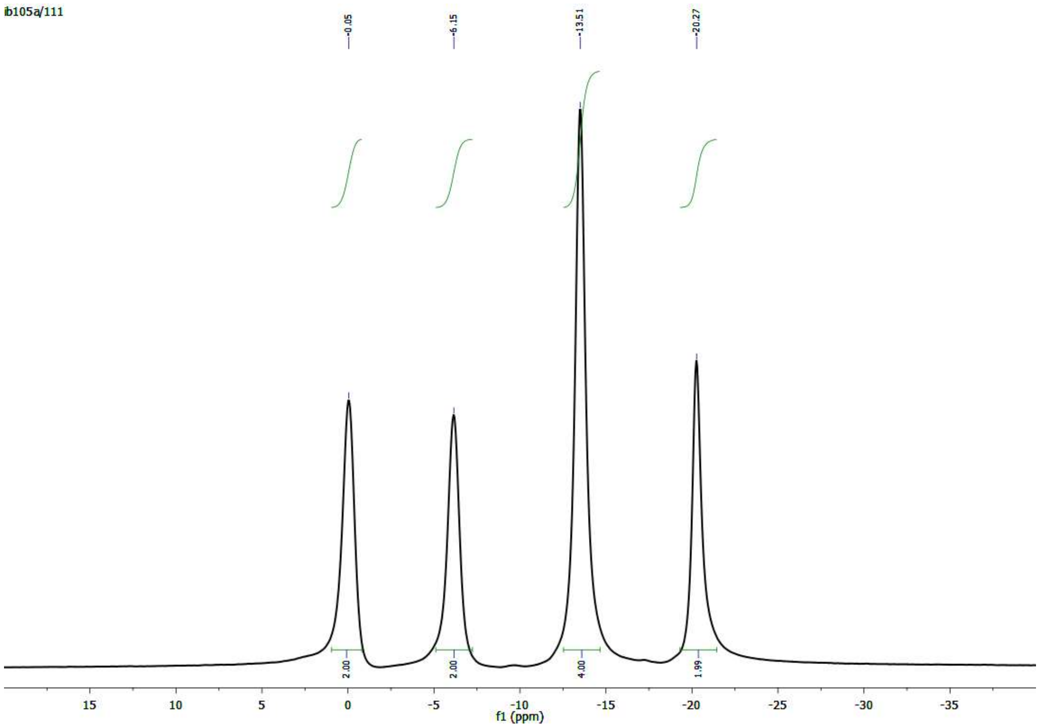

Figure S19. $^{11}B\{^1H\}$-NMR spectrum ($CDCl_3$).

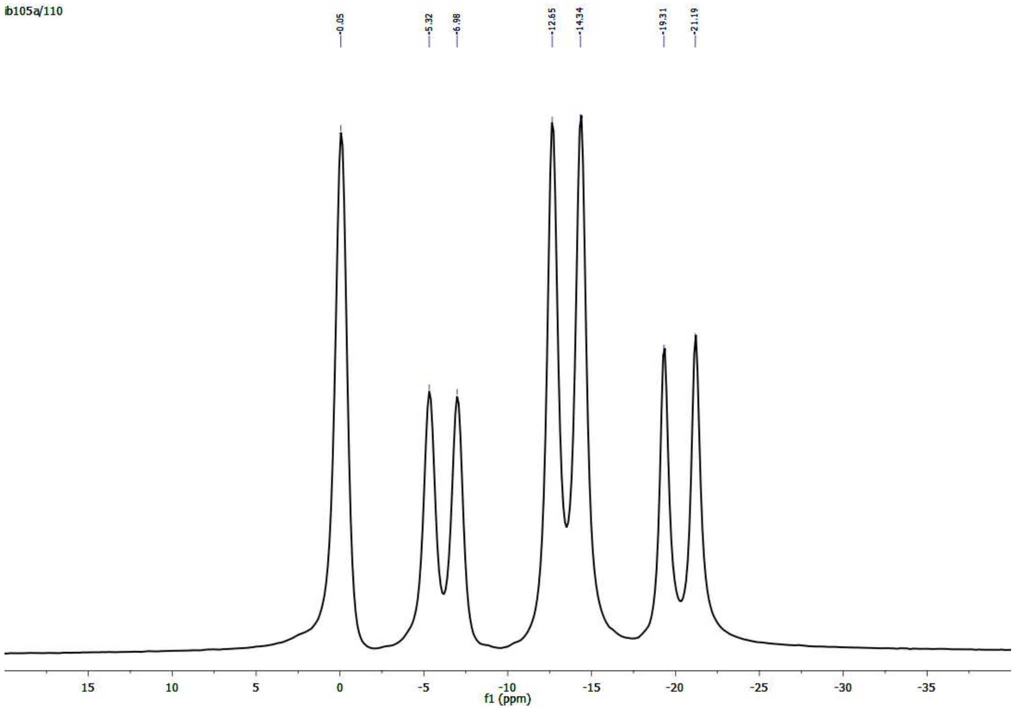

Figure S20. ^{11}B-NMR spectrum ($CDCl_3$)

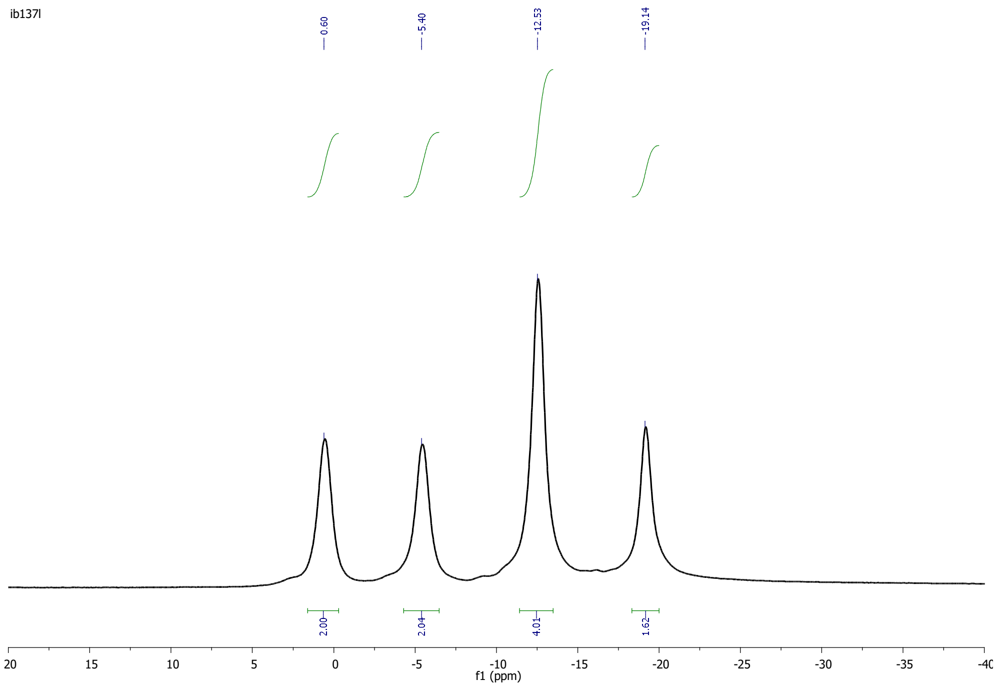

Figure S21. $^{11}B\{^{1}H\}$-NMR spectrum ($(CD_3)_2CO$).

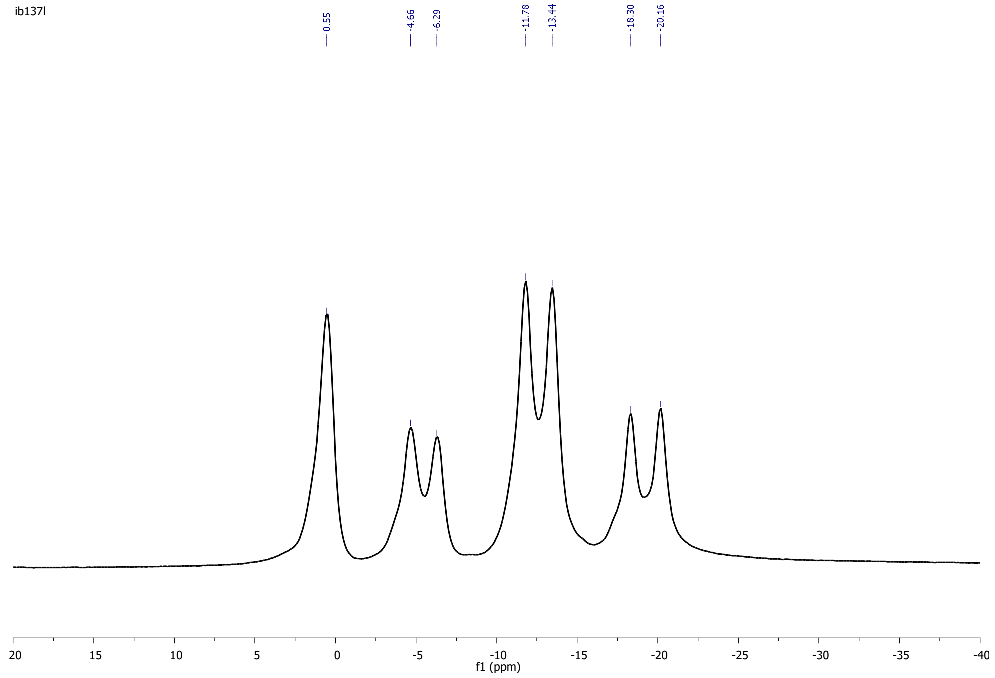

Figure S22. ^{11}B-NMR spectrum ($(CD_3)_2CO$).

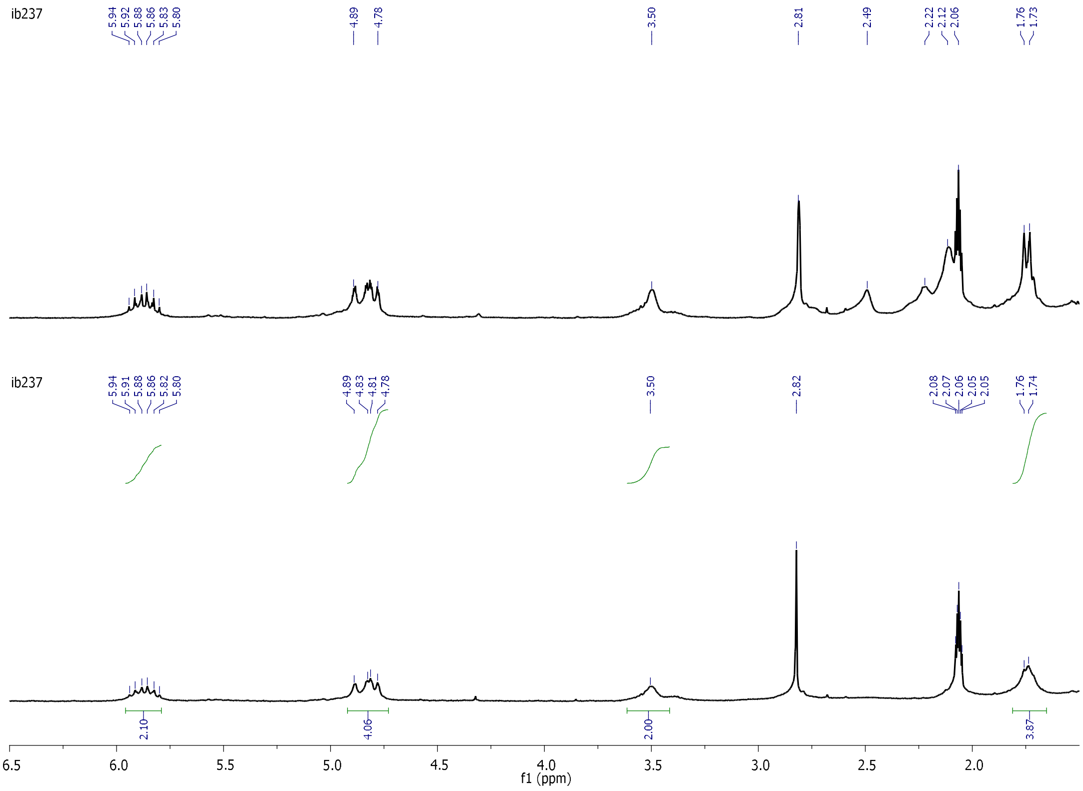

Figure S23. Superposition of $^1H\{^{11}B\}$-NMR and ^1H-NMR spectra in $((CD_3)_2CO)$.

Characterization of 9,10-$(HOCH_2CH_2CH_2)_2$-1,7-*closo*-$C_2B_{10}H_{10}$, 4, in d6-acetone.

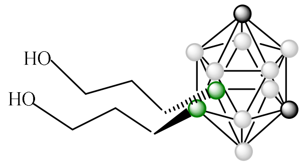

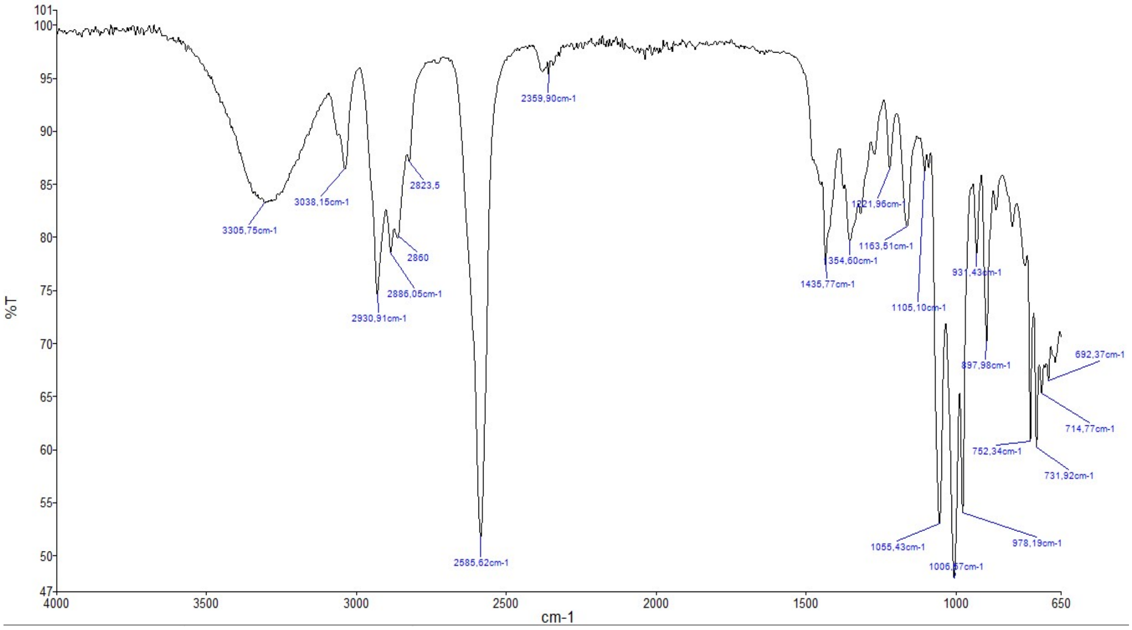

Figure S24. IR-ATR spectrum.

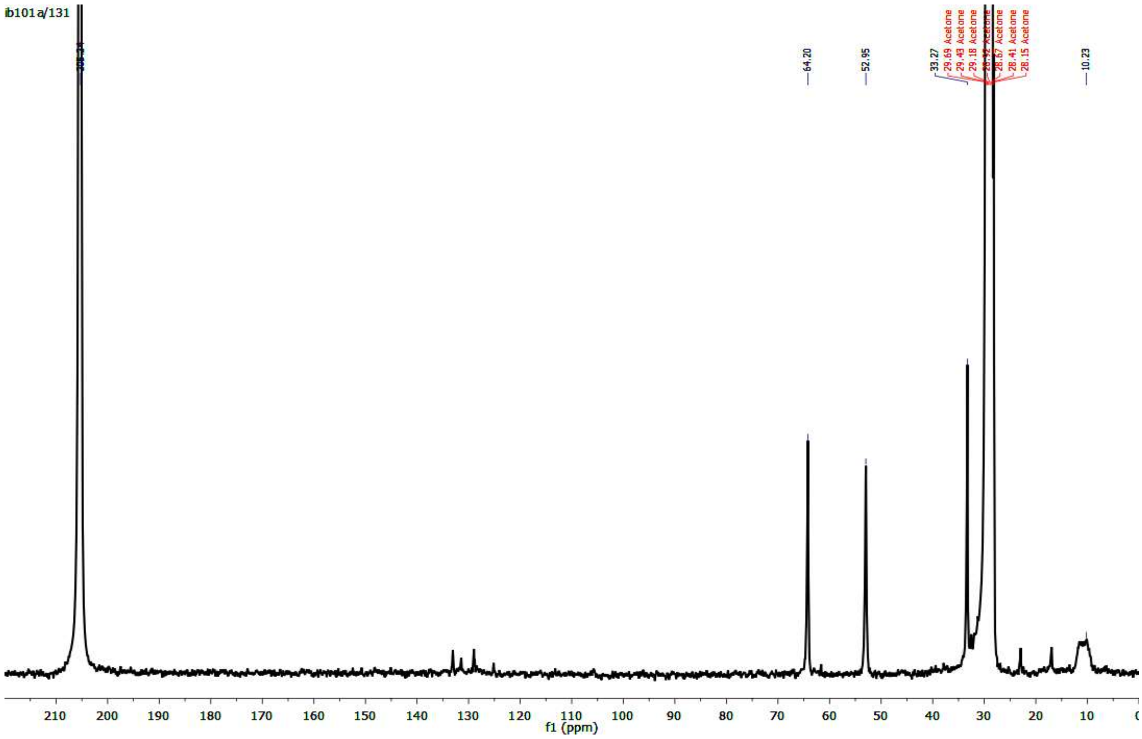

Figure S25. $^{13}C\{^1H\}$-NMR spectrum.

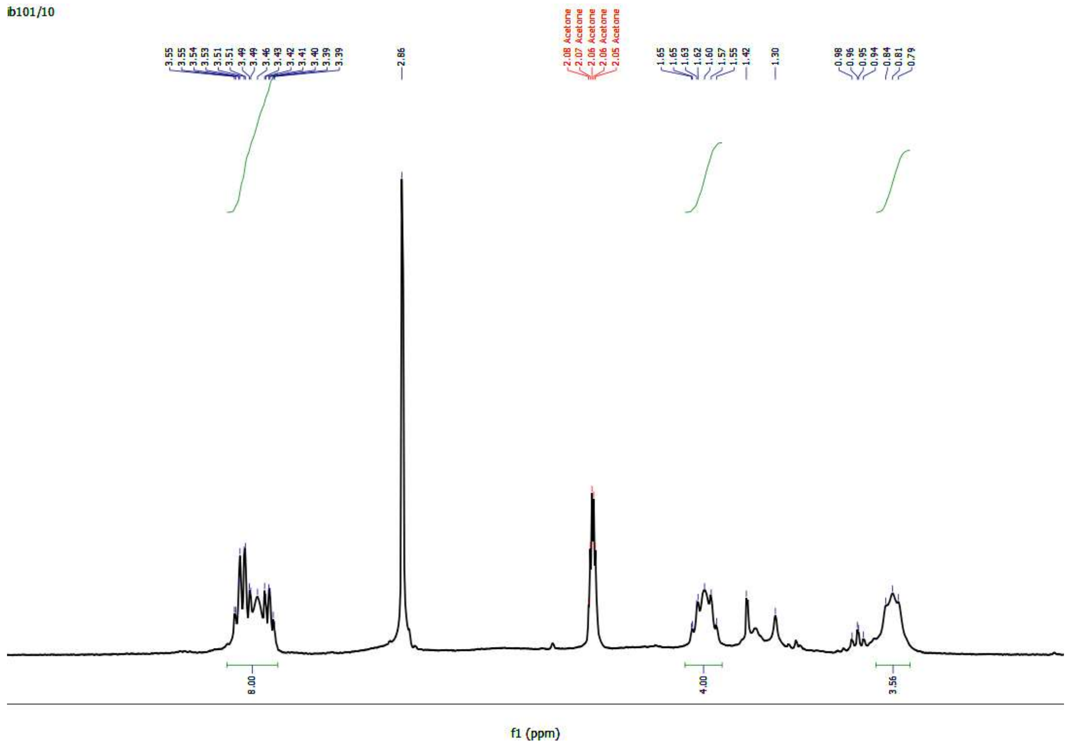

Figure S26. ^{1}H-NMR spectrum.

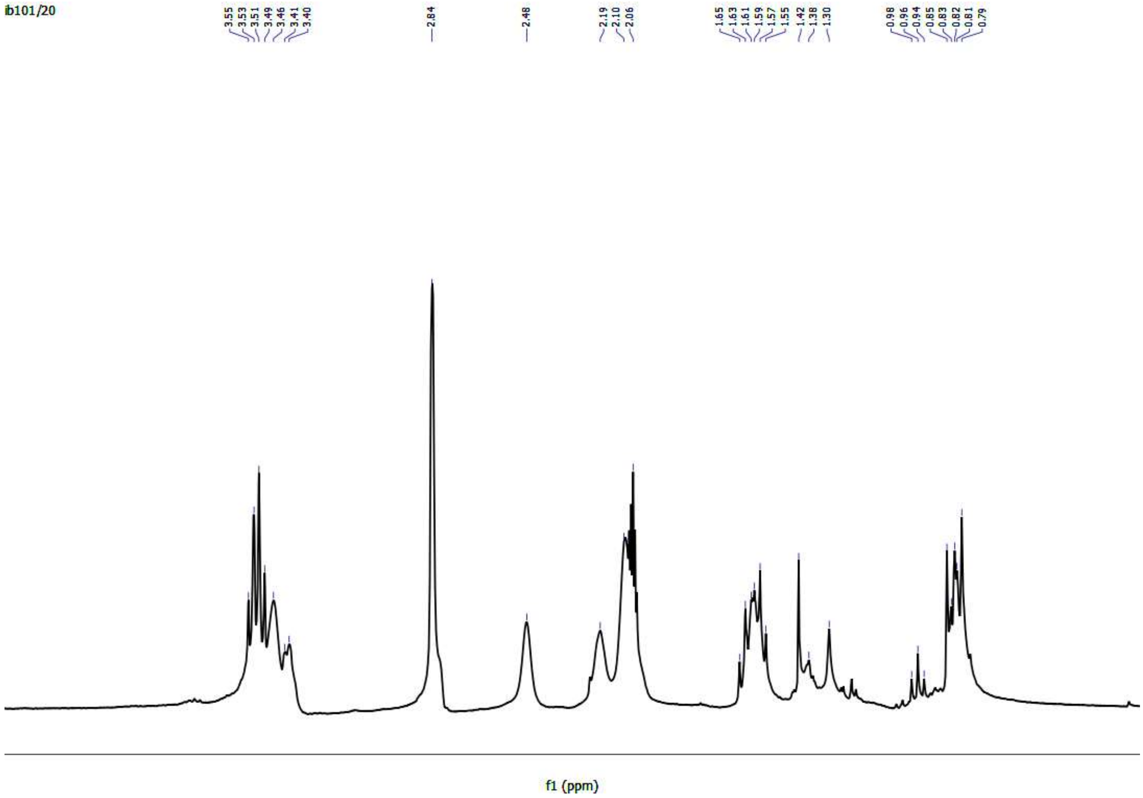

Figure S27. $^{1}H\{^{11}B\}$-NMR spectrum.

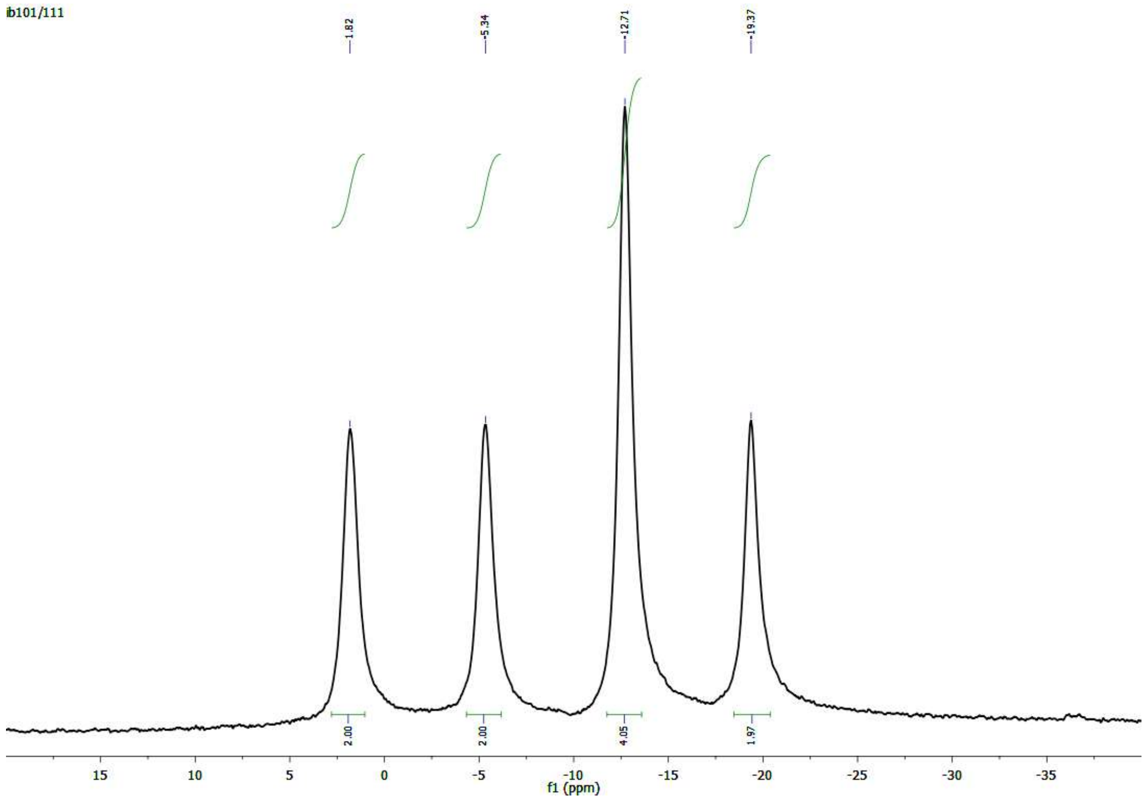

Figure S28. $^{11}B\{^1H\}$-NMR spectrum.

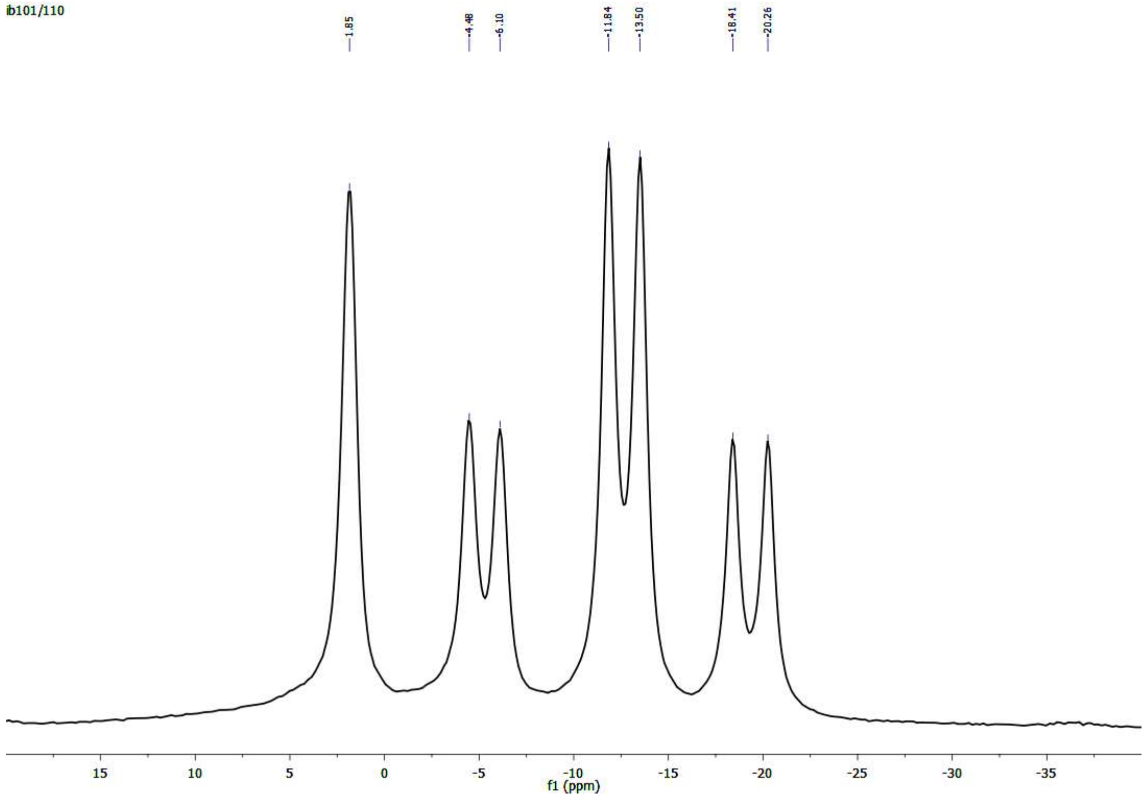

Figure S29. ^{11}B-NMR spectrum.

.

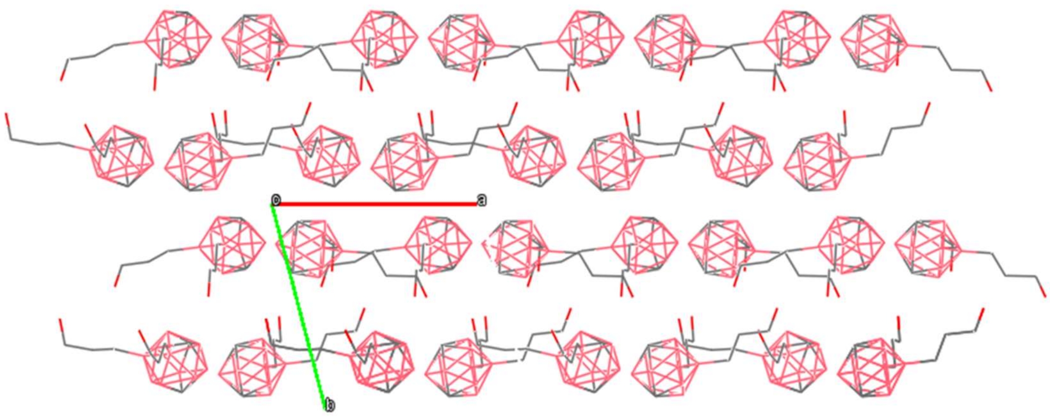

Figure S30. Crystal packing of the 9,10-($HOCH_2CH_2CH_2$)$_2$-1,7-*closo*-$C_2B_{10}H_{10}$ structure.

Table S1. Bond lengths (Å) for 9,10-$(HOCH_2CH_2CH_2)_2$-1,7-*closo*-$C_2B_{10}H_{10}$ structure.

O16-C15	1.446(12)	B9B-B8B	1.833(16)
O20-C19	1.386(16)	O16A-C15A	1.482(17)
C1-B5	1.679(18)	O20A-C19A	1.370(13)
C1-B4	1.70(2)	C1A-B2A	1.62(3)
C1-B2	1.74(2)	C1A-B5A	1.672(19)
C7-B5	1.65(2)	C1A-B3A	1.73(2)
C7-B12	1.704(14)	C7A-B3A	1.60(2)
C7-B8	1.724(19)	C7A-B12A	1.684(15)
C13-C14	1.485(14)	C7A-B2A	1.728(17)
C14-C15	1.519(13)	C13A-C14A	1.470(17)
C17-B9	1.64(2)	C14A-C15A	1.50(2)
B5-B6	1.729(19)	C17A-C18A	1.550(14)
B5-B10	1.780(16)	B5A-B6A	1.742(18)
B4-B8	1.744(18)	B5A-B9A	1.786(15)
B3-B2	1.87(3)	B4A-B3A	1.77(3)
B2-B11	1.74(2)	B3A-B8A	1.768(19)
B6-B11	1.772(17)	B2A-B6A	1.73(2)
B10-B11	1.776(15)	B6A-B10A	1.780(16)
B10-B9	1.857(15)	B10A-B12A	1.801(16)
B9-B12	1.807(15)	B10A-B11A	1.82(2)
C1C-B6C	1.691(17)	B9A-B12A	1.824(17)
C1C-B2C	1.71(2)	C1-B3	1.688(18)
C1C-B3C	1.735(17)	C1-B6	1.707(19)
C7C-B8C	1.63(2)	C7-B3	1.695(19)
C7C-B12C	1.684(16)	C7-B11	1.708(16)
C7C-B6C	1.76(2)	C13-B10	1.583(14)
C13C-C14C	1.509(13)	C17-C18	1.41(2)
C14C-C15C	1.496(14)	C18-C19	1.47(2)
C17C-B9C	1.631(17)	B2-B3	1.753(16)
C19C-O20C	1.331(15)	B5-B9	1.799(19)
B5C-B6C	1.71(2)	B4-B3	1.73(3)
B5C-B10C	1.773(16)	B4-B9	1.75(2)
B4C-B3C	1.778(18)	B3-B8	1.73(2)
B3C-B2C	1.76(2)	B2-B6	1.80(2)
B2C-B6C	1.84(2)	B6-B7	1.751(16)
B10C-B12C	1.739(17)	B10-B12	1.799(13)
B10C-B9C	1.781(16)	B9-B8	1.771(17)
B9C-B8C	1.81(2)	B8-B12	1.753(16)
O16B-C15B	1.423(11)	B11-B12	1.774(15)
O20B-C19B	1.368(16)	C1C-B5C	1.704(17)
C1B-B3B	1.670(18)	C1C-B4C	1.708(17)
C1B-B6B	1.682(17)	C7C-B5C	1.656(17)
C1B-B4B	1.719(16)	C7C-B11C	1.75(2)
C7B-B8B	1.64(2)	C13C-B7C	1.638(15)
C7B-B12B	1.718(14)	C15C-O16C	1.428(17)
C7B-B6B	1.75(2)	C17C-C18C	1.434(18)
C13B-C14B	1.556(13)	C18C-C19C	1.538(14)
C14B-C15B	1.529(11)	B5C-B11C	1.755(19)
C17B-B8B	1.553(15)	B5C-B4C	1.807(18)
B5B-B6B	1.710(19)	B4C-B9C	1.74(2)

B5B-B4B	1.776(17)	B4C-B10C	1.781(15)
B4B-B9B	1.781(13)	B3C-B8C	1.758(19)
B3B-B8B	1.770(16)	B3C-B9C	1.776(18)
B2B-B8B	1.80(2)	B2C-B8C	1.74(2)
B6B-B11B	1.770(16)	B10C-B11C	1.757(16)
B10B-B12B	1.765(13)	B9C-B12C	1.740(18)
B10B-B11B	1.810(15)	B8C-B12C	1.770(18)

Table S2. Bond Angles (°) for 9,10-($HOCH_2CH_2CH_2$)$_2$-1,7-*closo*-$C_2B_{10}H_{10}$ structure.

B2-C1-B4	114.0(10)	B9C-B4C-B8C	61.8(8)
B4-C1-B3	61.4(9)	C1C-B4C-B3C	58.1(7)
B4-C1-B6	117.3(10)	B5C-B4C-B3C	107.5(9)
B2-C1-B5	114.2(10)	C10C-B5C-B9C	57.2(8)
B3-C1-B5	116.0(10)	C10C-B5C-B4C	103.6(10)
B5-C10-B12	115.2(9)	B9C-B5C-B4C	60.3(8)
B5-C10-B11	62.2(9)	C1C-B5C-B6C	56.8(8)
B12-C10-B11	62.6(6)	B4C-B5C-B6C	107.8(11)
B4-C10-B9	60.7(9)	C1C-B6C-B2C	60.0(8)
B11-C10-B9	114.2(7)	B2C-B6C-C10C	104.0(9)
C14-C13-B7	115.7(8)	B2C-B6C-B11C	60.5(8)
C13-C14-C15	113.4(7)	C1C-B6C-B5C	57.6(8)
O16-C15-C14	110.8(7)	C10C-B6C-B5C	54.8(8)
C18-C17-B8	120.3(15)	C13C-B7C-B11C	123.3(8)
C17-C18-C19	115.1(15)	C13C-B7C-B2C	124.2(9)
O20-C19-C18	123.9(13)	B11C-B7C-B2C	59.6(7)
C1-B2-B6	60.1(8)	B12C-B7C-B8C	59.2(7)
B6-B2-B3	110.3(10)	B2C-B7C-B8C	107.9(8)
B6-B2-B7	59.8(7)	B12C-B7C-B3C	105.5(8)
C1-B2-B8	105.5(8)	B2C-B7C-B3C	61.1(7)
B3-B2-B8	59.1(9)	C17C-B8C-B3C	125.4(11)
C1-B3-B9	104.1(10)	B3C-B8C-B12C	107.4(8)
C1-B3-B8	106.7(8)	B3C-B8C-B4C	60.8(7)
B9-B3-B8	60.9(8)	C17C-B8C-B7C	121.2(10)
B4-B3-B2	108.4(12)	B12C-B8C-B7C	59.2(7)
B8-B3-B2	61.8(7)	C17C-B8C-B9C	120.3(11)
C1-B4-C10	98.7(10)	B12C-B8C-B9C	59.7(8)
C10-B4-B9	60.5(7)	B7C-B8C-B9C	107.3(8)
C10-B4-B3	105.6(10)	C10C-B9C-B4C	105.0(10)
C1-B4-B5	58.2(9)	C10C-B9C-B12C	59.3(7)
B9-B4-B5	106.8(10)	B4C-B9C-B12C	106.4(9)
C10-B5-C1	98.3(12)	B5C-B9C-B8C	107.6(10)
C10-B5-B6	104.6(10)	B12C-B9C-B8C	58.1(7)
C1-B5-B6	57.7(8)	C10C-B11C-B2C	102.8(8)
B11-B5-B4	107.0(10)	B2C-B11C-B7C	60.7(7)
B6-B5-B4	104.3(11)	B2C-B11C-B12C	106.9(8)
C1-B6-B2	58.5(8)	C10C-B11C-B6C	60.0(8)
B2-B6-B7	61.5(7)	B7C-B11C-B6C	107.8(8)
B2-B6-B11	108.6(9)	C10C-B12C-B8C	104.7(8)
C1-B6-B5	59.4(9)	C10C-B12C-B11C	60.8(8)
B7-B6-B5	108.4(9)	B8C-B12C-B11C	110.1(9)
C13-B7-B11	123.1(9)	B7C-B12C-B9C	111.2(8)
C13-B7-B2	122.9(8)	B11C-B12C-B9C	109.2(1)
B11-B7-B2	106.2(8)	B4B-C1B-B5B	62.9(8)
B6-B7-B12	106.6(8)	B5B-C1B-B6B	64.4(8)
B2-B7-B12	104.9(7)	B5B-C1B-B2B	115.5(8)
B6-B7-B8	107.2(9)	B4B-C1B-B3B	64.2(7)
B2-B7-B8	59.3(7)	B6B-C1B-B3B	114.1(8)
C17-B8-B3	128.6(13)	B9B-C10B-B12B	63.8(7)
B3-B8-B9	59.3(8)	B9B-C10B-B11B	116.8(8)

B3-B8-B2	59.1(8)	B12B-C10B-B11B	63.4(6)
C17-B8-B12	119.7(12)	B5B-C10B-B6B	62.5(7)
B9-B8-B12	58.7(6)	B11B-C10B-B6B	61.2(7)
C17-B8-B7	117.6(12)	C14B-C13B-B7B	114.0(7)
B9-B8-B7	106.0(8)	C15B-C14B-C13B	111.4(7)
B12-B8-B7	58.8(6)	O16B-C15B-C14B	111.9(7)
C10-B9-B3	103.8(11)	C18B-C17B-B8B	115.6(9)
C10-B9-B12	58.7(6)	C17B-C18B-C19B	114.7(10)
B3-B9-B12	107.9(8)	O20B-C19B-C18B	112.3(10)
B4-B9-B8	109.5(10)	C1B-B2B-B6B	59.4(8)
B12-B9-B8	61.7(7)	B6B-B2B-B11B	61.3(7)
C10-B11-B5	57.4(9)	B6B-B2B-B3B	109.9(10)
B5-B11-B6	61.7(8)	C1B-B2B-B7B	105.4(8)
B5-B11-B12	107.8(10)	B11B-B2B-B7B	60.7(6)
C10-B11-B7	105.5(7)	C1B-B3B-B7B	105.7(7)
B6-B11-B7	59.1(7)	C1B-B3B-B8B	106.4(8)
C10-B12-B11	58.8(7)	B7B-B3B-B8B	59.8(5)
C10-B12-B7	104.7(7)	B2B-B3B-B4B	105.7(9)
B11-B12-B7	59.6(6)	B8B-B3B-B4B	63.2(6)
B9-B12-B8	59.7(7)	C1B-B4B-B5B	58.7(8)
B7-B12-B8	62.0(6)	B5B-B4B-B9B	61.6(8)
B6C-C1C-B2C	60.7(8)	B5B-B4B-B3B	108.6(9)
B2C-C1C-B5C	114.9(9)	C1B-B4B-B8B	104.2(8)
B2C-C1C-B3C	64.0(7)	B9B-B4B-B8B	60.3(6)
B6C-C1C-B4C	116.2(9)	C1B-B5B-B4B	58.4(8)
B5C-C1C-B4C	61.6(8)	C1B-B5B-B6B	58.0(7)
B9C-C10C-B12C	64.6(8)	B4B-B5B-B6B	107.4(9)
B9C-C10C-B11C	117.3(9)	C10B-B5B-B9B	55.9(7)
B12C-C10C-B11C	61.9(7)	B6B-B5B-B9B	106.6(8)
B5C-C10C-B6C	65.1(9)	C1B-B6B-B2B	59.5(7)
B11C-C10C-B6C	60.6(8)	B2B-B6B-C10B	104.0(9)
C14C-C13C-B7C	115.0(8)	B2B-B6B-B11B	60.9(7)
C15C-C14C-C13C	114.1(8)	C1B-B6B-B5B	57.6(8)
O16C-C15C-C14C	115.8(12)	C10B-B6B-B5B	57.6(8)
C1C-B2C-B6C	59.3(8)	C13B-B7B-B8B	123.9(9)
B6C-B2C-B11C	61.3(8)	C13B-B7B-B3B	121.3(8)
B6C-B2C-B7C	109.5(10)	B8B-B7B-B3B	60.2(5)
C1C-B2C-B3C	58.1(7)	B12B-B7B-B11B	61.0(6)
B11C-B2C-B3C	107.2(9)	B3B-B7B-B11B	106.2(7)
C1C-B3C-B4C	59.7(7)	B12B-B7B-B2B	106.2(7)
C1C-B3C-B7C	104.1(8)	B3B-B7B-B2B	59.1(6)
B4C-B3C-B7C	109.0(8)	C17B-B8B-B12B	125.9(8)
B8C-B3C-B2C	108.4(8)	B12B-B8B-B7B	59.9(5)
B7C-B3C-B2C	59.2(6)	B12B-B8B-B3B	106.5(7)
C1C-B4C-B9C	103.5(9)	C17B-B8B-B9B	120.4(9)
B9C-B4C-B5C	59.2(8)		

Characterization of 9,10-$(ClCH_2CH_2CH_2)_2$-1,7-closo-$C_2B_{10}H_{10}$, 5, in d6-acetone.

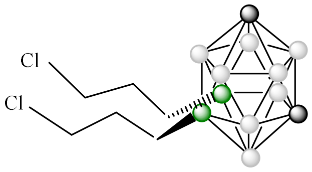

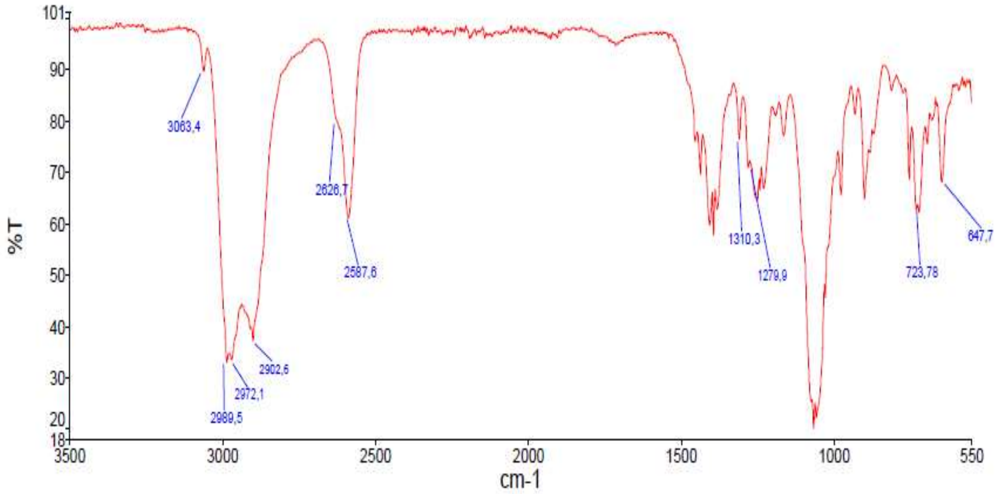

Figure S31. IR-ATR spectrum.

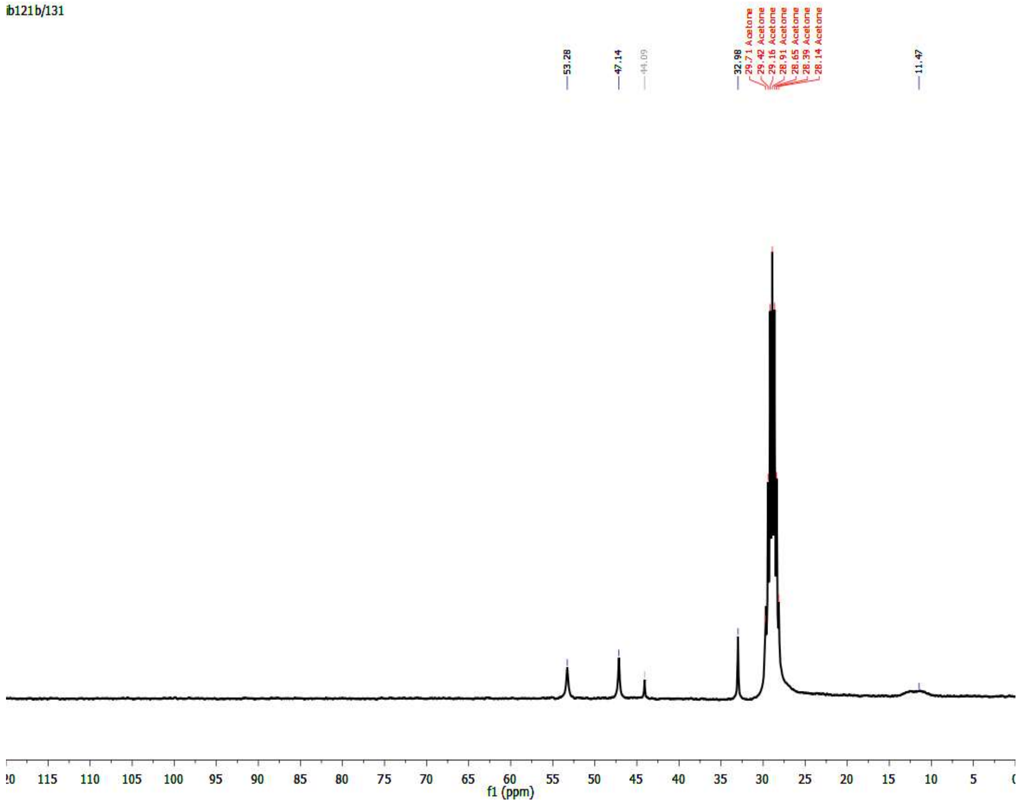

Figure S32. ${}^{13}C\{{}^{1}H\}$-NMR spectrum.

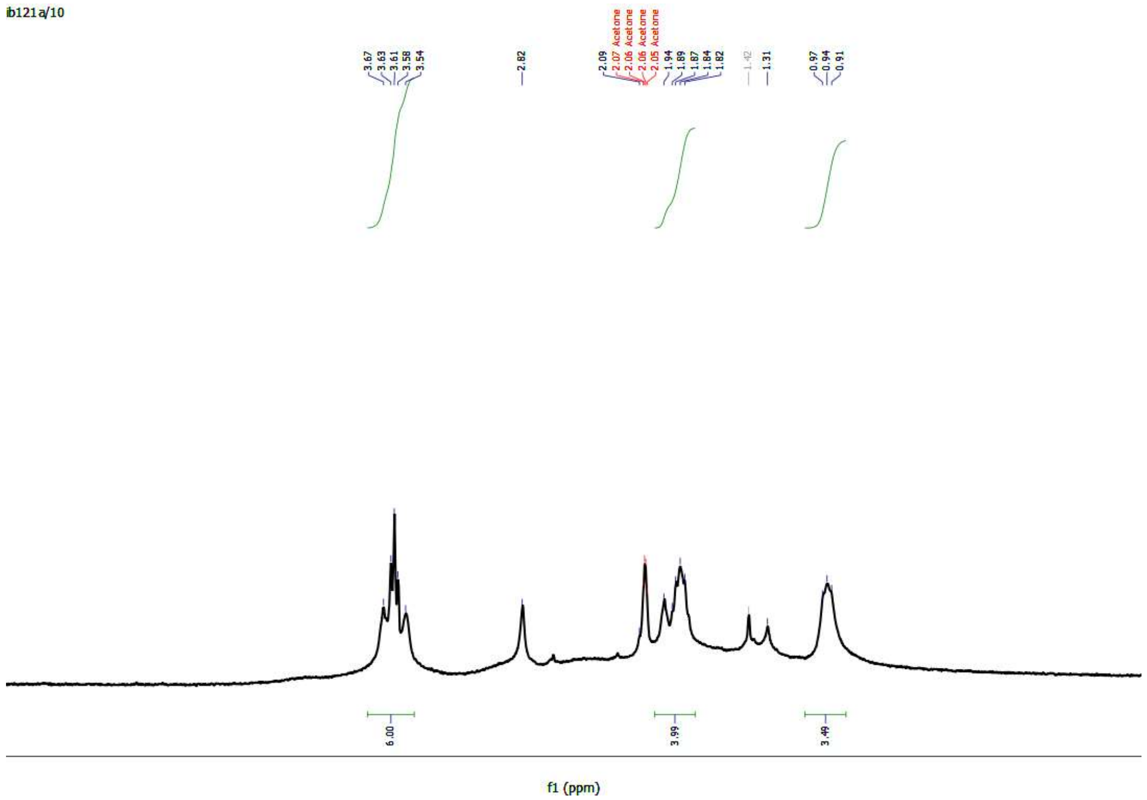

Figure S33. ^{1}H-NMR spectrum ($(CD_3)_2CO$)

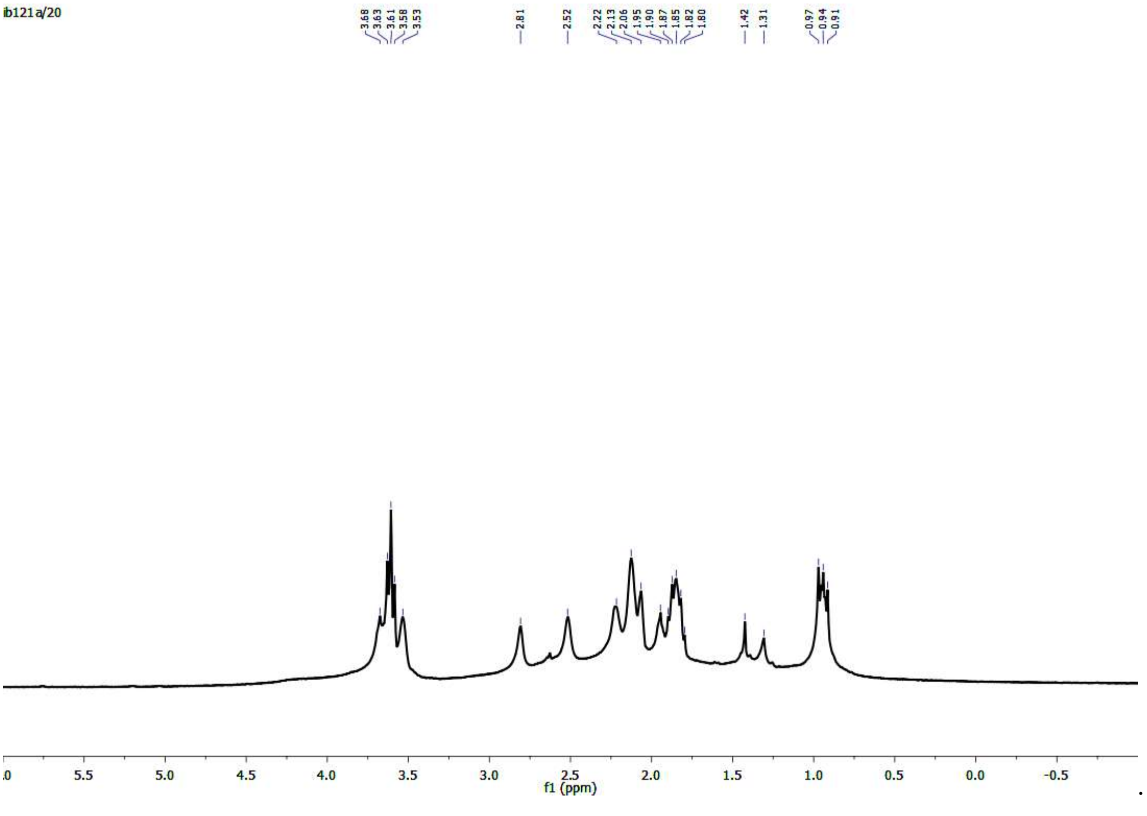

Figure S34. $^{1}H\{^{11}B\}$-NMR spectrum ($(CD_3)_2CO$).

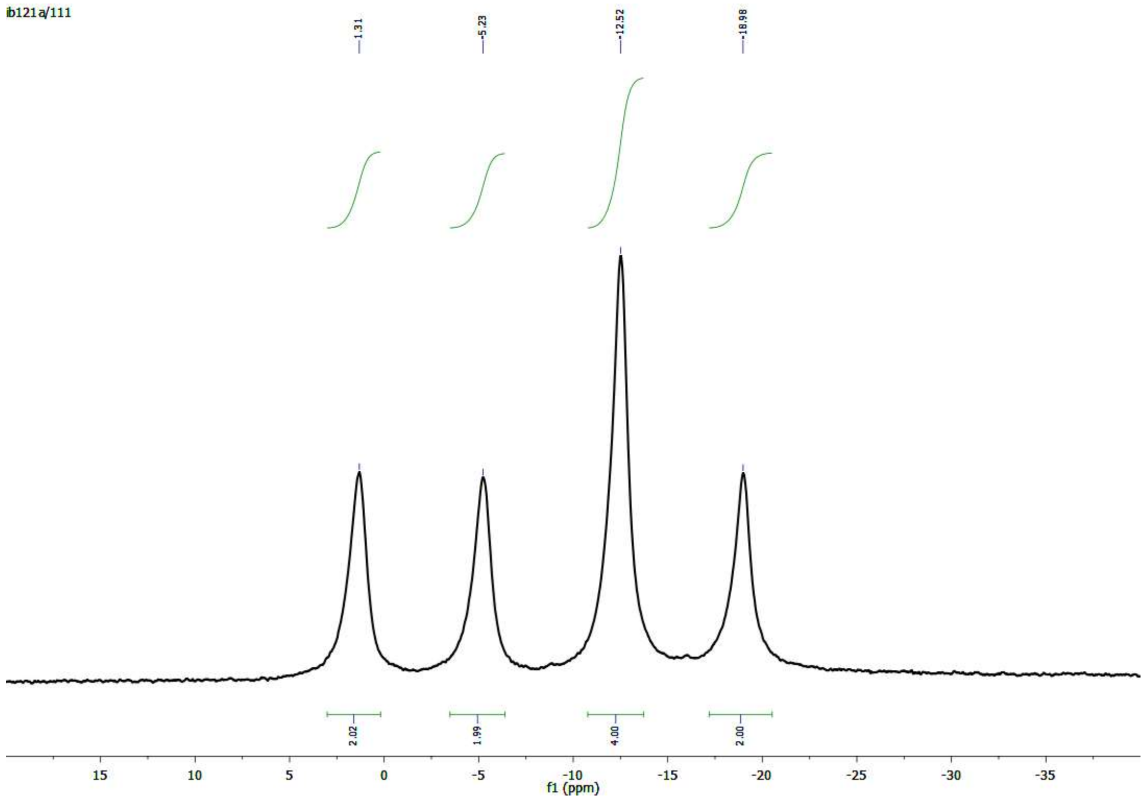

Figure S35. $^{11}B\{^{1}H\}$-NMR spectrum ($(CD_3)_2CO$).

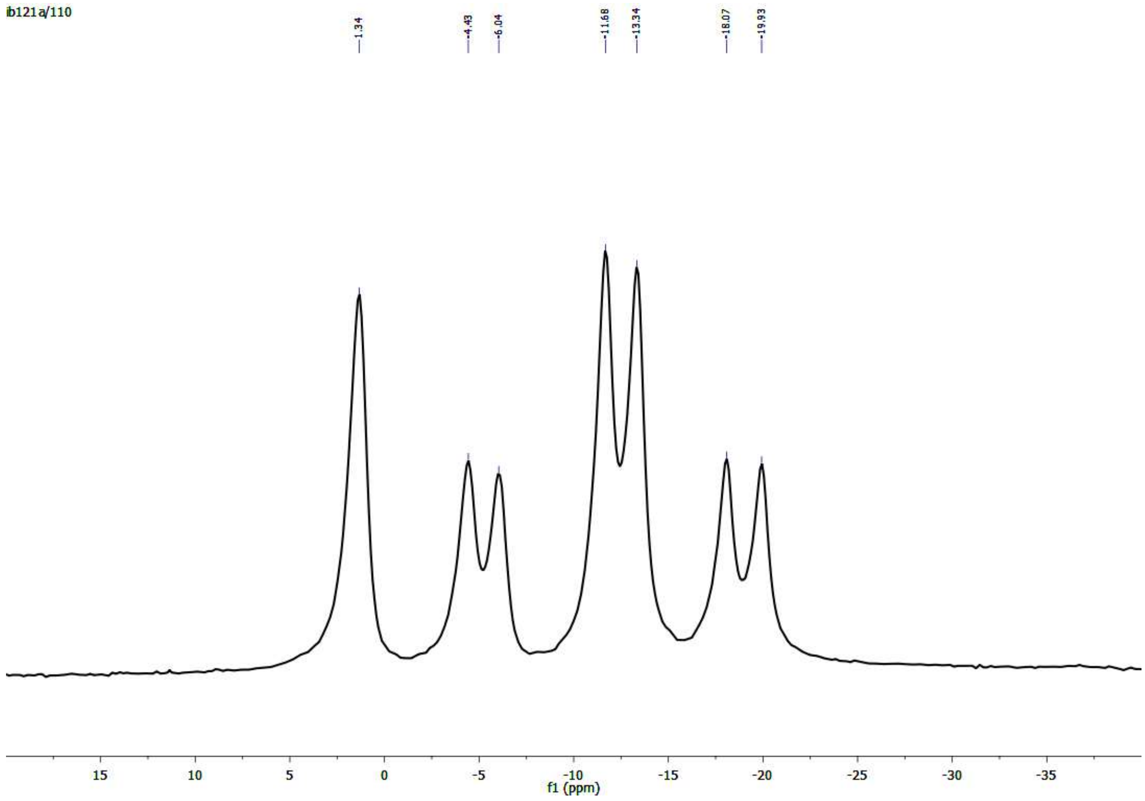

Figure S36. ^{11}B-NMR spectrum ($(CD_3)_2CO$).

Characterization of 9,10-($C_6H_5COOCH_2CH_2CH_2$)$_2$-1,7-*closo*-$C_2B_{10}H_{10}$, 6, in d6-acetone.

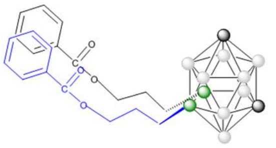

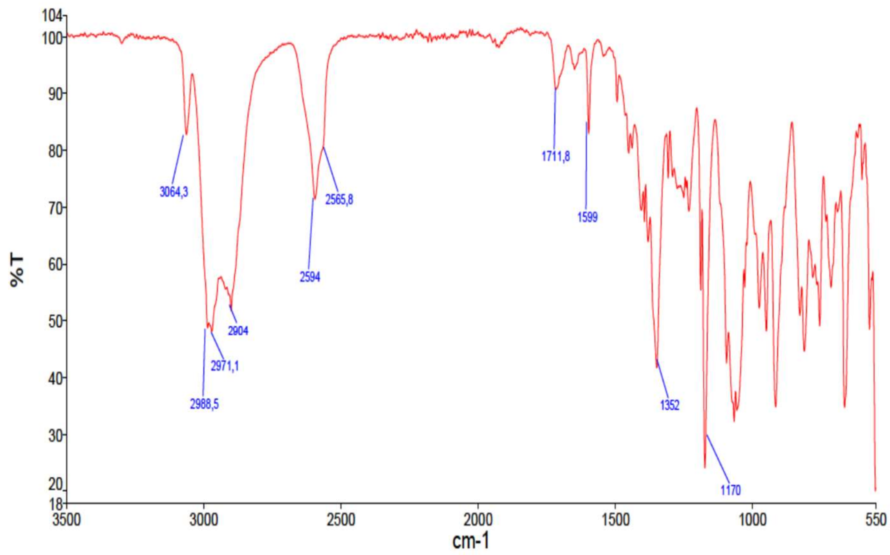

Figure S37. IR-ATR spectrum.

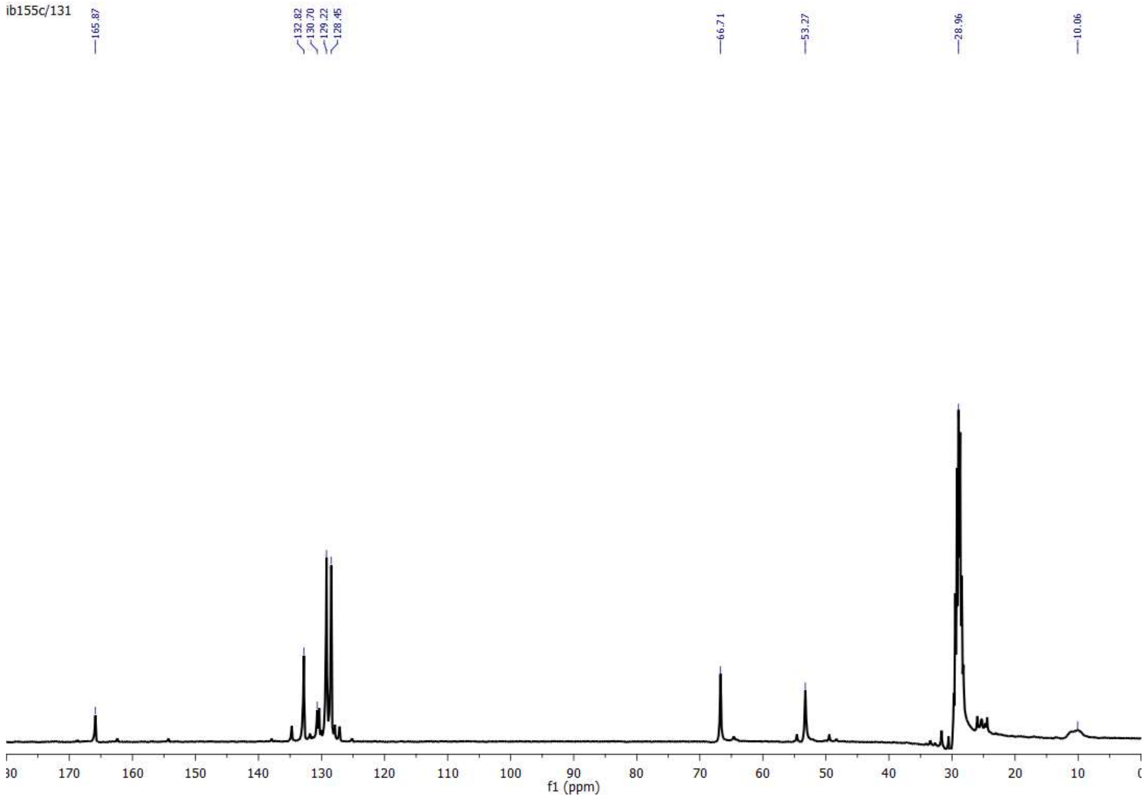

Figure S38. $^{13}C\{^1H\}$-NMR spectrum.

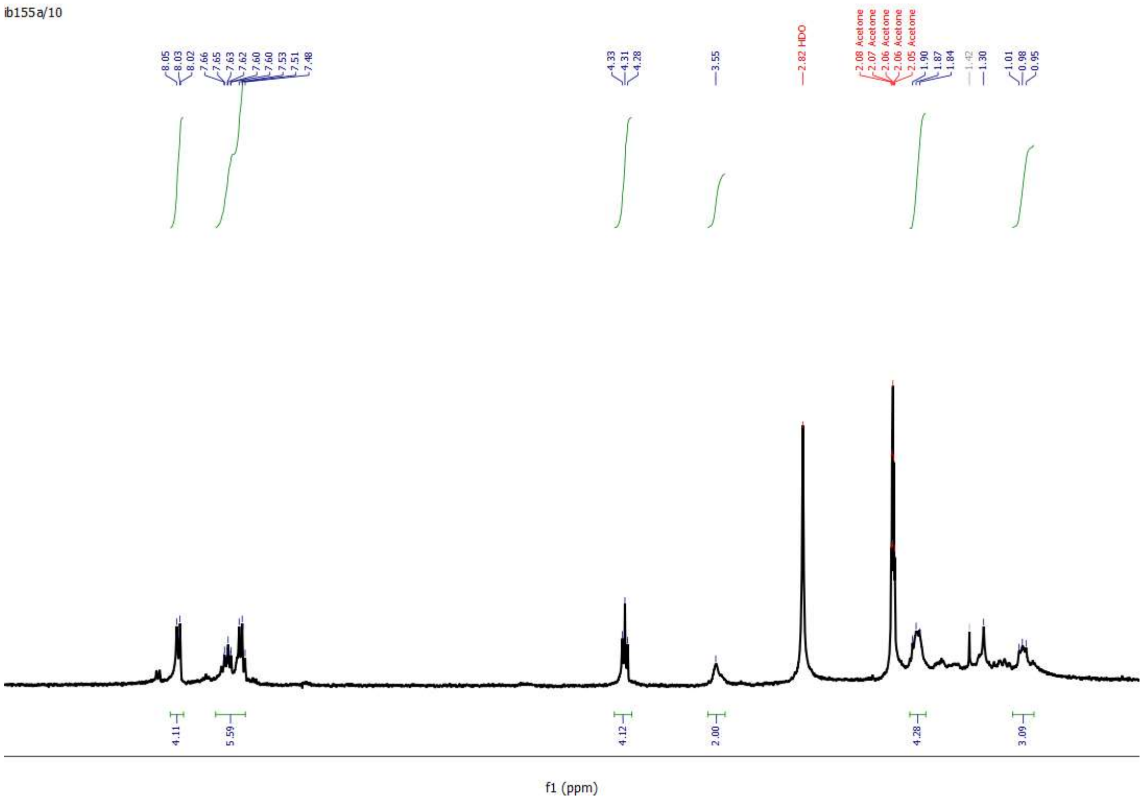

Figure S39. ^{1}H-NMR spectrum.

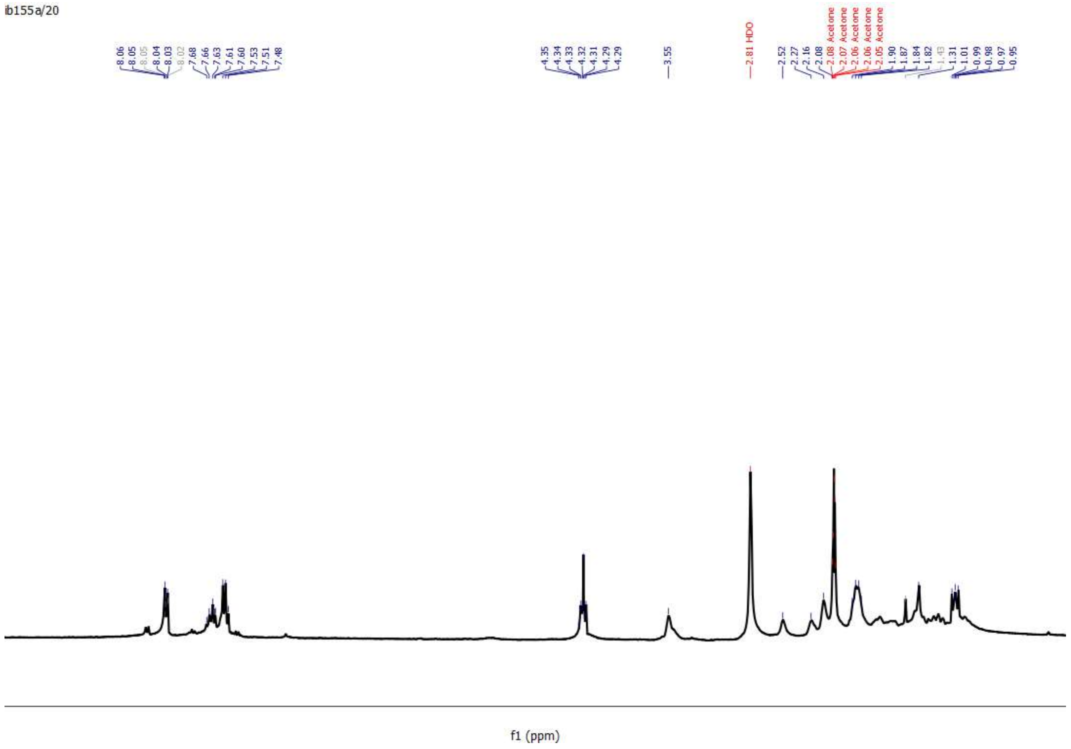

Figure S40. $^{1}H\{^{11}B\}$-NMR spectrum.

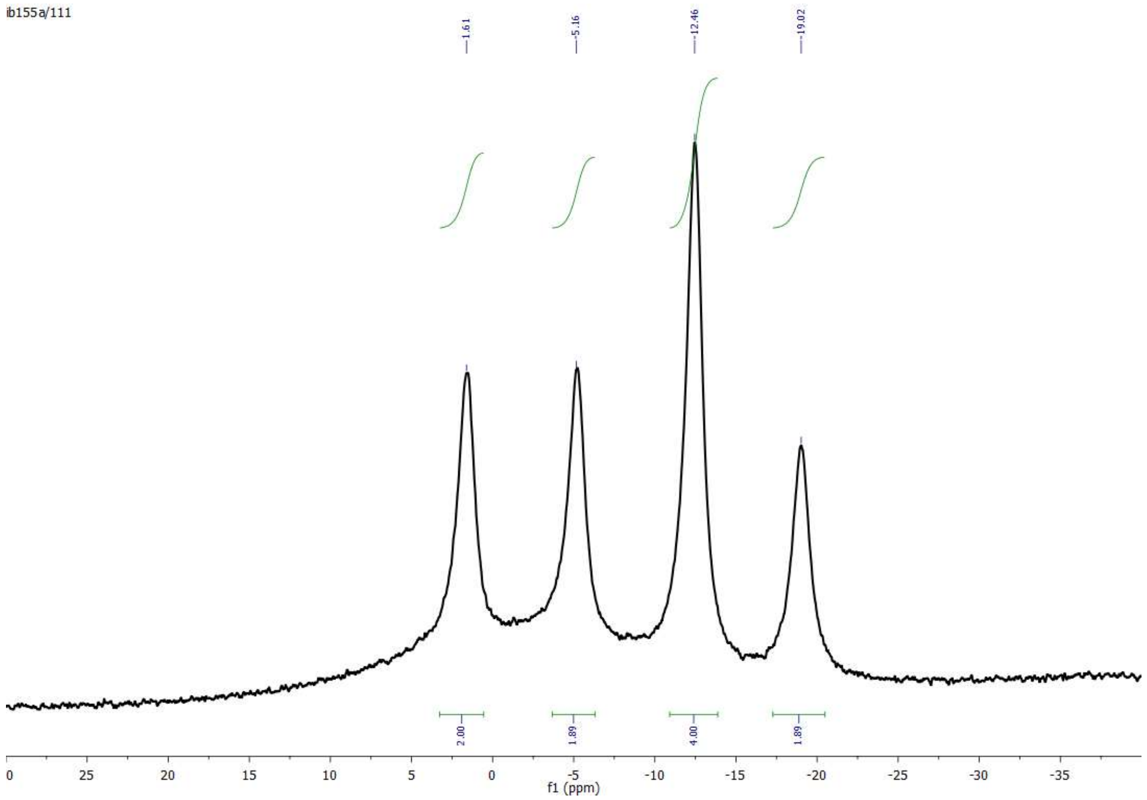

Figure S41. $^{11}B\{^{1}H\}$-NMR spectrum ($(CD_3)_2CO$).

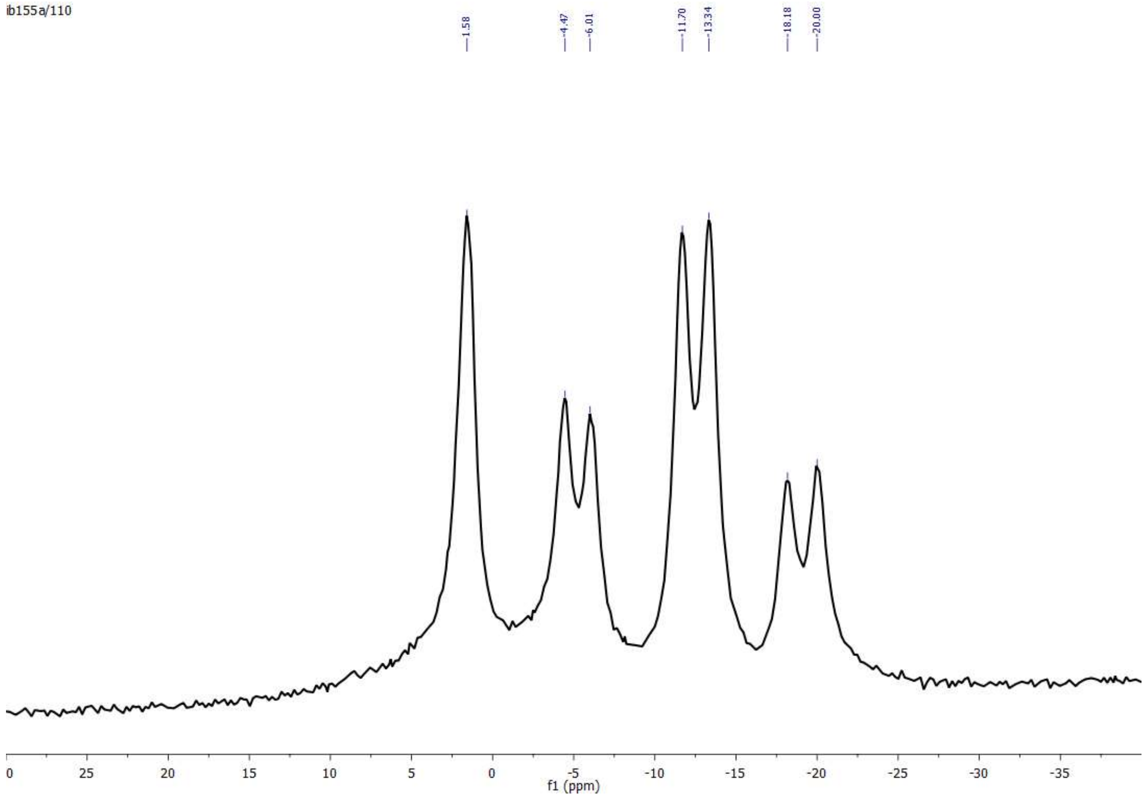

Figure S42. ^{11}B-NMR spectrum ($(CD_3)_2CO$).

Characterization of 9,10-(CH_3-C_6H_4-$SO_3(CH_2)_3)_2$-1,7-$C_2B_{10}H_{10}$, 7, in d6-acetone.

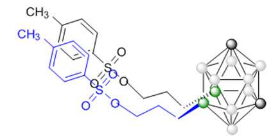

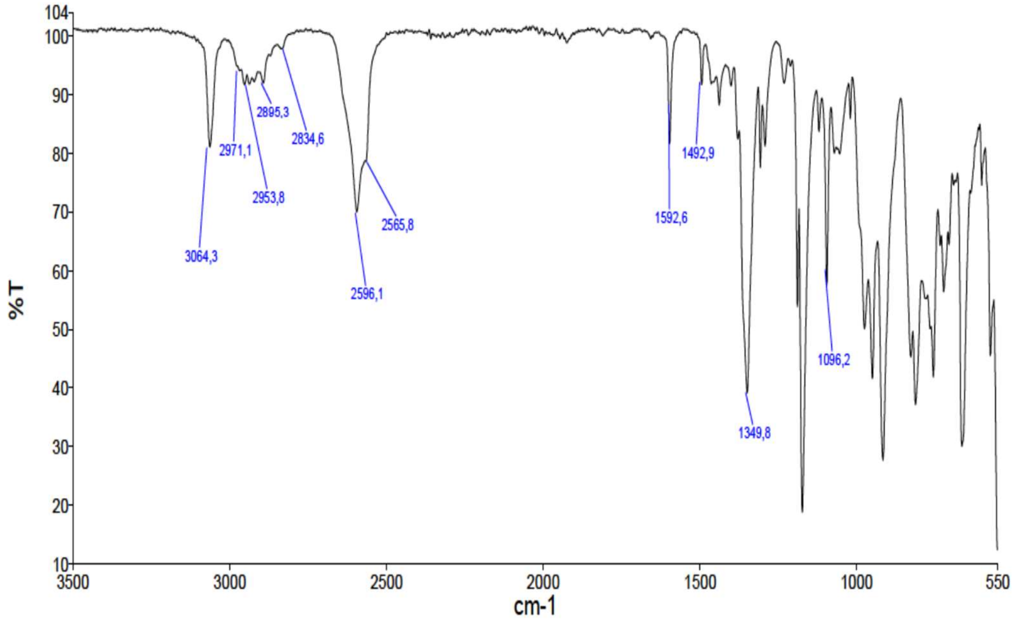

Figure S43. ATR spectrum.

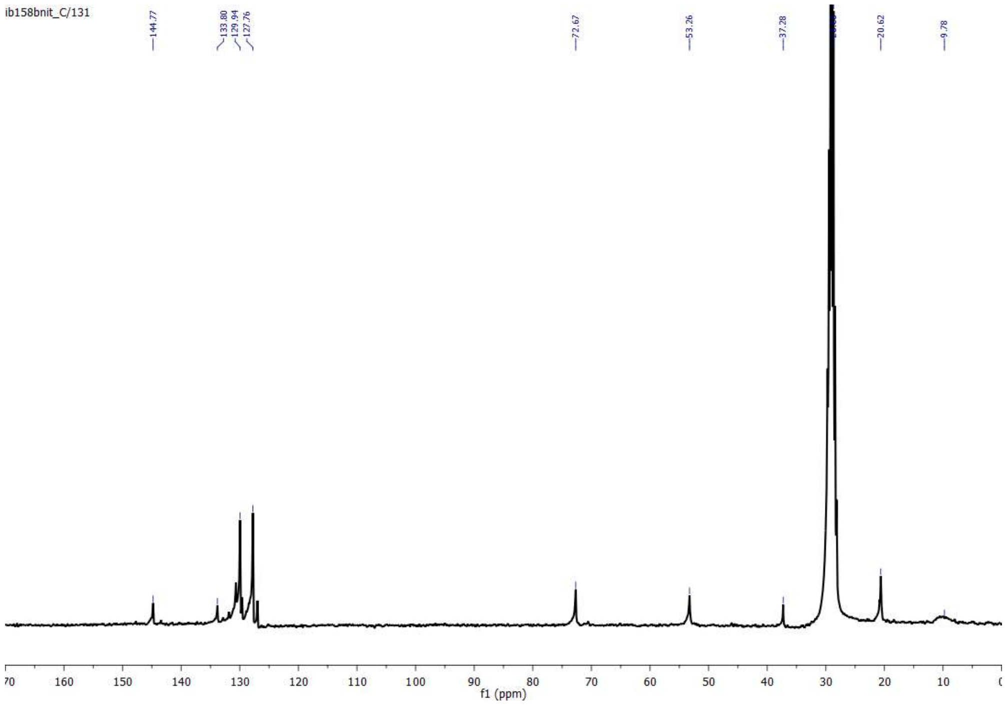

Figure S44. $^{13}C\{1H\}$-NMR spectrum.

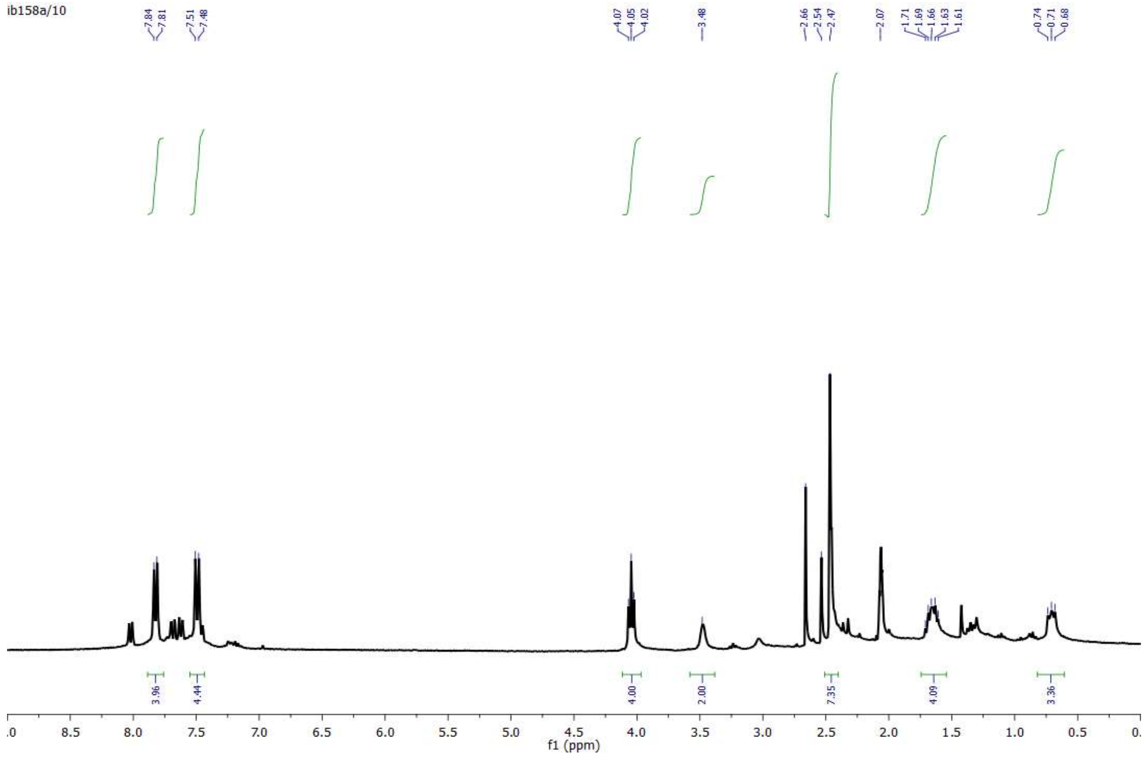

Figure S45. ^{1}H-NMR spectrum.

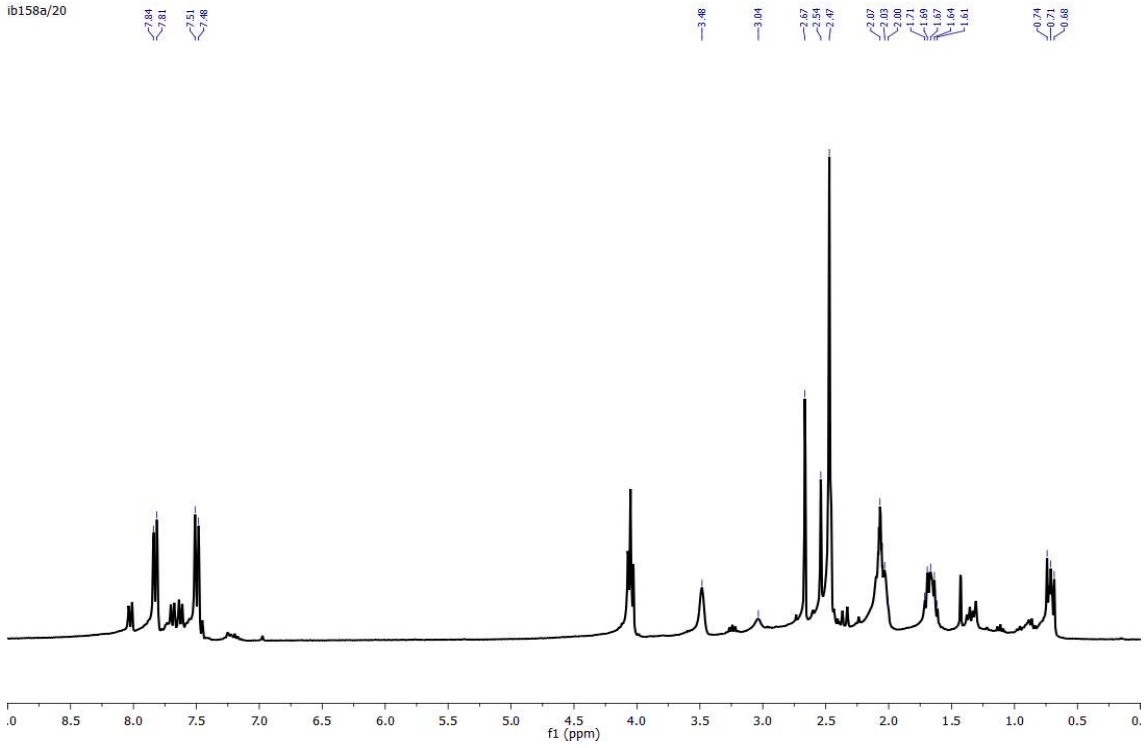

Figure S46. $^{1}H\{^{11}B\}$-NMR spectrum.

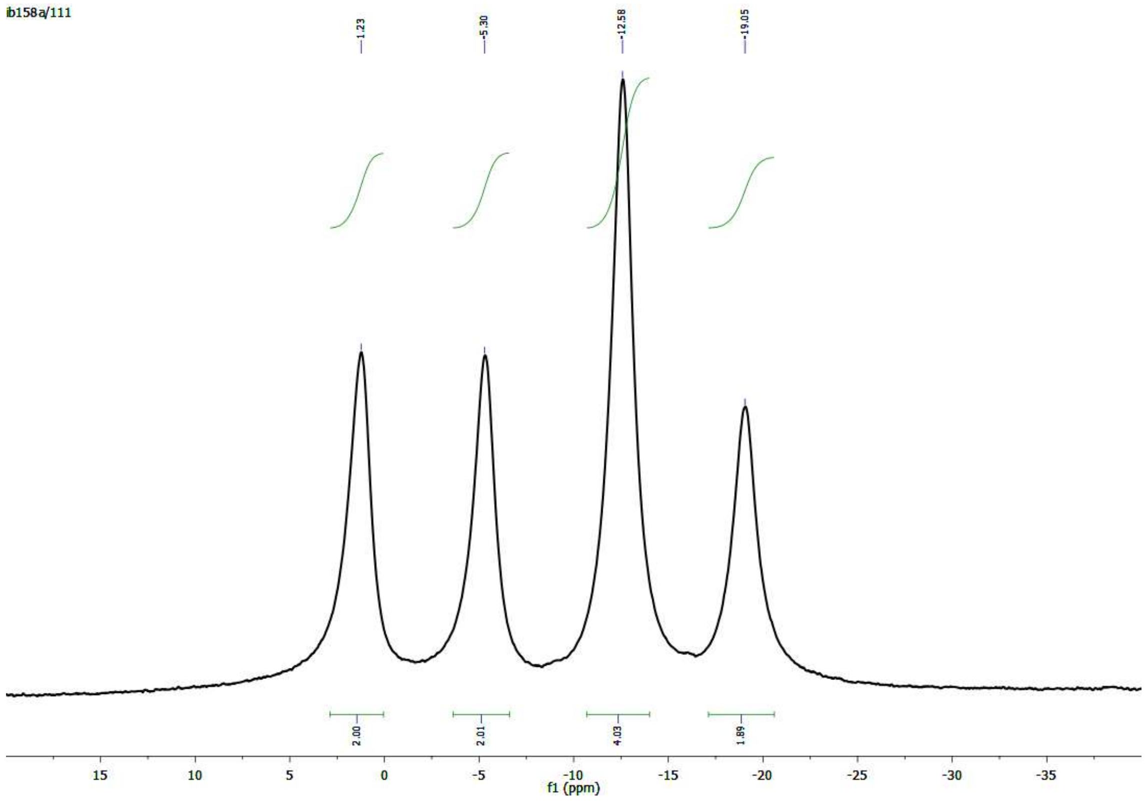

Figure S47. ^11^B{^1^H}-NMR spectrum ((CD$_3$)$_2$CO).

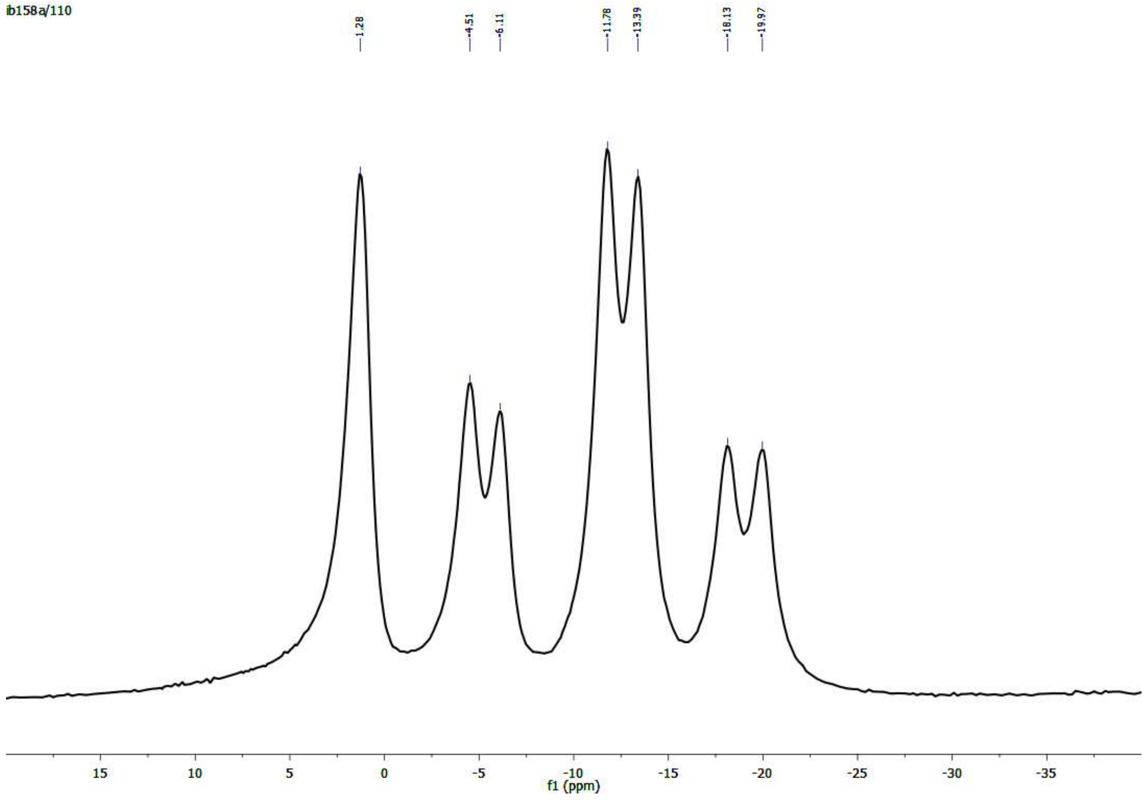

Figure S48. ^11^B-NMR spectrum ((CD$_3$)$_2$CO).

Characterization of 9,10-($N_3CH_2CH_2CH_2$)$_2$-1,7-*closo*-$C_2B_{10}H_{10}$, 8, in d6-acetone.

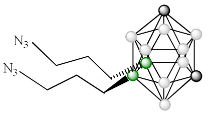

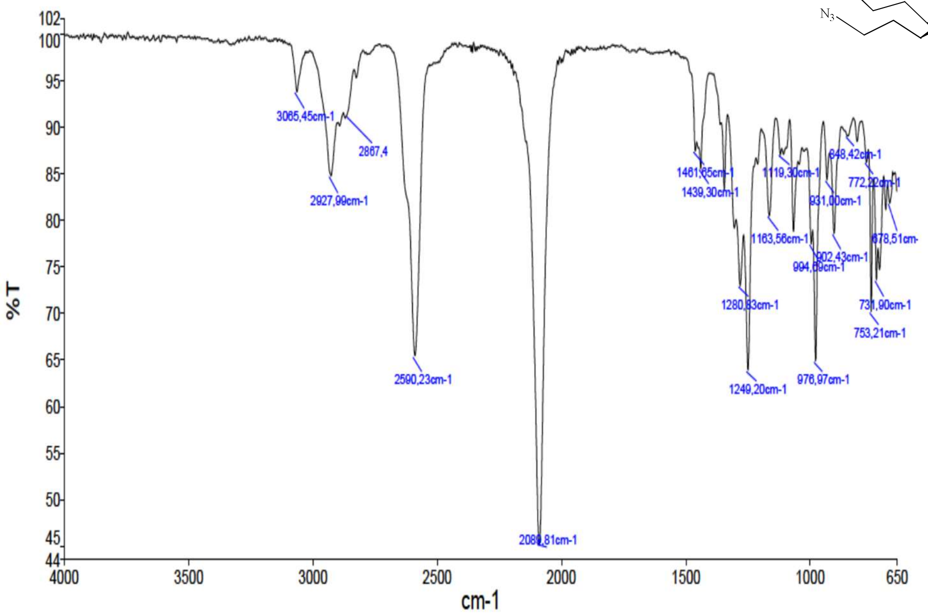

Figure S49. ATR spectrum.

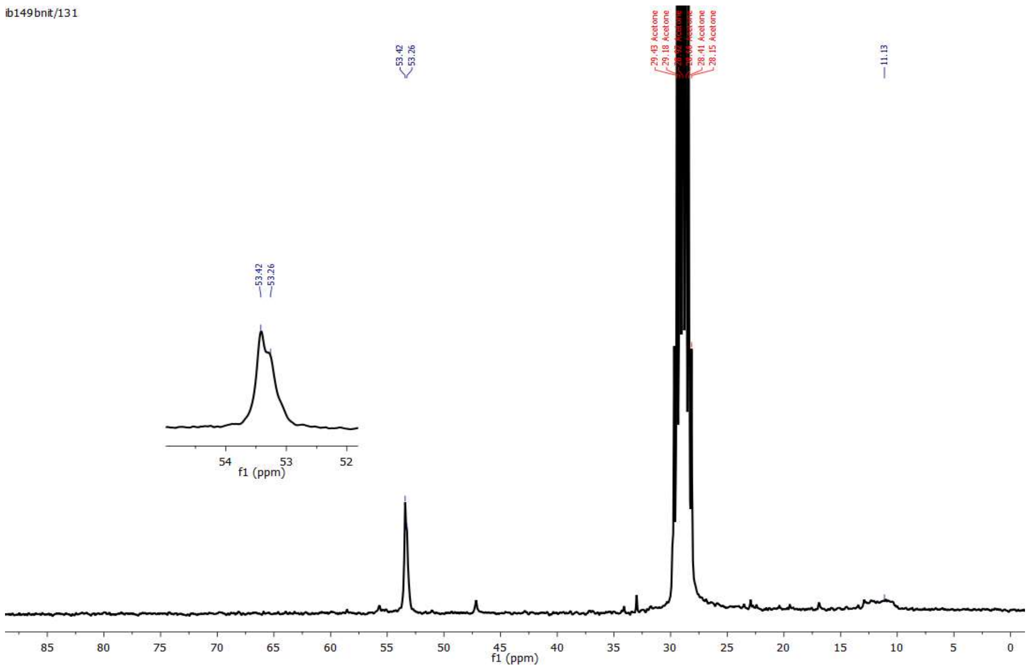

Figure S50. $^{13}C\{^1H\}$-NMR spectrum.

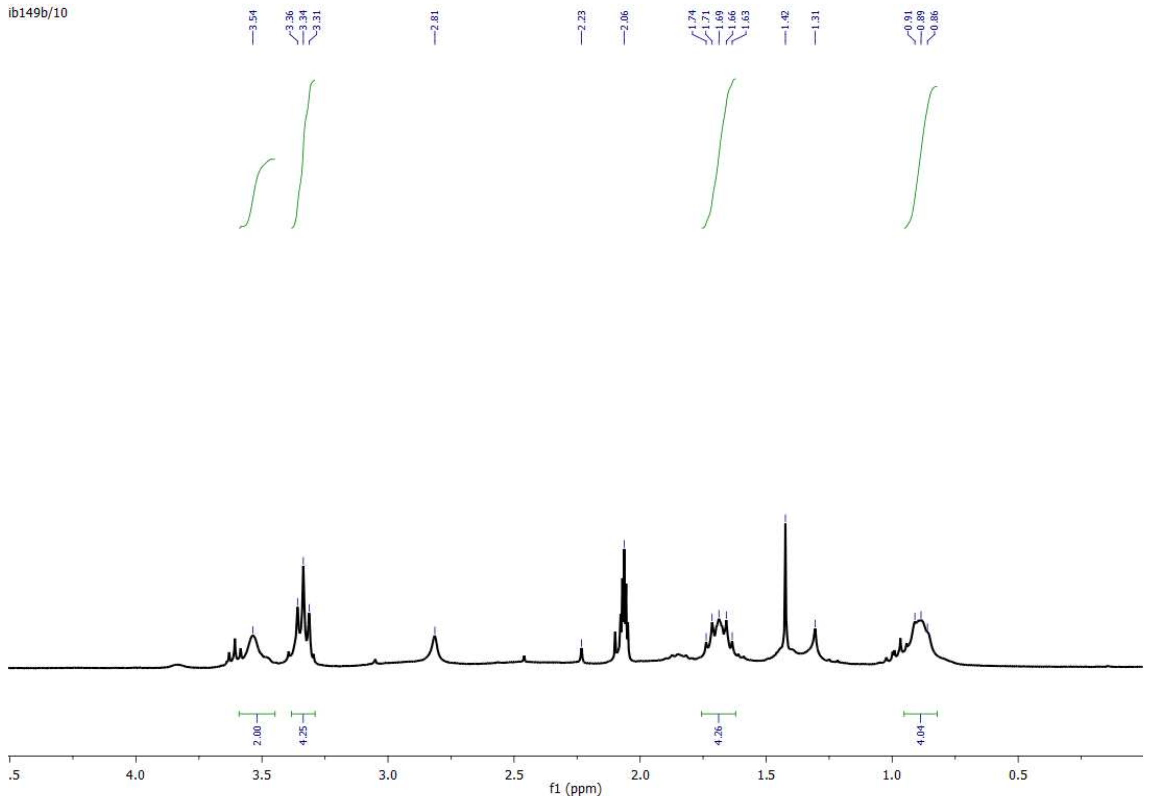

Figure S51. ^{1}H-NMR spectrum

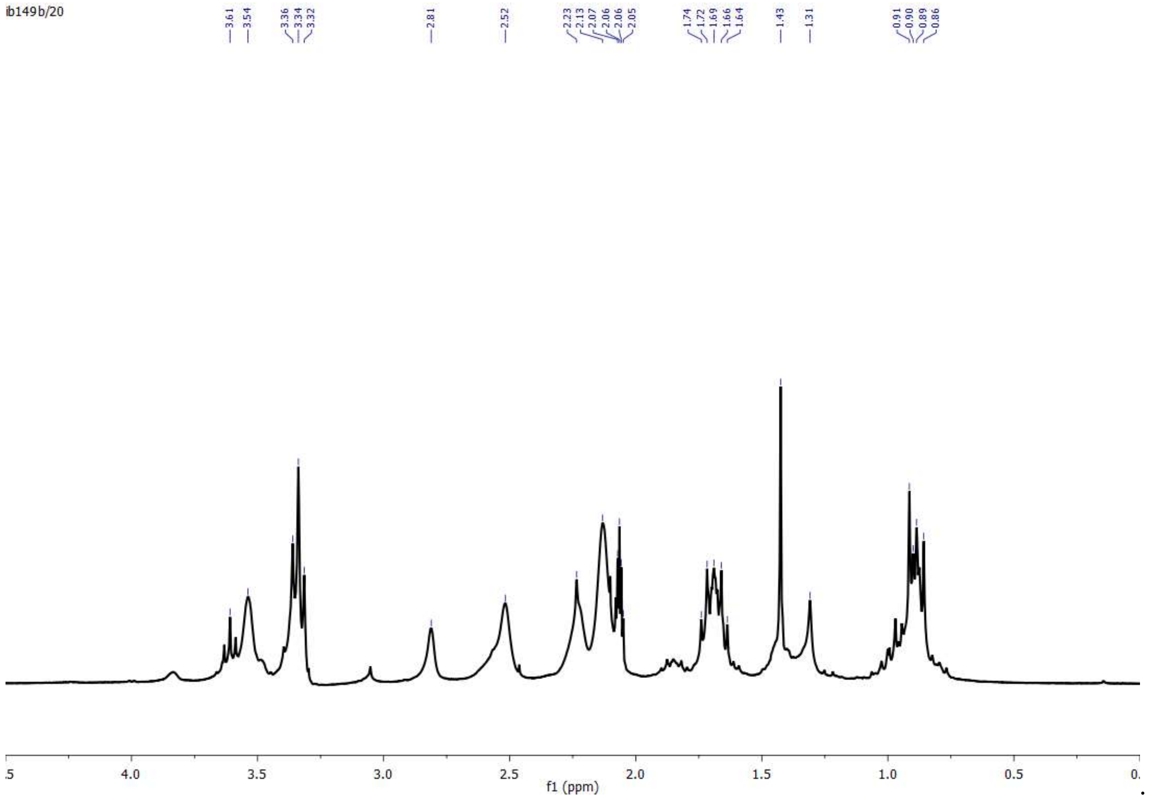

Figure S52. $^{1}H\{^{11}B\}$-NMR spectrum.

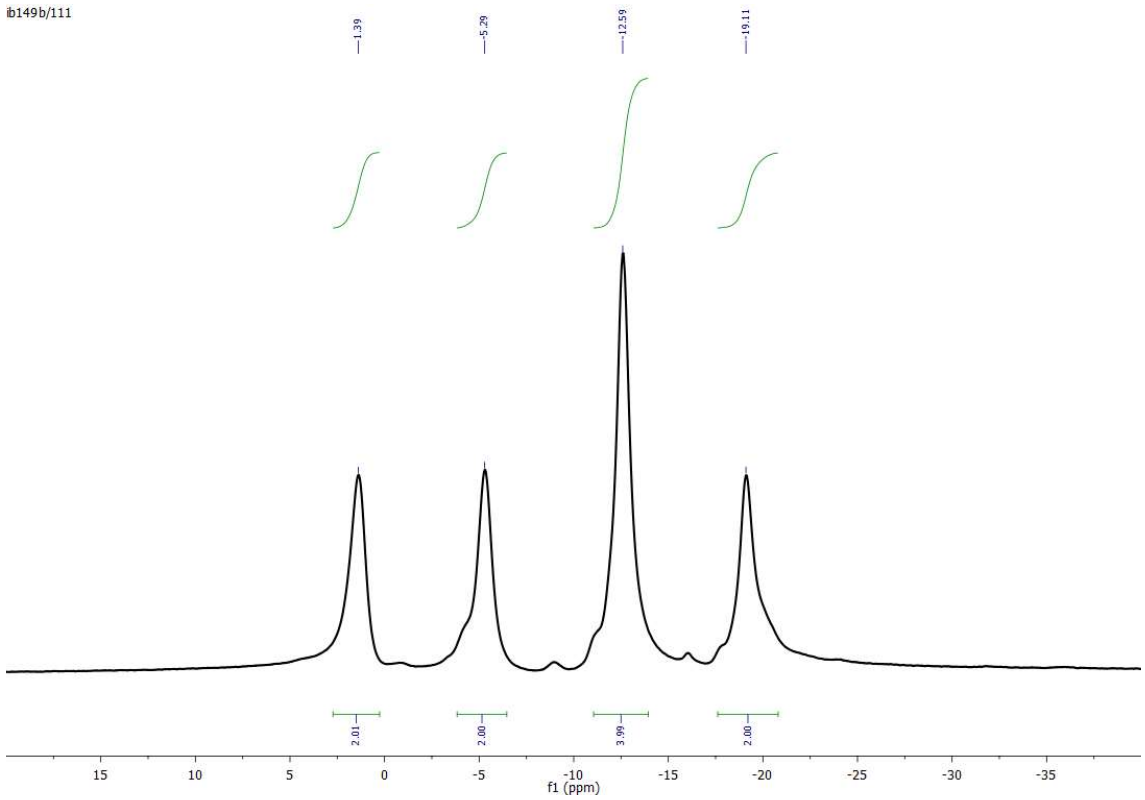

Figure S53. $^{11}B\{^{1}H\}$-NMR spectrum ($(CD_3)_2CO$).

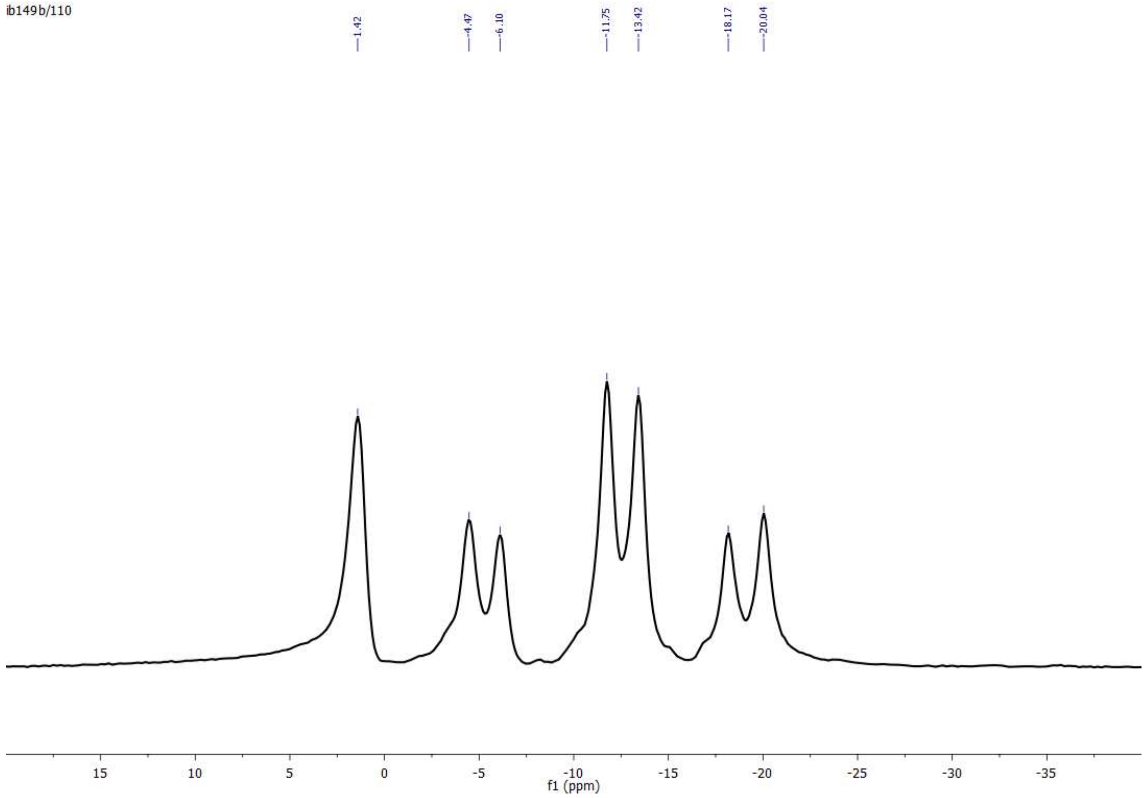

Figure S54. ^{11}B-NMR spectrum ($(CD_3)_2CO$).

Characterization of 9,10-($C_6H_5C_2N_3CH_2CH_2CH_2$)$_2$-1,7-*closo*-$C_2B_{10}H_{10}$, 9, in $(CD_3)_2SO$.

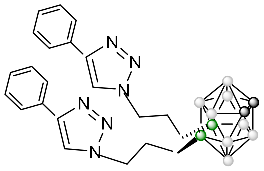

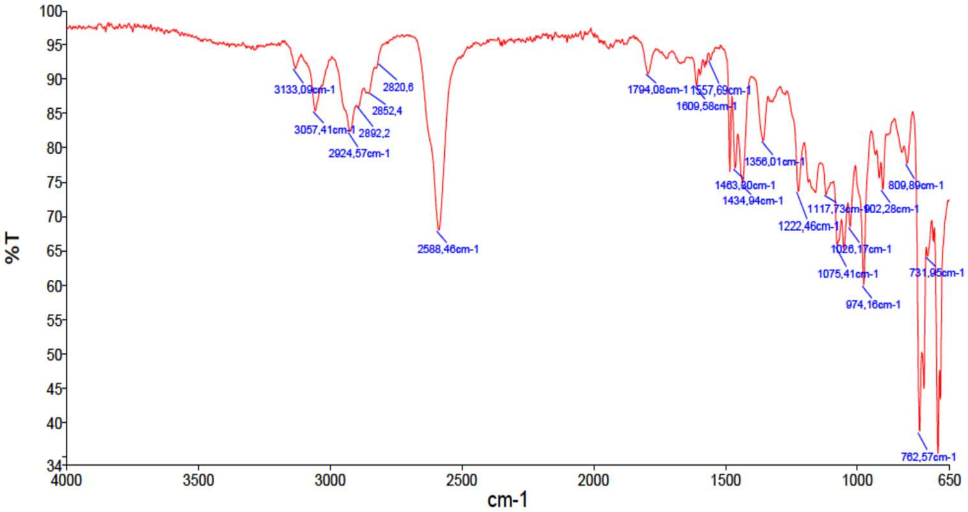

Figure S55. ATR-IR spectrum.

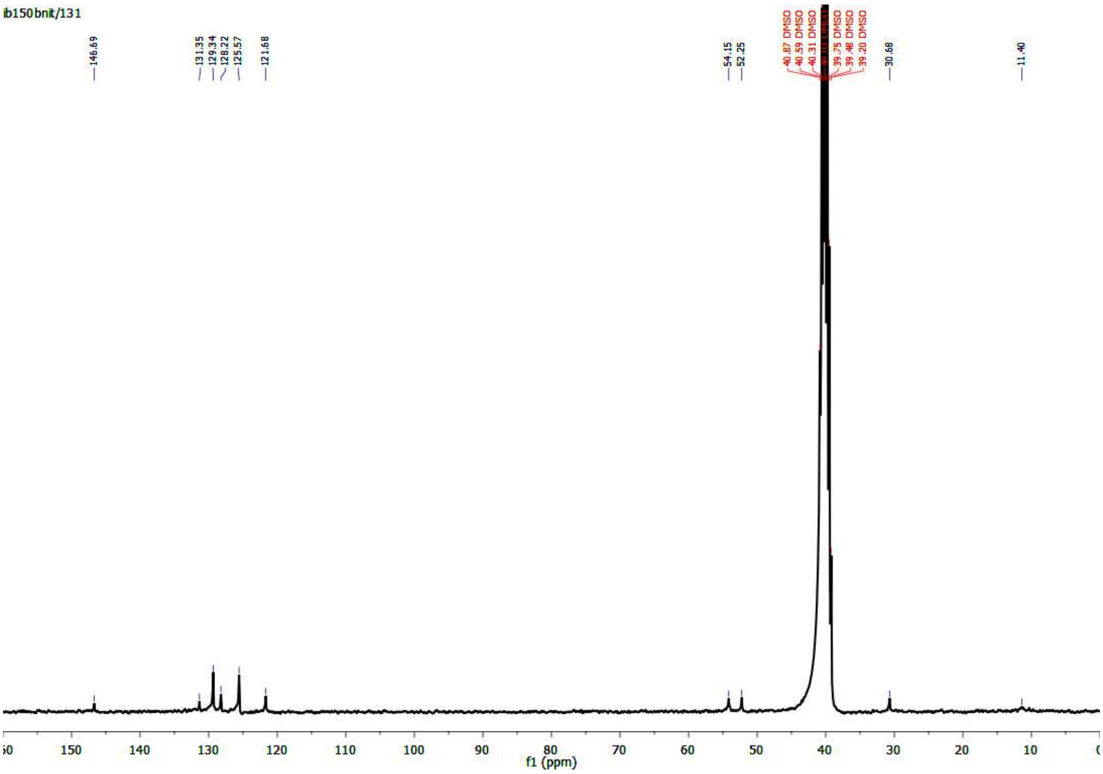

Figure S56. $^{13}C\{^1H\}$-NMR spectrum.

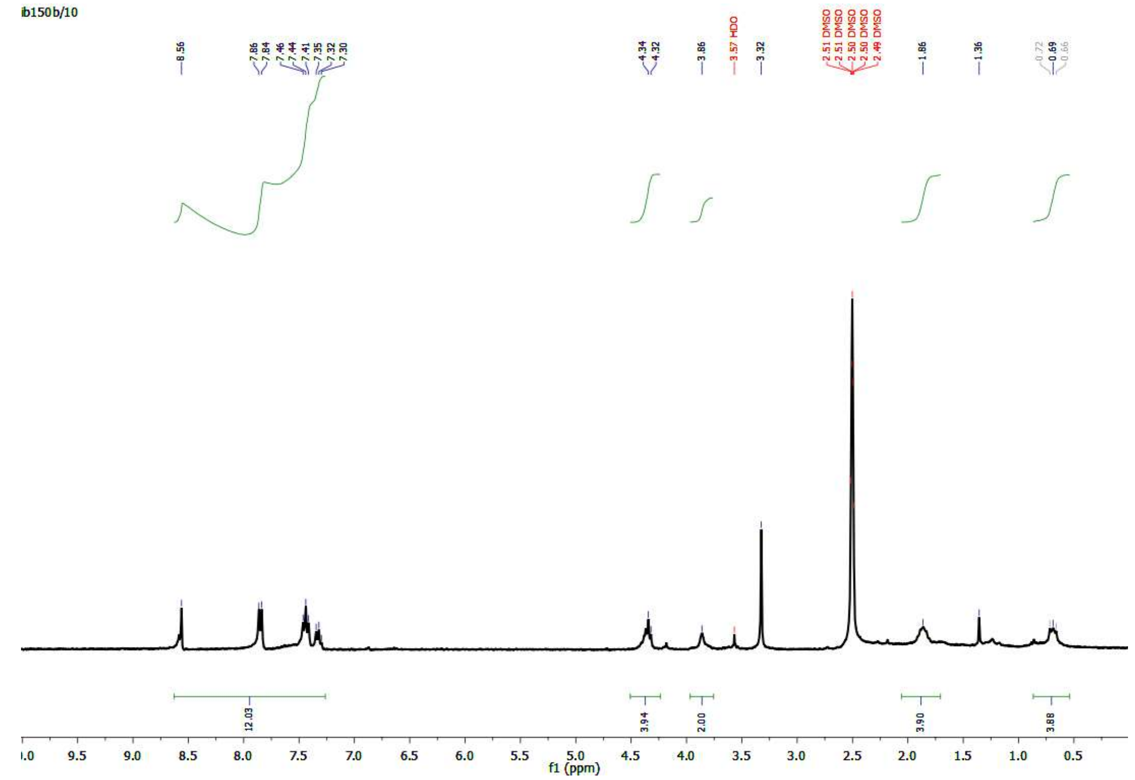

Figure S57. ^{1}H-NMR spectrum.

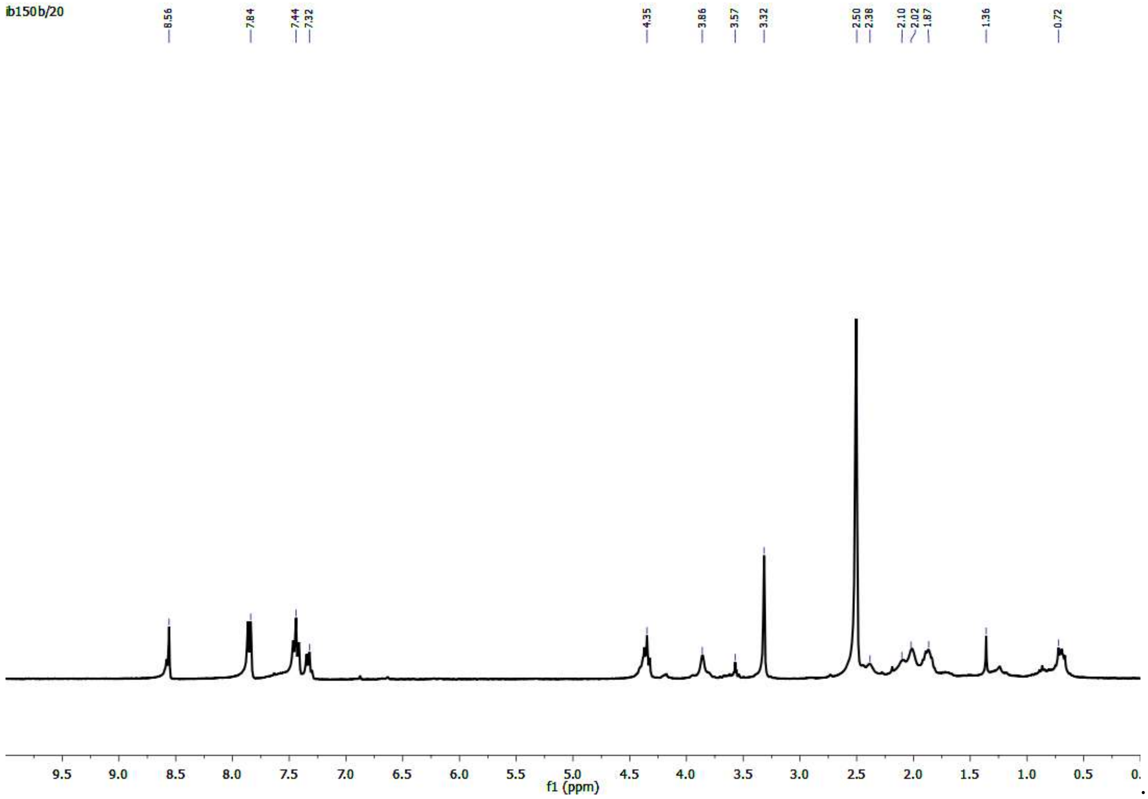

Figure S58. $^{1}H\{^{11}B\}$-NMR spectrum.

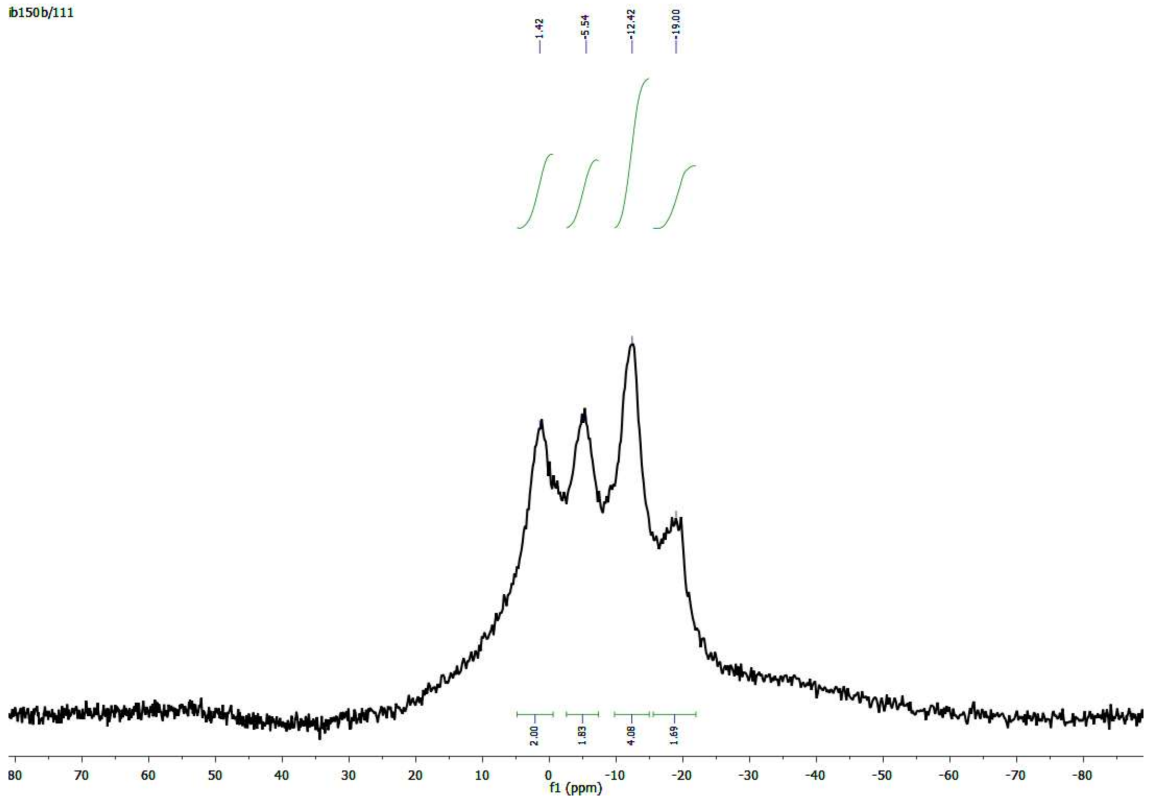

Figure S59. $^{11}B\{^{1}H\}$-NMR spectrum.

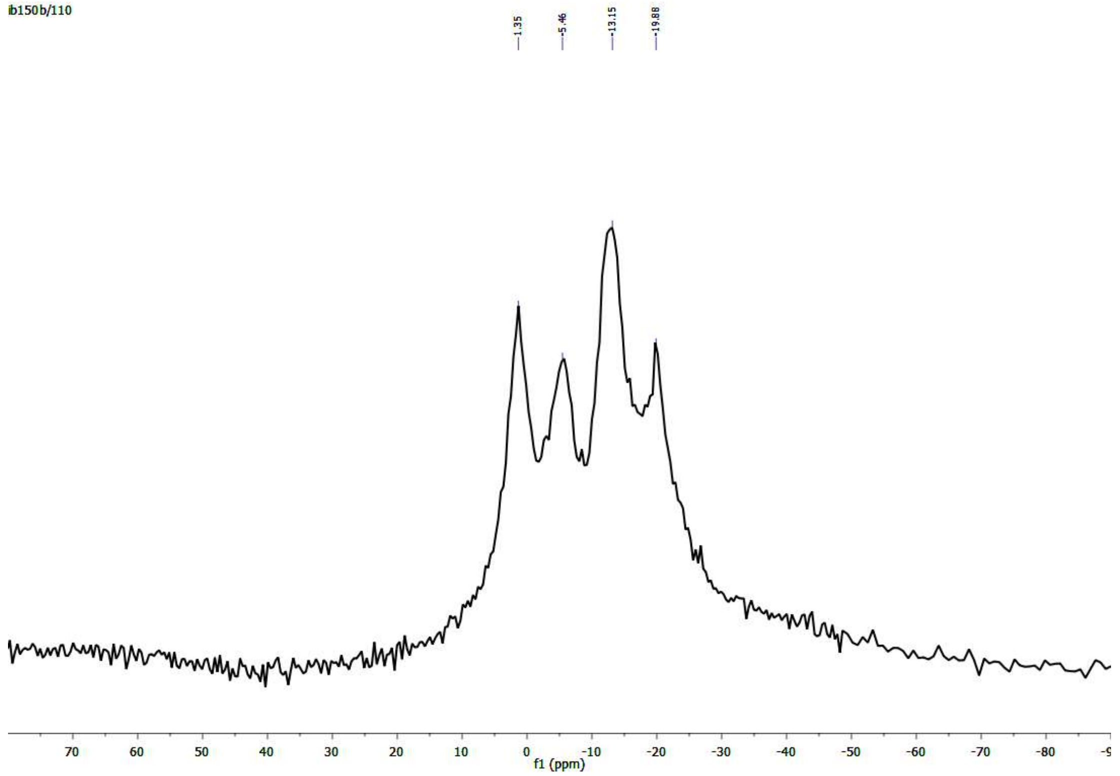

Figure S60. ^{11}B-NMR spectrum.

Characterization of 1,7-(CH_2=$CHCH_2$)$_2$-9,10-(CH_2=$CHCH_2$)$_2$-1,7-*closo*-$C_2B_{10}H_8$, 10, in d6-acetone.

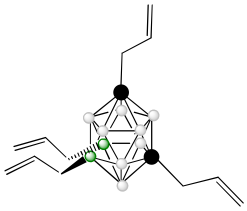

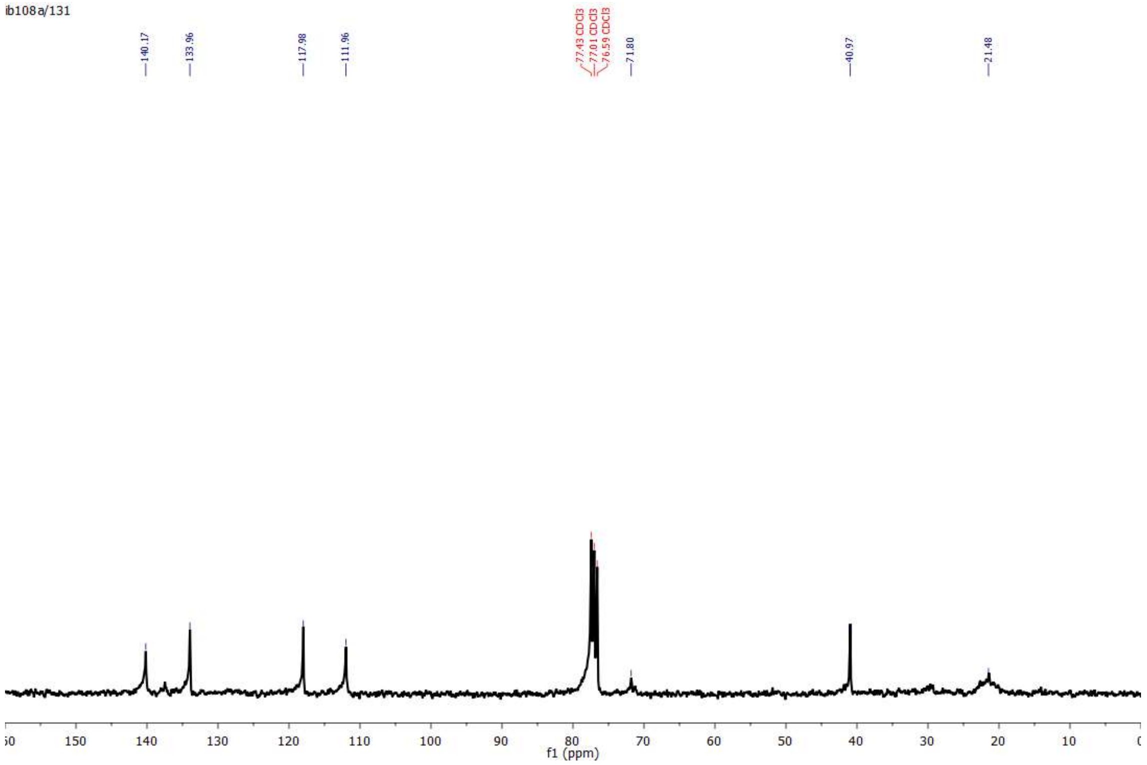

Figure S61. $^{13}C\{^1H\}$-NMR spectrum.

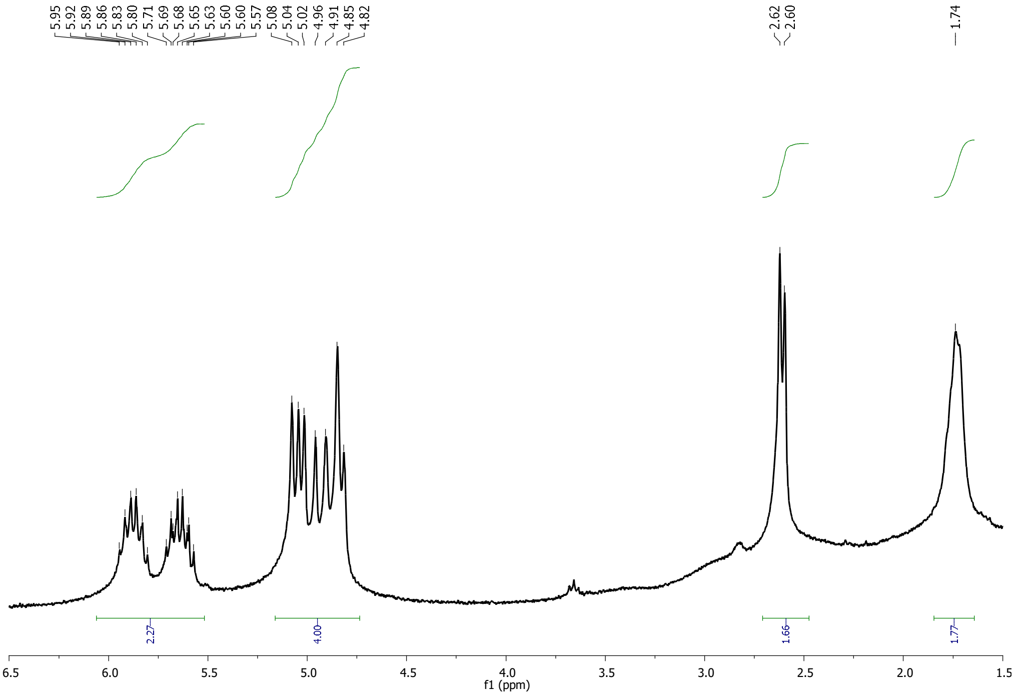

Figure S62. ^{1}H-NMR spectrum.

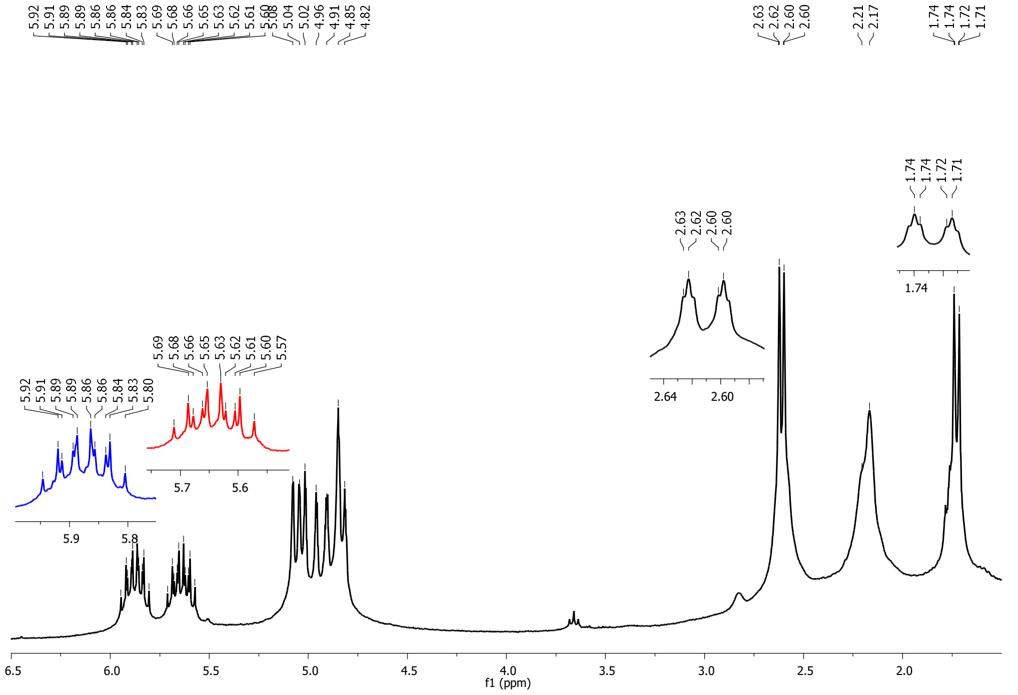

Figure S63. $^{1}H\{^{11}B\}$-NMR spectrum.

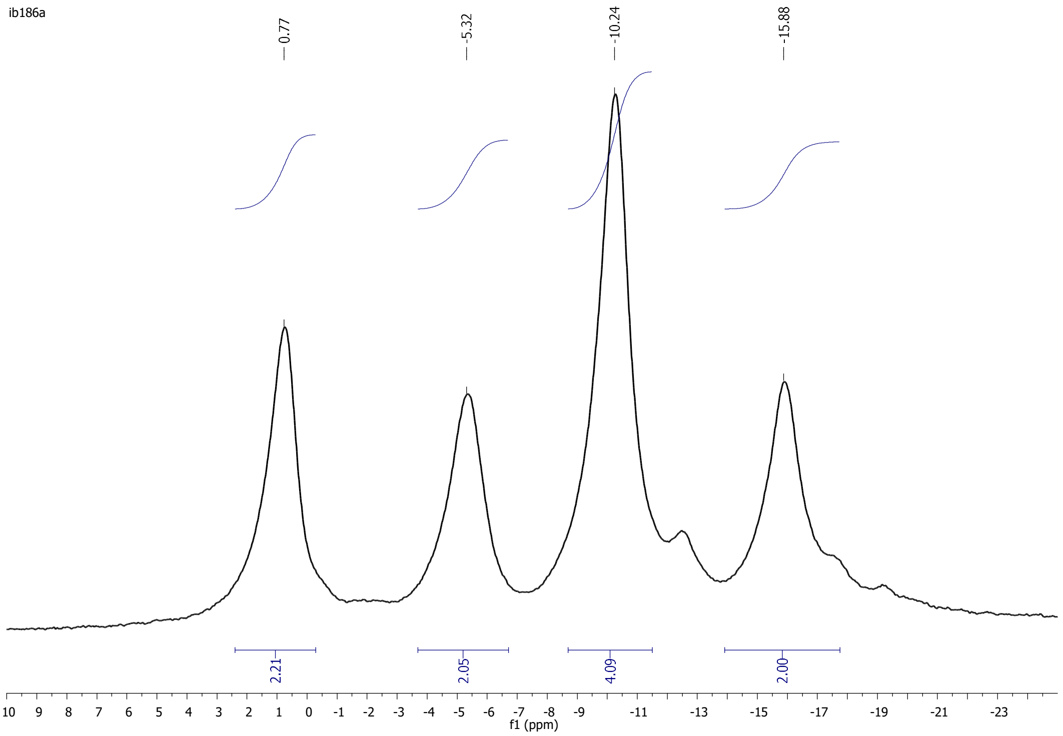

Figure S64. $^{11}B\{^{1}H\}$-NMR spectrum.

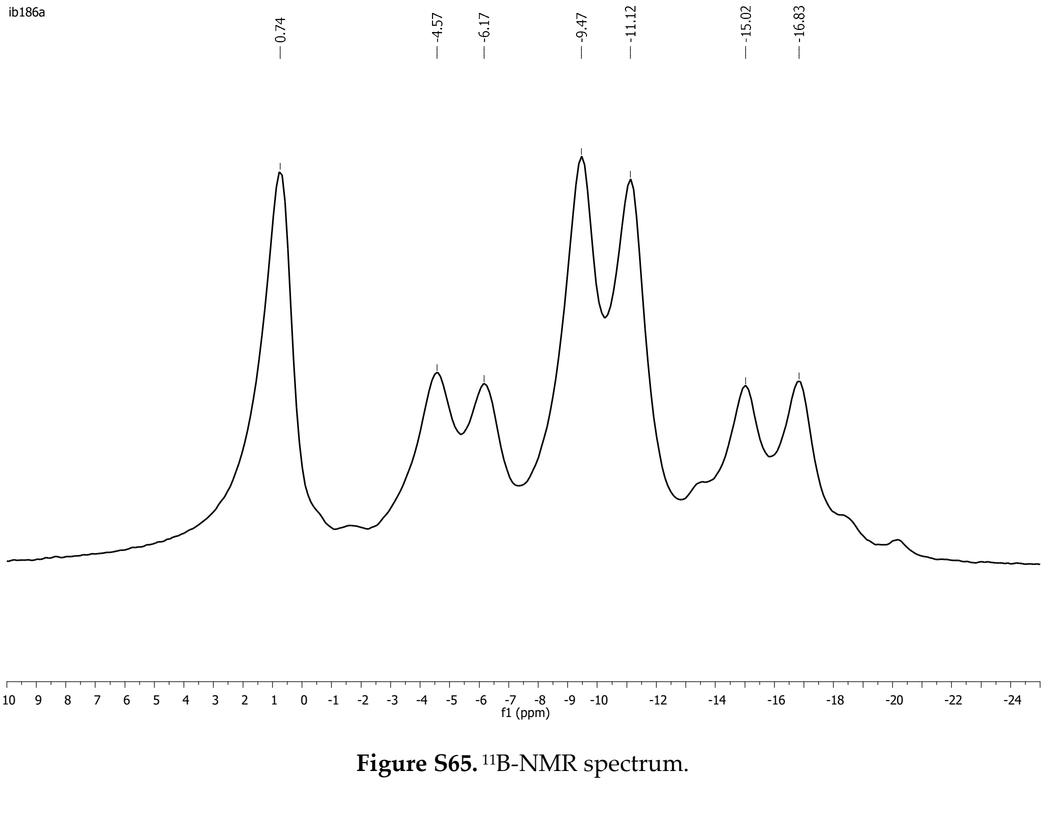

Figure S65. ^{11}B-NMR spectrum.

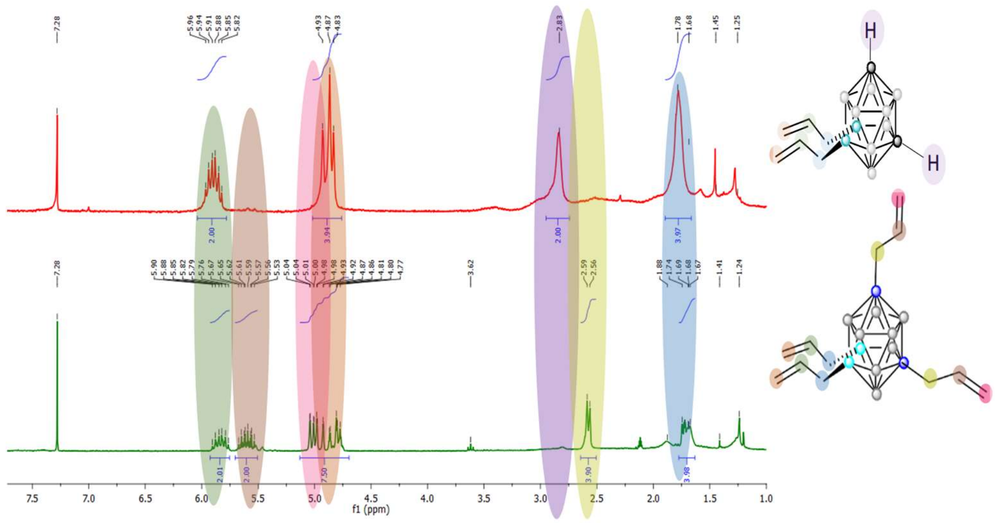

Figure S66. Comparison of ¹H-NMR spectrum of **3** and **10.**

Characterization of 1,7-$(OHCH_2CH_2CH_2)_2$-9,10-$(OHCH_2CH_2CH_2)_2$-1,7-closo-$C_2B_{10}H_8$, 12, in $CDCl_3$.

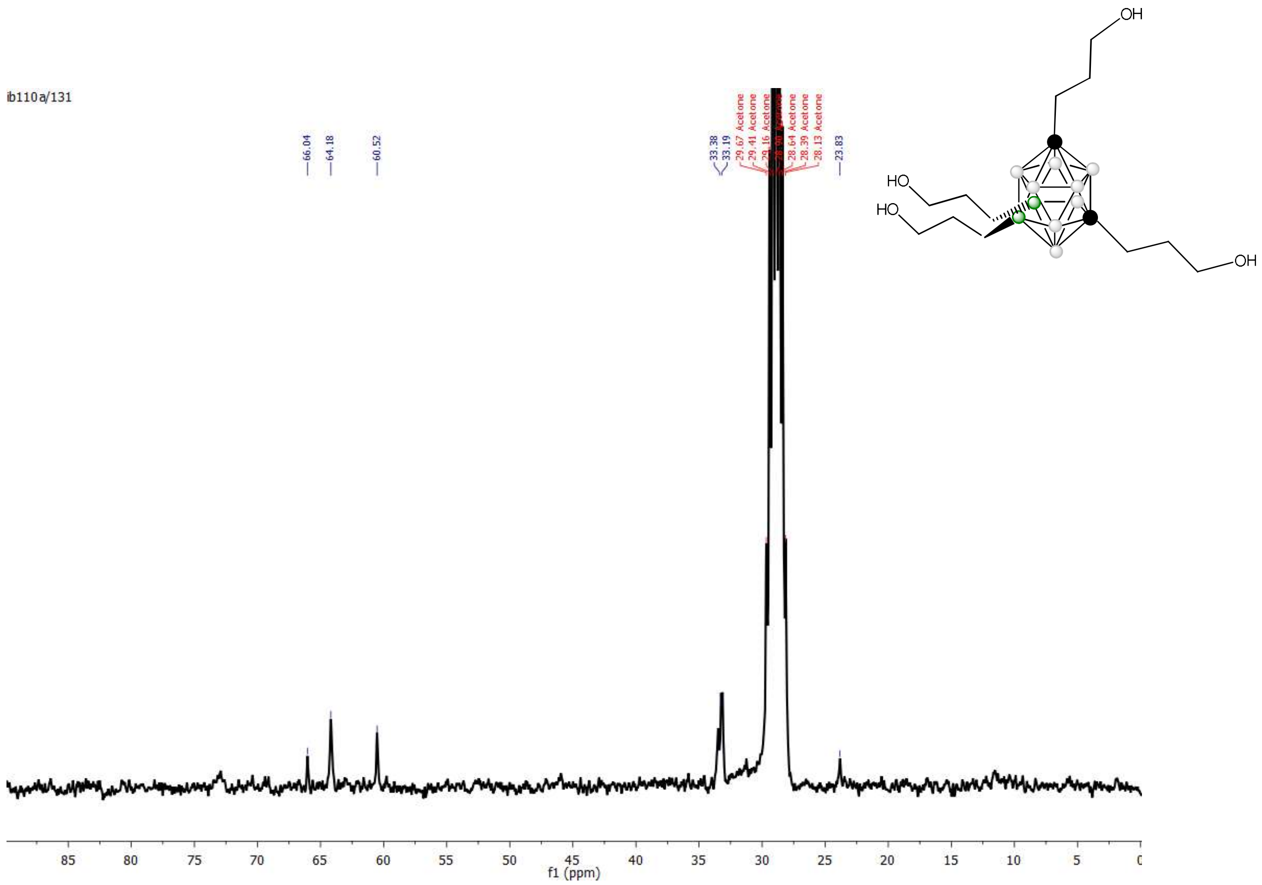

Figure S67. $^{13}C\{^1H\}$-NMR spectrum:.

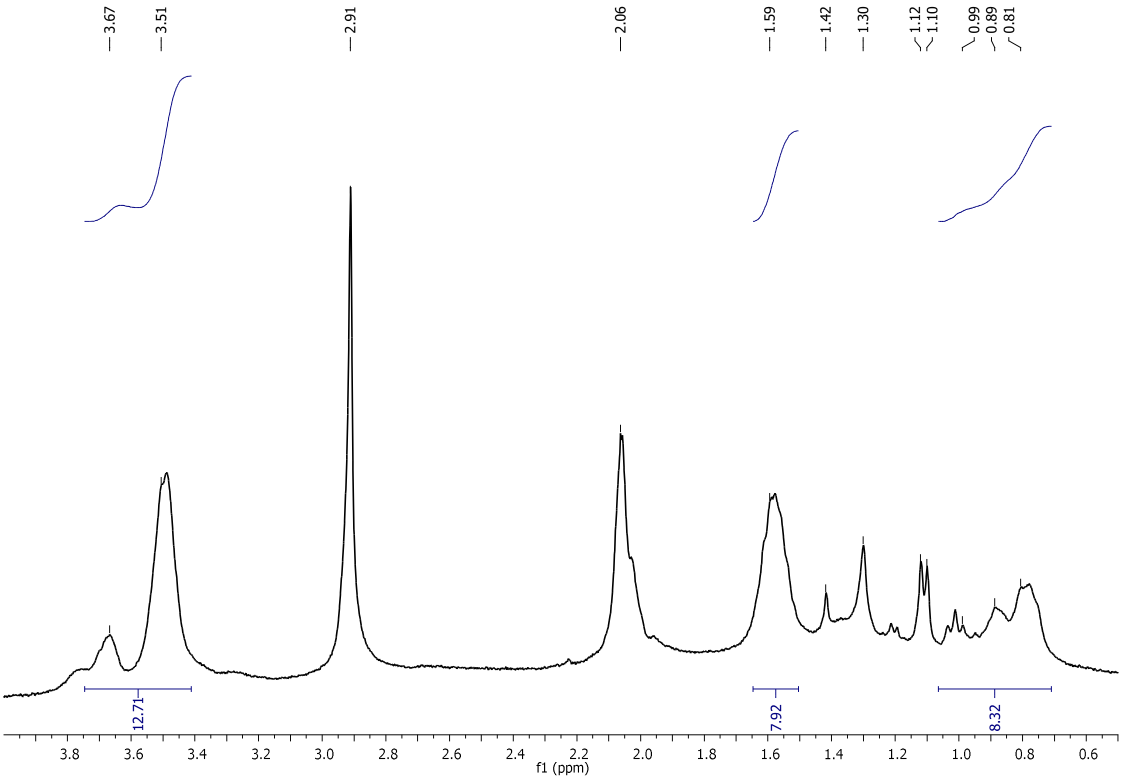

Figure S68. ^{1}H-NMR spectrum.

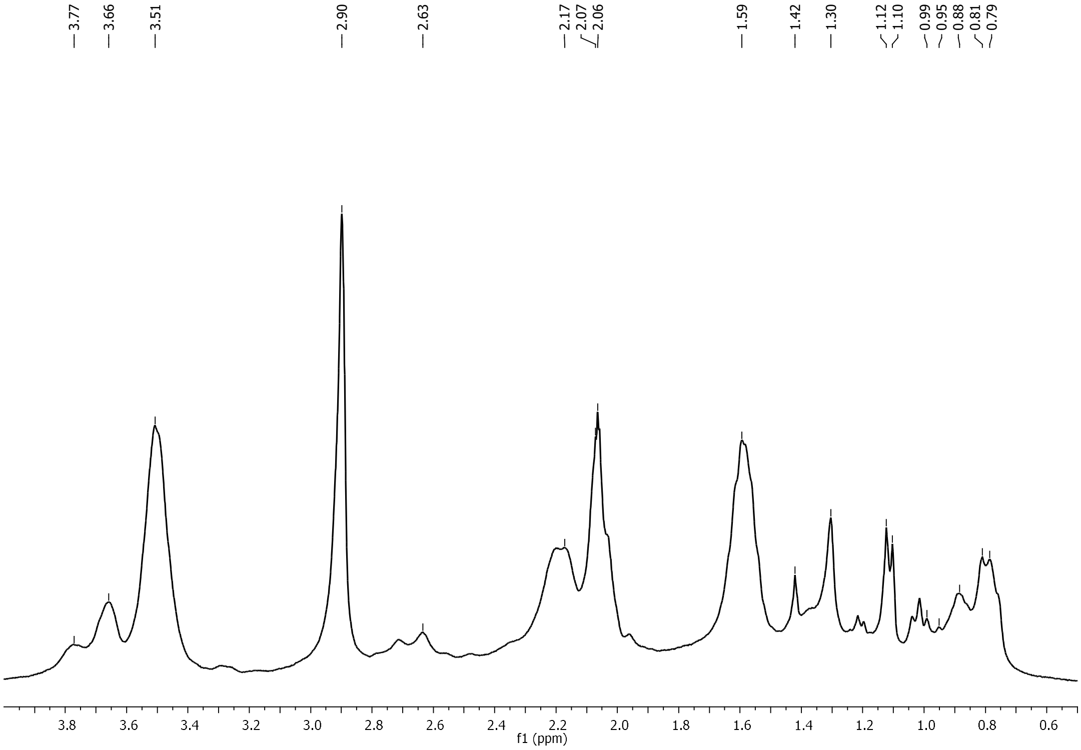

Figure S69. ^{1}H{^{11}B}-NMR spectrum.

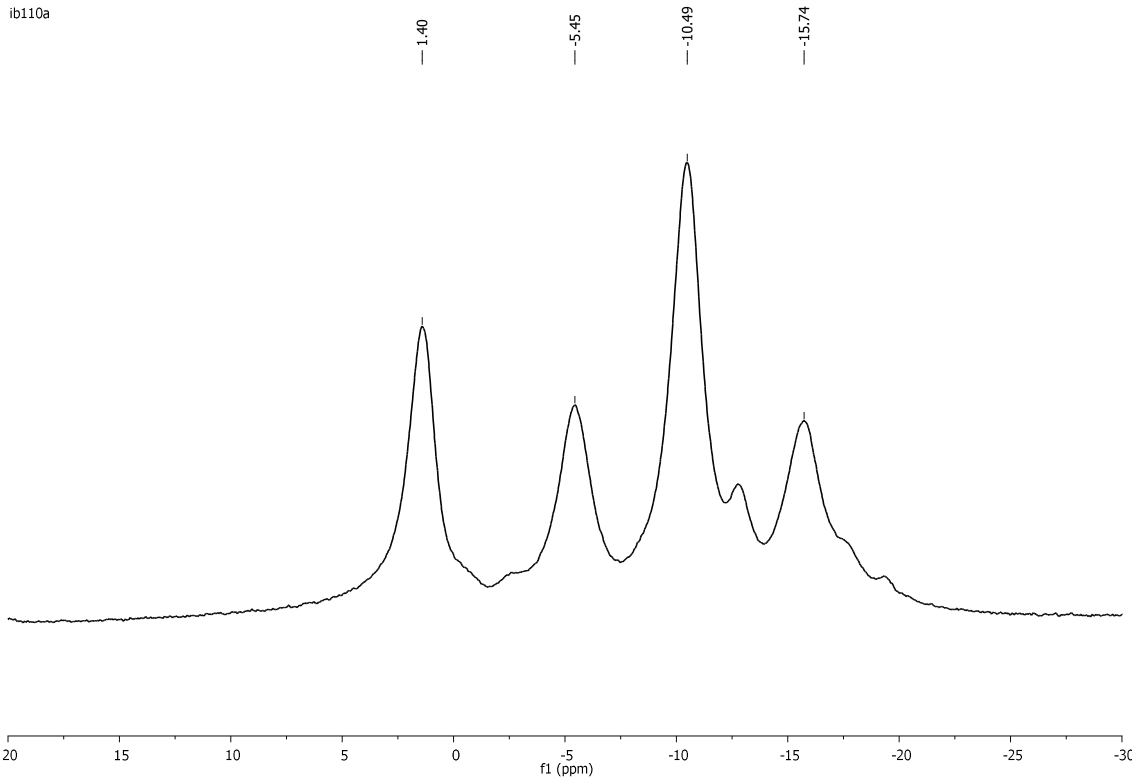

Figure S70. $^{11}B\{^{1}H\}$-NMR spectrum

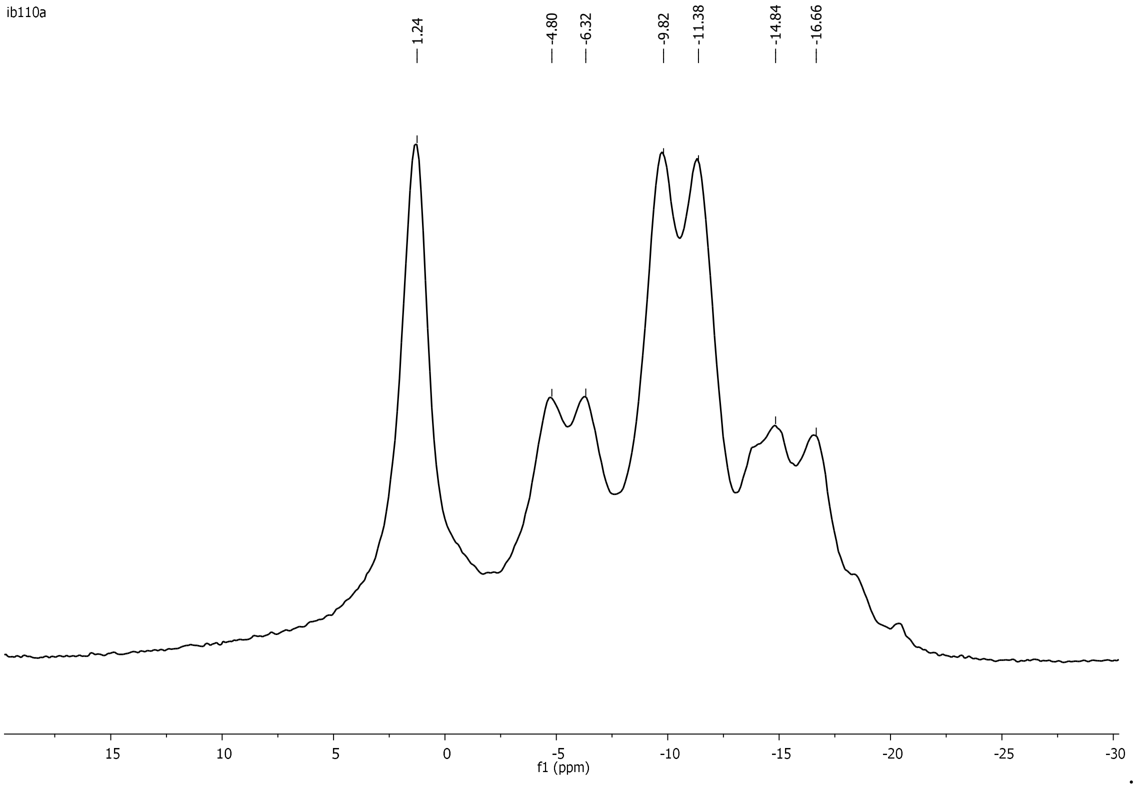

Figure S71. ^{11}B-NMR spectrum.

Characterization of 9,10-($CH_3CH{=}CH$)$_2$-1,7-*closo*-$C_2B_{10}H_{10}$, 10, in d6-acetone.

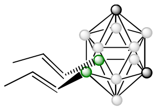

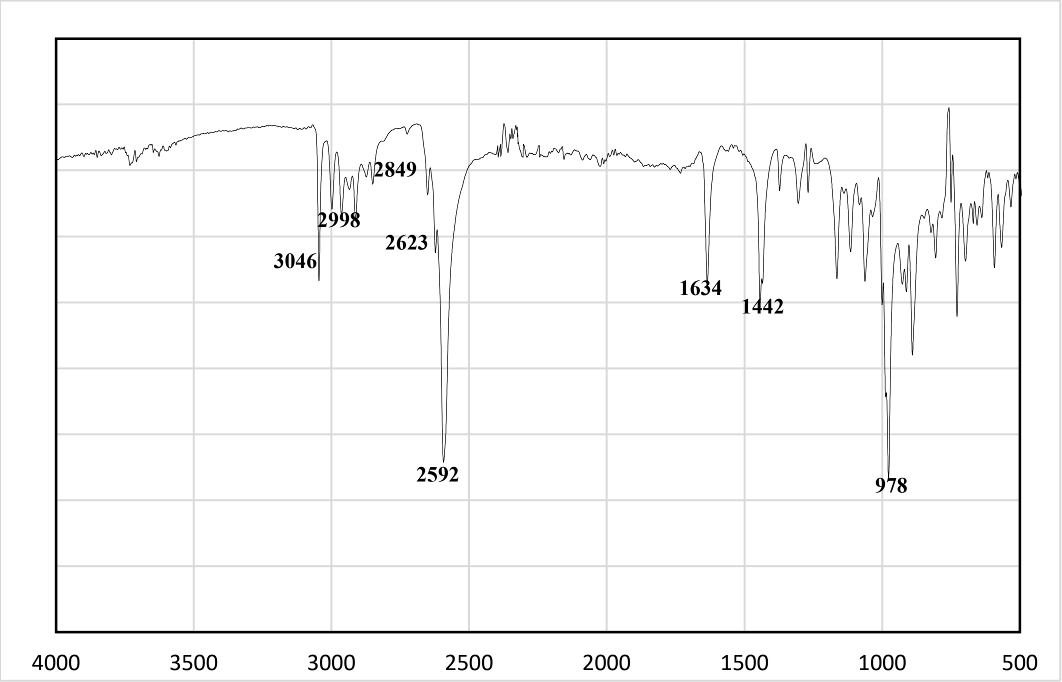

Figure S72. IR-ATR spectrum.

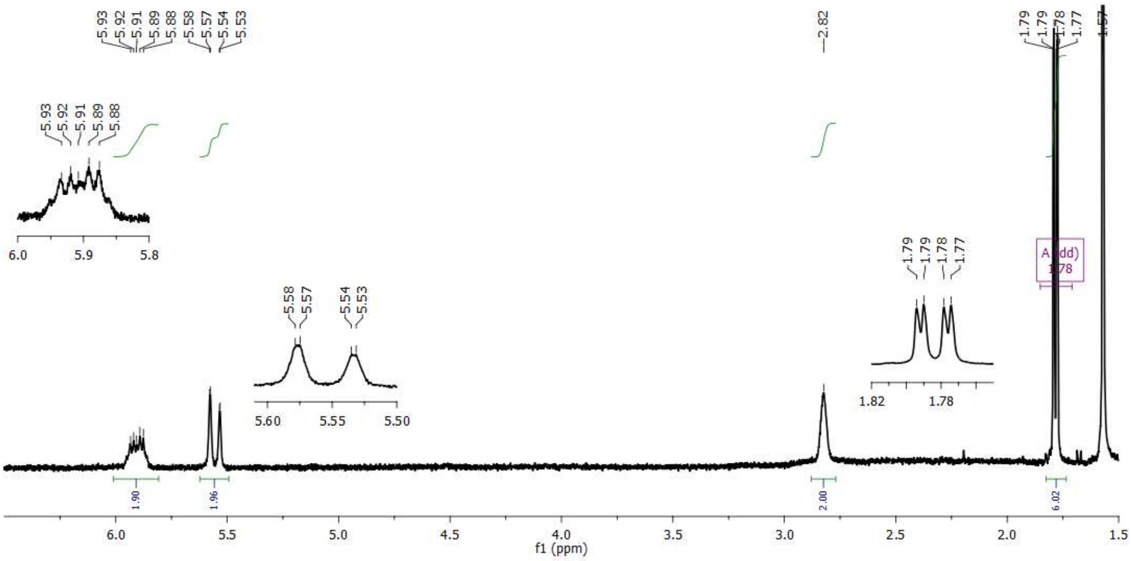

Figure S73. ^1H-NMR spectrum.

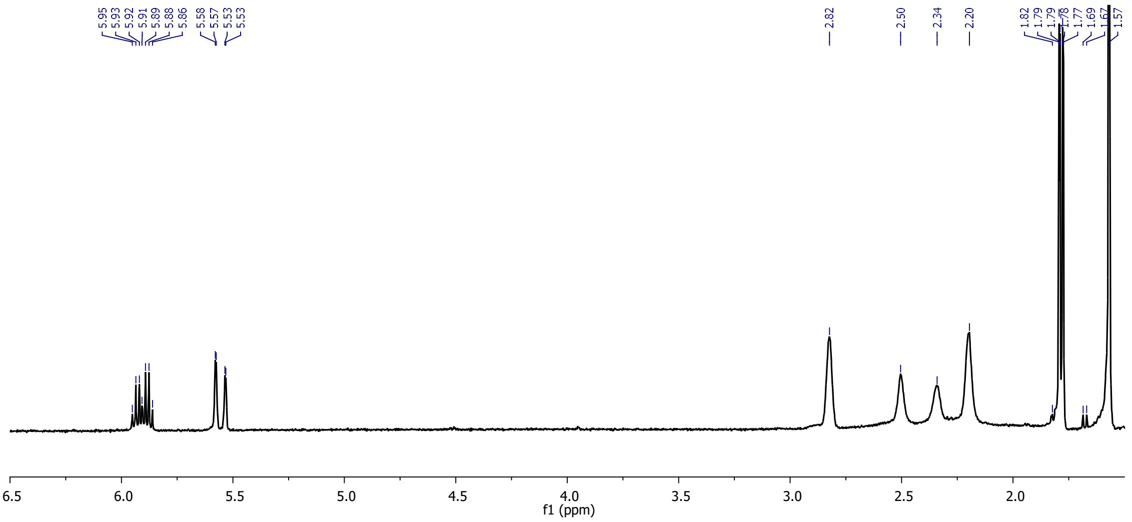

Figure S74. $^{1}H\{^{11}B\}$-NMR spectrum.

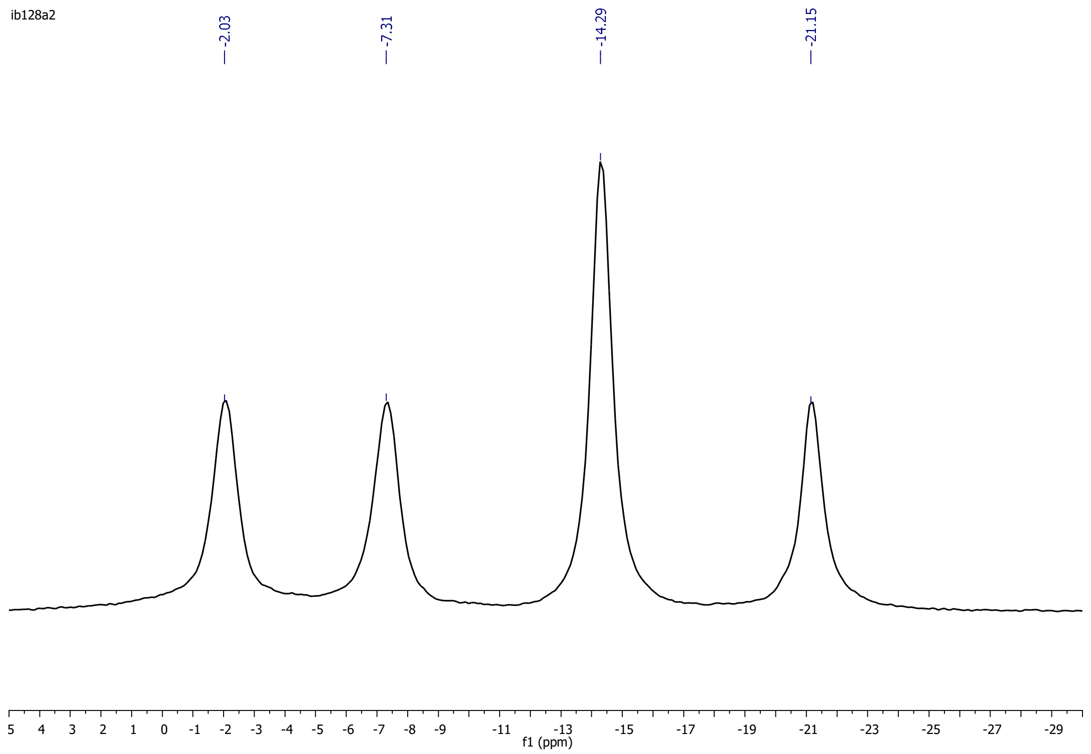

Figure S75. $^{11}B\{^{1}H\}$-NMR spectrum ($CDCl_3$).

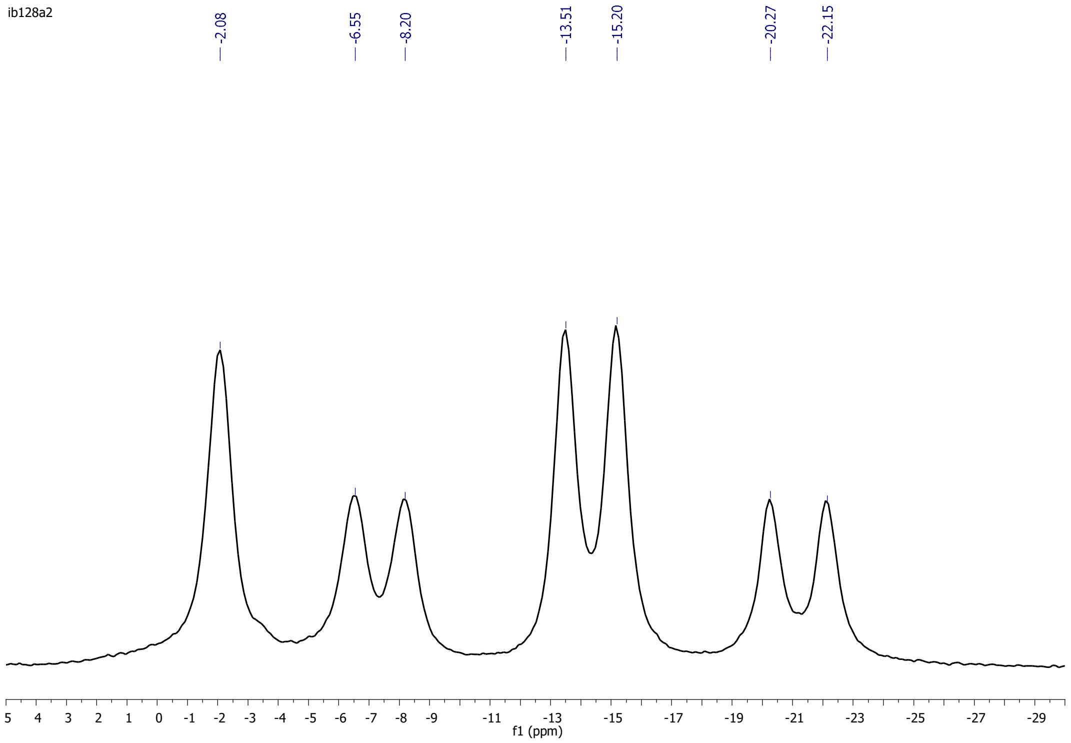

Figure S76. ^{11}B-NMR spectrum ($CDCl_3$).

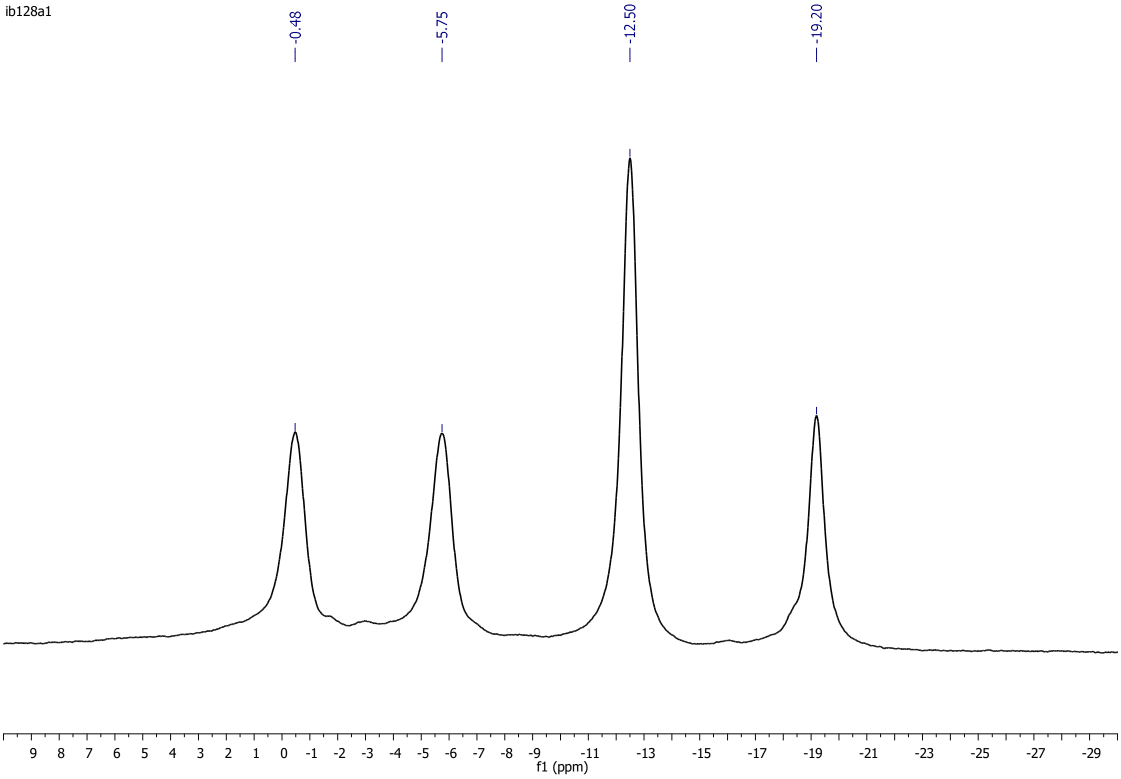

Figure S77. ^{11}B{^{1}H}-NMR spectrum ($CDCOCD_3$).

·

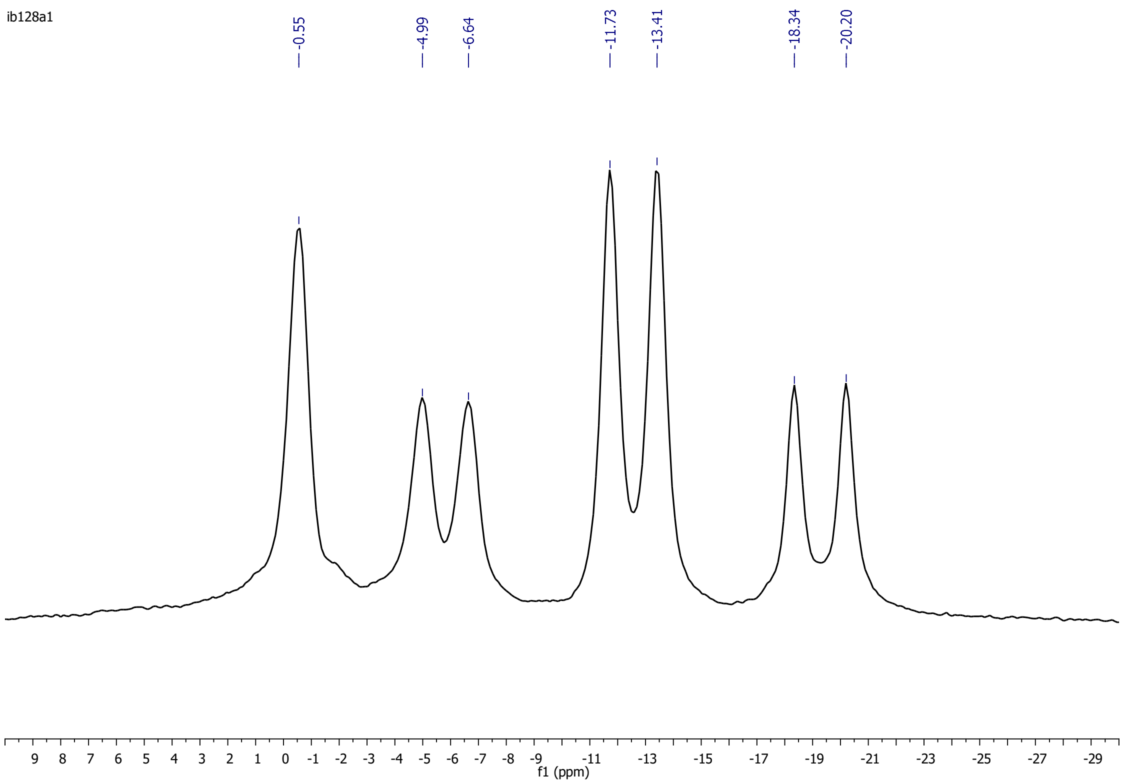

Figure S78. ^{11}B-NMRspectrum ($CDCOCD_3$).

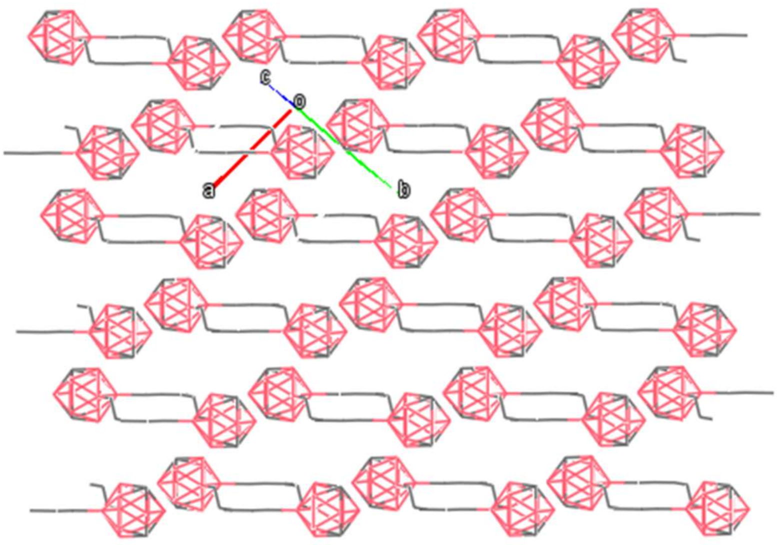

Figure S79. Crystal packing of the 9,10-(CH_3CH=$CH)_2$-1,7-*closo*-$C_2B_{10}H_{10}$.

Table S3. Bond lengths (Å) for 9,10-($CH_3CH{=}CH$)$_2$-1,7-*closo*-$C_2B_{10}H_{10}$.

C5-B9	1.691(2)	C12-B10	1.693(2)
C5-B1	1.704(2)	C12-B8	1.710(2)
C5-B6	1.710(2)	C13-B2	1.569(2)
C12-B9	1.690(2)	C14-C15	1.496(2)
C12-B7	1.703(2)	C16-B3	1.564(2)
C12-B11	1.710(2)	C17-C18	1.493(2)
C13-C14	1.323(2)	B1-B6	1.779(2)
C16-C17	1.315(2)	B1-B3	1.783(2)
B1-B4	1.776(2)	B2-B6	1.785(2)
B1-B2	1.782(2)	B2-B11	1.789(2)
B2-B7	1.787(2)	B3-B7	1.781(2)
B2-B3	1.813(2)	B3-B8	1.793(2)
B3-B4	1.789(2)	B4-B8	1.772(2)
B4-B9	1.764(2)	B6-B10	1.764(2)
B6-B11	1.765(2)	B6-H6	1.12
B7-B8	1.772(2)	B7-B11	1.777(2)
C5-B10	1.695(2)	B8-B9	1.756(3)
C5-B4	1.709(2)	B9-B10	1.784(3)

Table S4. Bond angles (°) for 9,10-($CH_3CH{=}CH)_2$-1,7-*closo*-$C_2B_{10}H_{10}$.

B3-C7-B2	63.59(10)	B3-C7-B12	114.31(12)
B2-C7-B12	114.48(11)	B3C7-B8	62.50(10)
B2-C7-B8	115.46(12)	B1-C7-B8	62.71(9)
B3-C7-B11	115.11(11)	B2-C7-B11	62.40(10)
B12-C7-B11	62.79(9)	B8-C7-B11	115.19(11)
B3-C1-B5	113.90(12)	B3-C1-B2	63.64(10)
B3-C1-B4	62.20(10)	B2-C1-B5	114.27(11)
B5-C1-B4	62.57(10)	B2-C1-B4	115.20(12)
B2-C1-B6	62.38(10)	B3-C1-B6	115.05(12)
B4-C1-B6	115.04(11)	B5-C1-B6	62.76(9)
C17-C16-B10	125.99(14)	C16-C17-C18	125.93(14)
C14-C13-B9	125.80(14)	C13-C14-C15	126.90(15)
C7-B12-B8	58.79(9)	C7-B12-B11	58.79(9)
B8-B12-B11	108.63(12)	C7-B12-B10	105.30(11)
B8-B12-B10	109.42(11)	B11-B12-B10	60.17(9)
C7-B12-B9	105.31(11)	B8-B12-B9	60.37(9)
B11-B1-B9	109.17(11)	B10-B12-B9	61.16(9)
C16-B10-B11	122.47(12)	C13-B10-B12	122.33(12)
C16-B10-B5	123.20(12)	B12-B10-B11	59.82(9)
B11-B10-B5	106.33(11)	B12-B10-B5	105.78(11)
B12-B10-B6	106.57(11)	C16-B10-B6	122.90(12)
B5-B10-B6	59.59(9)	B11-B10-B6	59.19(9)
B12-B10-B9	59.44(9)	C16-B10-B9	121.44(12)
B5-B10-B9	59.27(9)	B11-B10-B9	107.52(11)
C13-B9-B5	122.26(12)	B6-B10-B9	107.37(11)
B5-B9-B12	106.04(11)	C13-B9-B12	122.87(12)
B5-B9-B8	106.44(11)	C13-B9-B8	123.20(13)
C13-B9-B4	122.87(13)	B12-B9-B8	59.63(9)
B12-B9-B4	106.48(12)	B5-B9-B4	59.46(9)
C13-B9-B10	120.97(12)	B8-B9-B4	59.29(9)
B12-B9-B10	59.40(9)	B5-B9-B10	59.64(9)
B4-B9-B10	107.44(11)	B8-B9-B10	107.44(11)
C7-B8-B4	103.97(12)	C7-B8-B3	58.24(9)
C7-B8-B12	58.50(9)	B3-B8-B4	59.56(10)
B4-B8-B12	107.70(12)	B3-B8-B12	107.35(12)
B3-B8-B9	108.19(11)	C7-B8-B9	104.80(11)
B12-B8-B9	60.00(9)	B8-B8-B9	60.47(9)
C7-B11-B2	58.38(9)	C7-B11-B6	104.22(12)
B2-B11-B6	59.93(9)	C7-B11-B12	58.43(9)
B2-B11-B12	107.57(12)	B6-B11-B12	107.78(12)
C7-B11-B10	104.89(11)	B2-B11-B10	108.67(12)
B6-B11-B10	60.53(9)	B12-B11-B10	60.01(9)
C1-B5-B6	58.82(9)	C1-B5-B4	58.91(9)
C1-B5-B9	105.76(11)	B4-B5-B6	108.74(12)
B6-B5-B9	109.36(11)	B4-B5-B9	60.63(9)
B4-B5-B10	109.53(11)	C1-B5-B10	105.49(11)
B9-B5-B10	61.09(9)	B6-B5-B10	60.26(9)
C1-B4-B3	58.35(10)	C1-B4-B8	104.39(12)
B3-B4-B8	59.99(10)	C1-B4-B5	58.52(9)
B3-B4-B5	107.40(12)	B8-B4-B5	107.55(12)

C1-B4-B9	104.92(11)	B3-B4-B9	108.34(12)
B8-B4-B9	60.24(9)	B5-B4-B9	59.92(9)
C1-B3-B4	59.45(9)	C1-B3-C7	101.02(11)
C1-B3-B8	105.57(12)	C7-B3-B4	105.41(12)
B4-B3-B8	60.44(10)	C7-B9-B8	59.25(9)
C7-B3-B2	58.32(9)	C1-B3-B2	58.27(9)
B8-B3-B2	108.45(12)	B4-B3-B2	108.50(12)
C1-B2-C7	100.71(12)	C1-B2-B11	105.10(12)
C7-B2-B6	104.96(11)	B6-B2-B11	60.06(9)
C7-B2-B11	59.22(9)	C7-B2-B3	58.09(9)
C1-B2-B3	58.09(9)	B11-B2-B3	107.99(12)
B6-B2-B3	107.96(12)	C1-B6-B2	58.34(9)
C1-B6-B11	104.35(11)	B2-B6-B11	60.01(9)
C1-B6-B5	58.41(9)	B2-B6-B5	107.38(12)
B11-B6-B5	107.63(11)	C1-B6-B10	105.10(11)
B2-B6-B10	108.54(11)	B11-B6-B10	60.28(9)
B5-B6-B10	60.15(9)		

Theoretical Calculations:

All calculations were carried out with the Gaussian 09 program package[1] at B3LYP/6-311+G** or B3LYP-D3/6-311+G** level of theory as it was implemented in G09. Full geometry optimization calculations were performed and harmonic vibrational frequencies were calculated to establish the nature of the stationary points obtained, as characterized by none negative eigenvalue of the Hessian for minima structures. In case of the calculations of the protonaffinity *single point* calculations were performed using polarizable continuum model (solvent=tetrahydrofuran). For the visualization of the molecules and orbitals the MOLDEN[2] was used.

XYZ coordinates and total energies of the investigated systems

Table S5. XYZ coordinates and total energies of *o*-carborane.

E(B3LYP-D3/6-311+G**)=-332.189573

C	-0.000374	0.000807	-0.000461
B	-0.000161	0.000794	1.697876
B	1.518715	0.000904	0.758650
B	1.253086	-0.914037	-0.745342
B	0.860588	-2.594439	-0.306457
B	0.907397	-2.724655	1.466748
B	2.086399	-1.681002	0.608521
B	1.314863	-1.117622	2.126256
B	-0.387440	-1.681513	2.138395
B	-1.226524	-0.914753	0.787889
B	-0.658133	-2.594727	0.632980
C	-0.371918	-1.463987	-0.601391
H	-1.465547	-3.433705	0.441036
H	-0.939821	-1.471897	-1.520287
H	-0.979309	-1.937699	3.129231
H	3.237264	-1.937178	0.521418
H	1.932372	-0.970114	3.124048
H	1.231211	-3.734835	1.989589
H	1.624098	-0.571596	-1.809508
H	1.050398	-3.433110	-1.114681
H	2.169805	0.983254	0.697529
H	-0.345930	0.982841	2.253426
H	-2.344424	-0.572242	0.645163
H	-0.351073	0.851081	-0.567018

Table S6. XYZ coordinates and total energies of *m*-carborane.

E(B3LYP-D3/6-311+G**)= -332.2153305

C	0.000000	0.000000	0.000000
B	0.000000	0.000000	1.694042
B	1.707172	0.000000	2.155344
B	1.156214	0.987715	0.791537
B	2.679059	0.072856	0.664636
B	2.354484	-1.414871	-0.283242
B	1.183078	-2.401712	0.625508
B	2.442677	-1.483693	1.492224
C	0.810506	-1.454028	2.005449
B	-0.324120	-1.484745	0.748521
B	0.632290	-1.413085	-0.736984
B	1.554092	0.112955	-0.711691
H	2.003724	0.396302	3.226191
H	3.724937	0.623270	0.641531
H	1.134941	-3.578397	0.695646
H	3.170542	-1.916420	-0.975933
H	3.238241	-2.068531	2.138680
H	-1.400598	-1.951036	0.844990
H	0.549825	-1.976553	2.915134
H	0.151983	-1.814574	-1.736964
H	1.684931	0.722353	-1.713900
H	1.020970	2.159679	0.792113
H	-0.826540	0.493288	-0.491893
H	-0.883871	0.415051	2.351367

Table S7. XYZ coordinates and total energies of compound 3.

E(B3LYP-D3/6-311+G**, PCM=THF) = -565.7305145
E(B3LYP-D3/6-311+G**)= -565.726117

C	-0.014610	0.002207	0.010772
B	-0.003030	-0.005328	1.706226
B	1.707616	-0.006877	2.153447
B	1.147537	0.984505	0.801190
B	1.535568	0.120048	-0.703093
B	2.353692	-1.415499	-0.303293
B	1.174045	-2.395903	0.618364
B	2.435175	-1.481505	1.476922
B	2.687092	0.086308	0.659544
C	4.091536	0.860367	0.618728
C	5.200409	0.143117	1.330825
C	5.806183	0.556002	2.443132
B	0.616438	-1.403554	-0.734532
B	-0.333930	-1.485234	0.755569
C	0.810205	-1.458918	2.004873
C	3.462035	-2.118258	-1.228322
C	3.891765	-1.294897	-2.408018
C	5.111151	-0.795526	-2.603401
H	-0.843154	0.498984	-0.474114
H	0.557785	-1.986871	2.913701
H	3.240924	-2.069104	2.107520
H	2.019263	0.386299	3.221292
H	-1.408761	-1.954449	0.854464
H	-0.883039	0.407006	2.370145
H	1.135993	-3.574320	0.675277
H	1.666968	0.737062	-1.700783
H	1.018135	2.157758	0.808189
H	0.138687	-1.803917	-1.737334
H	4.368749	0.997721	-0.432803
H	3.967028	1.861305	1.045393
H	4.333505	-2.372864	-0.616615
H	3.054084	-3.071312	-1.588347
H	3.117151	-1.082360	-3.143266
H	5.346604	-0.192180	-3.473059
H	5.916767	-0.970615	-1.896037
H	5.503646	-0.808201	0.896662
H	6.589404	-0.027014	2.914989
H	5.536879	1.494019	2.920400

Table S8. XYZ coordinates and total energies of compound 3 deprotonated in allylic position.

E(B3LYP-D3/6-311+G**, PCM=THF) =-565,18499
E(B3LYP-D3/6-311+G**)= -565.122175

C	0.087363	0.005107	-0.051226
B	-0.020739	-0.069146	1.650809
B	1.672777	-0.021765	2.311739
C	2.106262	-0.655157	3.606738
C	1.842291	-1.993302	3.979856
C	2.178205	-2.678627	5.123725
B	-0.370154	1.412899	0.746456
B	0.645782	1.471839	2.193717
B	2.339579	1.533563	1.643285
B	2.363897	1.547893	-0.158653
B	1.702902	0.028834	-0.697521
B	1.277724	-0.920505	0.774581
B	2.750807	0.069913	0.715261
C	4.194814	-0.620199	0.776019
C	5.342395	0.321415	0.604932
C	6.269762	0.277869	-0.354791
B	0.682364	1.467407	-0.700643
C	1.068888	2.278305	0.748160
H	-0.666113	-0.512728	-0.627165
H	1.006497	3.356996	0.736040
H	3.105168	2.246042	2.202117
H	3.110902	2.253364	-0.745518
H	-1.435942	1.911577	0.641724
H	0.231796	1.999095	-1.653975
H	0.268783	2.121256	3.107983
H	1.230845	-2.098612	0.660039
H	1.910644	-0.518860	-1.727187
H	-0.929991	-0.653138	2.130822
H	4.242278	-1.075584	1.778087
H	4.260919	-1.433265	0.044347
H	2.647518	-0.041261	4.329882
H	1.278990	-2.574652	3.242011
H	1.890087	-3.714947	5.265731
H	2.746082	-2.207648	5.922823
H	5.394567	1.127003	1.337125
H	7.063406	1.016007	-0.416977
H	6.259365	-0.498530	-1.115676

Table S9. XYZ coordinates and total energies of compound 3 deprotonated at cluster carbon atom position.

E(B3LYP-D3/6-311+G**, PCM=THF) = -565.2000877
E(B3LYP-D3/6-311+G**)= -565.141006

C	-0.039343	-0.014849	0.011823
B	-0.014669	-0.005525	1.741487
B	1.669808	0.012898	2.164841
B	1.118885	0.975138	0.768889
B	1.497651	0.062345	-0.708592
B	2.296513	-1.456110	-0.241701
B	1.119445	-2.387129	0.757264
B	2.370774	-1.462451	1.547178
B	2.644746	0.068198	0.649344
C	4.065659	0.827065	0.525115
C	5.158902	0.227714	1.352466
C	5.823253	0.830292	2.340500
B	0.566608	-1.453885	-0.658991
B	-0.351938	-1.476705	0.876746
C	0.756903	-1.447566	2.148552
C	3.411065	-2.203190	-1.142705
C	3.688173	-1.542870	-2.456011
C	4.845072	-0.996206	-2.836600
H	-0.870029	0.468276	-0.487389
H	3.245278	-2.014807	2.135952
H	2.040893	0.520257	3.174286
H	-1.457580	-1.907388	0.910698
H	-0.905537	0.512813	2.330345
H	1.110143	-3.577937	0.764340
H	1.641597	0.638028	-1.737162
H	0.999824	2.156208	0.728436
H	0.079239	-1.893910	-1.649374
H	4.364675	0.790576	-0.530731
H	3.945930	1.885551	0.783586
H	4.340777	-2.296627	-0.570650
H	3.054961	-3.225786	-1.326009
H	2.838653	-1.478828	-3.135660
H	4.954198	-0.498309	-3.794975
H	5.716659	-1.010208	-2.187561
H	5.398223	-0.811522	1.131265
H	6.583289	0.312112	2.916855
H	5.606258	1.857272	2.622759

Table S10. XYZ coordinates and total energies of *o*-carborane analogue of compound 3 deprotonated at the allylic position.

E(B3LYP-D3/6-311+G**, PCM=THF)= -565.1603823
E(B3LYP-D3/6-311+G**)= -565.094071

C	-0.051236	0.025627	0.056417
C	-0.001683	-0.122777	1.696887
B	1.589201	-0.205183	2.272399
B	2.285064	-1.770144	1.650188
C	3.333507	-2.575137	2.371299
C	3.352997	-2.825080	3.760978
C	4.246636	-3.545195	4.520925
B	-0.534413	-1.478725	0.748715
B	0.472459	-1.415230	-0.684633
B	0.880738	-2.493953	0.645432
B	0.538879	-1.658481	2.169030
B	1.165999	0.881478	0.912553
B	2.589036	-0.131632	0.818986
B	2.188159	-1.580599	-0.175035
C	3.237031	-2.344782	-1.129689
C	4.530469	-1.618878	-1.321989
C	5.009661	-1.158765	-2.479967
B	1.537865	0.041573	-0.584824
H	-1.692883	-1.718200	0.764242
H	1.030169	2.051341	1.024584
H	3.664437	0.364709	0.831049
H	1.727799	0.673121	-1.568630
H	-0.780396	0.398801	2.232645
H	-0.847722	0.629722	-0.350566
H	1.802650	0.301684	3.320243
H	0.763651	-3.668557	0.534077
H	0.006564	-1.697443	-1.737755
H	0.056450	-2.113301	3.149322
H	3.441927	-3.309862	-0.646964
H	2.780978	-2.561826	-2.103205
H	4.151144	-3.007122	1.789031
H	2.527666	-2.374397	4.323359
H	4.124380	-3.643004	5.594565
H	5.103767	-4.041639	4.071825
H	5.097634	-1.436882	-0.410826
H	5.946482	-0.613088	-2.533801
H	4.470255	-1.303681	-3.413003

Table S11. XYZ coordinates and total energies of *o*-carborane analogue of compound 3 deprotonated at the cluster carbon atom position.

E(B3LYP-D3/6-311+G**, PCM=THF)= -565.1899989
E(B3LYP-D3/6-311+G**)= -565.132790

C	0.044269	-0.019678	0.022147
B	-0.037683	-0.008668	1.736073
C	1.447942	-0.051115	0.961383
B	2.353253	1.385137	0.945594
B	1.393292	0.903759	2.360180
B	-0.177482	1.699650	2.265974
B	-0.190808	2.718612	0.790622
C	-1.017144	4.100221	0.643543
C	-1.713484	4.544045	1.889800
C	-3.030823	4.677971	2.058598
B	1.486501	0.751666	-0.506636
B	1.381974	2.479457	-0.038885
B	1.308516	2.575855	1.756479
C	1.927382	3.783291	2.640506
C	2.387669	4.963858	1.843983
C	1.896154	6.202614	1.912213
B	-0.053105	1.584326	-0.592834
B	-0.995781	1.116576	0.798928
H	2.106749	0.318448	-1.420658
H	-0.435262	-0.952956	2.334254
H	-0.773321	2.001635	3.249904
H	-2.171278	0.960234	0.722946
H	1.954899	-1.005717	0.981573
H	1.941736	0.526397	3.343946
H	1.880099	3.334909	-0.699027
H	-0.563092	1.764031	-1.651867
H	3.539110	1.324847	0.988957
H	-0.302898	4.878456	0.346775
H	-1.743553	4.003180	-0.171676
H	1.193739	4.102189	3.388811
H	2.783693	3.379207	3.197718
H	3.175263	4.760422	1.119463
H	2.261489	6.997774	1.269959
H	1.089313	6.454962	2.595050
H	-3.457044	4.975796	3.011697
H	-3.727834	4.468646	1.251405
H	-1.064081	4.745008	2.740757

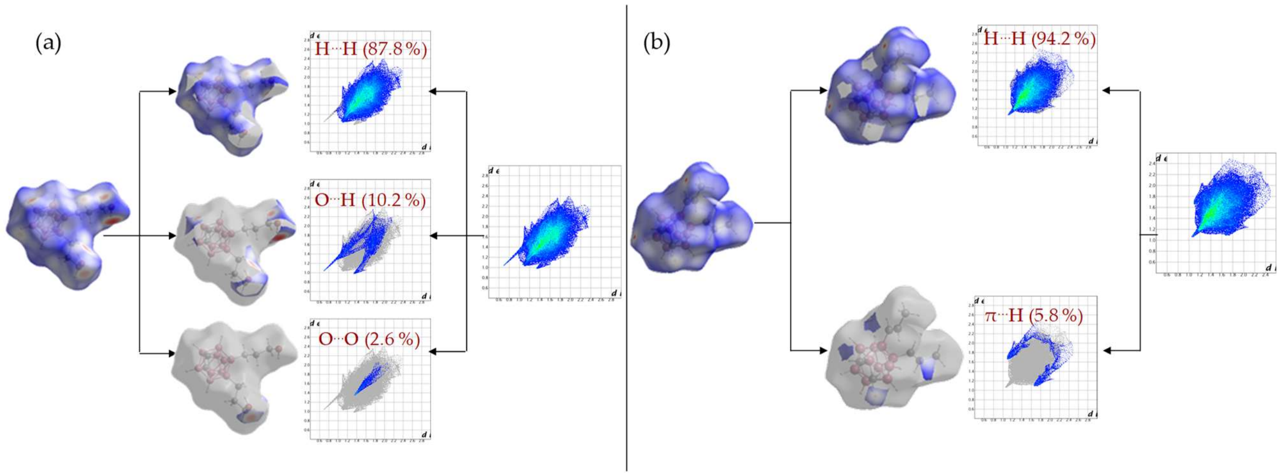

Figure S80. Two-dimensional fingerprint plots overall plot and those delineated into (a) H…H and O…H/H…O and O…O for **4**; (b) H…H and π…H/H…π for **12**.

References:

1. Gaussian 09, Revision B.01, M. J. Frisch, G. W. Trucks, H. B. Schlegel, G. E. Scuseria, M. A. Robb, J. R. Cheeseman, G. Scalmani, V. Barone, B. Mennucci, G. A. Petersson, H. Nakatsuji, M. Caricato, X. Li, H. P. Hratchian, A. F. Izmaylov, J. Bloino, G. Zheng, J. L. Sonnenberg, M. Hada, M. Ehara, K. Toyota, R. Fukuda, J. Hasegawa, M. Ishida, T. Nakajima, Y. Honda, O. Kitao, H. Nakai, T. Vreven, J. A. Montgomery, Jr., J. E. Peralta, F. Ogliaro, M. Bearpark, J. J. Heyd, E. Brothers, K. N. Kudin, V. N. Staroverov, T. Keith, R. Kobayashi, J. Normand, K. Raghavachari, A. Rendell, J. C. Burant, S. S. Iyengar, J. Tomasi, M. Cossi, N. Rega, J. M. Millam, M. Klene, J. E. Knox, J. B. Cross, V. Bakken, C. Adamo, J. Jaramillo, R. Gomperts, R. E. Stratmann, O. Yazyev, A. J. Austin, R. Cammi, C. Pomelli, J. W. Ochterski, R. L. Martin, K. Morokuma, V. G. Zakrzewski, G. A. Voth, P. Salvador, J. J. Dannenberg, S. Dapprich, A. D. , O. Farkas, J. B. Foresman, J. V. Ortiz, J. Cioslowski, D. J. Fox, Gaussian Inc., Wallingford CT, **2010.**

2. G. Schaftenaar, J. H. Noordik, *J. Comput. Aided Mol. Design* 2000, **14**, 123.
